# Supplementary material for: Domino Nitro Reduction-Friedländer Heterocyclization for the Preparation of Quinolines
Source: Molecules. 2022 Jun 27;27(13):4123. doi: 10.3390/molecules27134123 (PMC9268355; doi:10.3390/molecules27134123)

Domino nitro reduction-Friedländer heterocyclization for the preparation of quinolines

Kwabena Fobi and Richard A. Bunce*

Department of Chemistry, Oklahoma State University, Stillwater, OK 74078-3071, U.S.A.; kfobi@ok-state.edu (K.F.)
* Correspondence: rab@okstate.edu; Tel. +1-405-744-5952

Supplemental Information

General methods 3

General procedure for domino reduction-heterocyclization 3

^{1}H and ^{13}C NMRs for
- 3.3.1. Ethyl 2-methylquinoline-3-carboxylate (Table 1) 4
- 3.3.2. Ethyl 2-(trifluoromethyl) quinoline-3-carboxylate (Table 1) 5
- 3.3.3. Methyl 2-ethylquinoline-3-carboxylate (Table 1) 6
- 3.3.4. Methyl 2-pentylquinoline-3-carboxylate (Table 1) 7
- 3.3.5. Methyl 2-isopropylquinoline-3-carboxylate (Table 1) 8
- 3.3.6. Methyl 2-(*tert*-butyl) quinoline-3-carboxylate (Table 1) 9
- 3.3.7. Ethyl 2-phenylquinoline-3-carboxylate (Table 1) 10
- 3.3.8. Methyl 2-benzylquinoline-3-carboxylate (Table 1) 11
- 3.3.9. Methyl 2-phenethylquinoline-3-carboxylate (Table 1) 12
- 3.3.10. Methyl 2-(phenoxymethyl)quinoline-3-carboxylate (Table 1) 13
- 3.3.11. 2-Methyl-3-(phenylsulfonyl)quinoline (Table 1) 14
- 3.3.12. 2-Methylquinoline-3-carbonitrile (Table 1) 15
- 3.3.13. 2-Phenylquinoline-3-carbonitrile (Table 1) 16
- 3.3.14. 3,3-Dimethyl-3,4-dihydroacridin-1(2*H*)-one (Table 1) 17
- 3.3.15. 1-(2-Methylquinolin-3-yl)ethan-1-one (Table 1) 18
- 3.3.16. 2-Methylquinolin-3-yl)(phenyl)methanone (Table 1) 19
- 3.3.17. 2-Methyl-3-phenylquinoline (Table 1) 20
- 3.3.18. 2,3-Diphenylquinoline (Table 1) 21
- 3.3.19. 5,6-Dihydrobenzo[*a*]acridine (Table 1) 22

- 3.4.1. Ethyl 6-fluoro-2-(trifluoromethyl)quinoline-3-carboxylate (Table 2) 23
- 3.4.2. Methyl 6-fluoro-2-isopropylquinoline-3-carboxylate (Table 2) 24
- 3.4.3. Ethyl 6-fluoro-2-phenylquinoline-3-carboxylate (Table 2) 25
- 3.4.4. Methyl 2-benzyl-6-fluoroquinoline-3-carboxylate (Table 2) 26
- 3.4.5. 6-Fluoro-2-methyl-3-(phenylsulfonyl)quinoline (Table 2 5) 27
- 3.4.6. 6-Fluoro-2-phenylquinoline-3-carbonitrile (Table 2) 28
- 3.4.7. 7-Fluoro-3, 3-dimethyl-3, 4-dihydroacridin-1(2H)-one (Table 2) 29

- 3.5.1. Ethyl 6-methoxy-2-(trifluoromethyl)quinoline-3-carboxylate (Table 3) 30
- 3.5.2. Methyl 2-isopropyl-6-methoxyquinoline-3-carboxylate (Table 3) 31
- 3.5.3. Ethyl 6-methoxy-2-phenylquinoline-3-carboxylate (Table 3) 32
- 3.5.4. Methyl 2-benzyl-6-methoxyquinoline-3-carboxylate (Table 3) 33
- 3.5.5. 6-Methoxy-2-methyl-3-(phenylsulfonyl)quinoline (Table 3) 34

3.5.6. 6-Methoxy-2-phenylquinoline-3-carbonitrile (Table 3) 35
3.5.7. 7-Methoxy-3,3-dimethyl-3,4-dihydroacridine-1(2*H*)-one (Table 3) 36

3.6.1. 4-Methyl-3-propionylquinolin-2(1*H*)-one (Table 4) 37
3.6.2. 3-Benzoyl-4-methylquinolin-2(1*H*)-one (Table 4) 38
3.6.3. 4-Methyl-2-phenylquinoline-3-carbonitrile (Table 4) 39
3.6.4. 1-(2,4-Dimethylquinolin-3-yl)ethan-1-one (Table 4) 40
3.6.5. 3,3,9-Trimethyl-3,4-dihydroacridin-1(2*H*)-one (Table 4) 41
3.6.6. 2,4-Dimethyl-3-phenylquinoline (Table 4) 42
3.6.7. 12-Methyl-5,6-dihydrobenzo[*a*]acridine (Table 4) 43

3.7.1. Methyl 2-ethyl-4-phenylquinoline-3-carboxylate (Table 5) 44
3.7.2-3.7.3. 3-Benzoyl-4-phenylquinolin-2(1*H*)-one (Table 5) 45
3.7.4. (2-Methyl-4-phenylquinolin-3-yl)ethan-1-one (Table 5) 46
3.7.5. 3,3-Dimethyl-9-phenyl-3,4-dihydroacridin-1(2*H*)-one (Table 5) 47
3.7.6. 2-Methyl-3,4-diphenylquinoline (Table 5) 48
3.7.7. 3-Butyl-2,4-diphenylquinoline (Table 5) 49
3.7.8. 12-Phenyl-5,6-dihydrobenzo[*a*]acridine (Table 5) 50

General Methods.

Unless otherwise indicated, all reactions were performed under dry N_2 in oven-dried glassware. All reagents and solvents were used as received. 2-Nitrobenzophenone (**1e**) was prepared using the literature procedure [29,30]. Reactions were monitored by thin layer chromatography on Analtech No 21521 silica gel GF plates (Newark, DE, USA). Preparative separations were performed by flash chromatography on silica gel (Davisil®, grade 62, 60–200 mesh) containing 0.5% of UV-05 UV-active phosphor (both from Sorbent Technologies, Norcross, GA, USA) slurry packed into quartz columns. Band elution for all chromatographic separations was monitored using a hand-held UV lamp (Fisher Scientific, Pittsburgh, PA, USA). Wash solutions used in work-up procedures were all aqueous. Melting points were obtained using a MEL-TEMP apparatus (Cambridge, MA, USA) and are uncorrected. FT-IR spectra were run as thin films on NaCl disks using a Nicolet iS50 spectrophotometer (Madison WI, USA). ^1H- and ^{13}C-NMR spectra were measured using a Bruker Avance 400 system (Billerica, MA, USA) at 400 MHz and 101 MHz, respectively, in the indicated solvents containing 0.05% $(CH_3)_4Si$ as the internal standard; coupling constants (J) are given in Hz. Low-resolution mass spectra were obtained using a Hewlett-Packard Model 1800A GCD GC-MS system (Palo Alto, CA, USA). Elemental analyses (± 0.4%) were determined by Atlantic Microlabs (Norcross, GA, USA) and are provided only for new compounds.

General procedure for domino reduction-heterocyclization.

To a solution of the 2-nitrobenzaldehyde (1.32 mmol, 1 equiv.) in AcOH (10 mL) under N_2 was added the active methylene compound: 3 equiv. for all active ketones except ethyl benzoylacetate, deoxybenzoin, 1-benzoylacetone, dimedone and methyl 4-phenylacetoacetate where 2 equiv. were used. The mixture was stirred for 15 min at 95-110 °C before addition of Fe (<100 mesh, 4 equiv. relative to the nitro compound). When TLC (20% EtOAc in hexane) indicated complete consumption of the starting material (3-6 h), unreacted Fe was removed by filtration through Celite before the solution was diluted with ether (50 mL) and washed with water (3 × 30 mL) to remove the AcOH. The ether was washed with $NaHCO_3$ (2 × 25 mL) and saturated NaCl (1 × 25 mL), and then dried (Na_2SO_4). Removal of the solvent under vacuum gave a crude product, which was further purified by column chromatography (25 cm × 2 cm) using increasing concentrations of ethyl acetate (5-15%) in hexanes to afford analytical samples of the heterocyclic products. The compounds prepared are given in Tables 1-5.

3.3.1. ¹H NMR for Ethyl 2-methylquinoline-3-carboxylate (Table 1, entry 1)

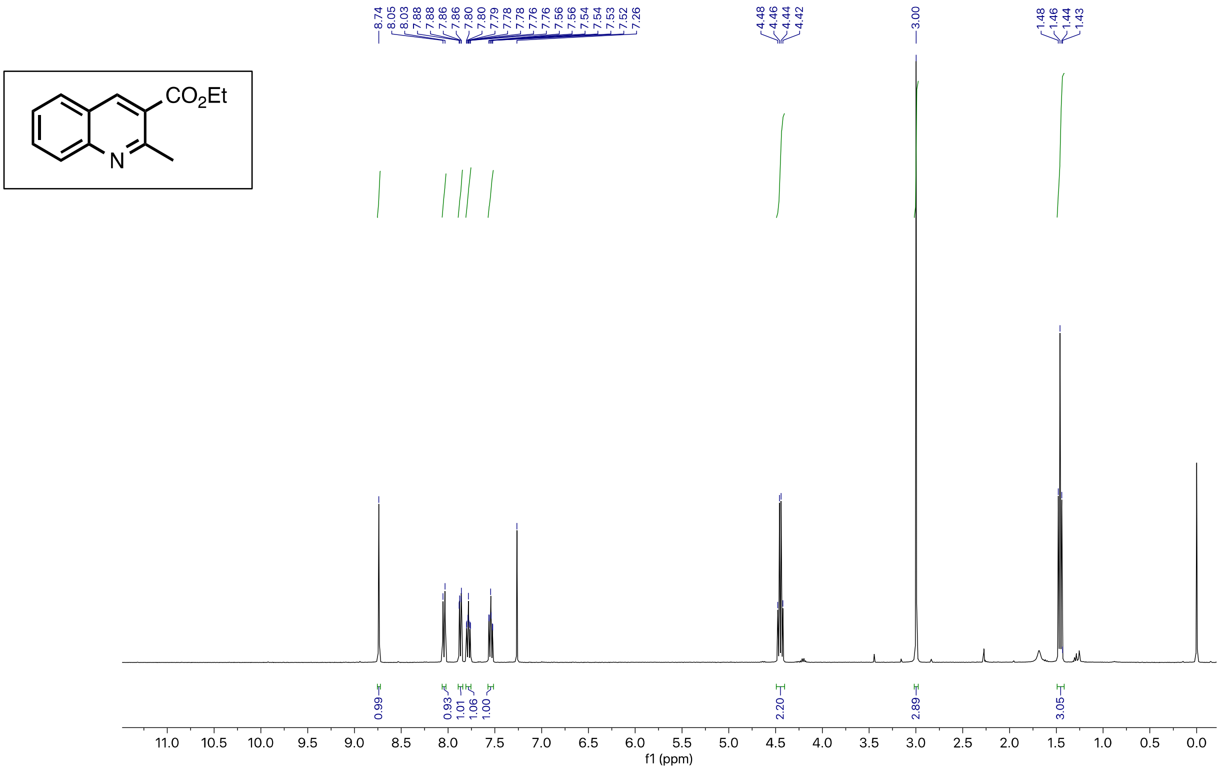

3.3.1. ¹³C NMR for Ethyl 2-methylquinoline-3-carboxylate (Table 1, entry 1)

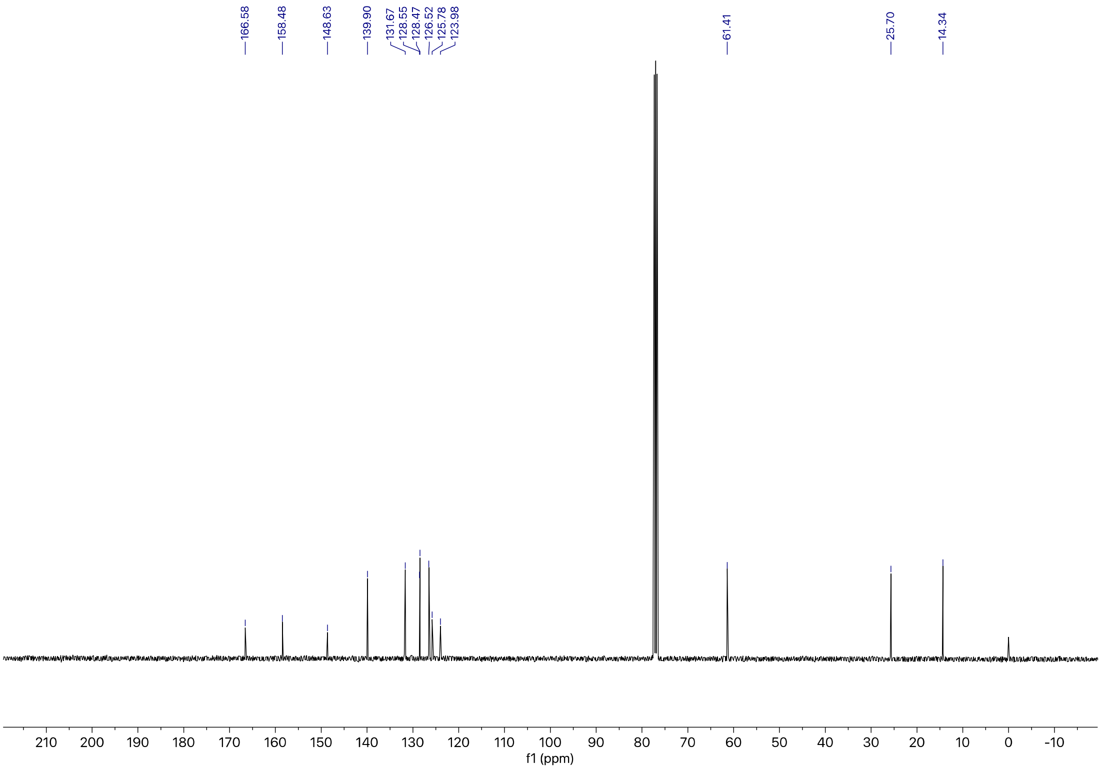

3.3.2. ¹H NMR for Ethyl 2-(trifluoromethyl)quinoline-3-carboxylate (Table 1, entry 2)

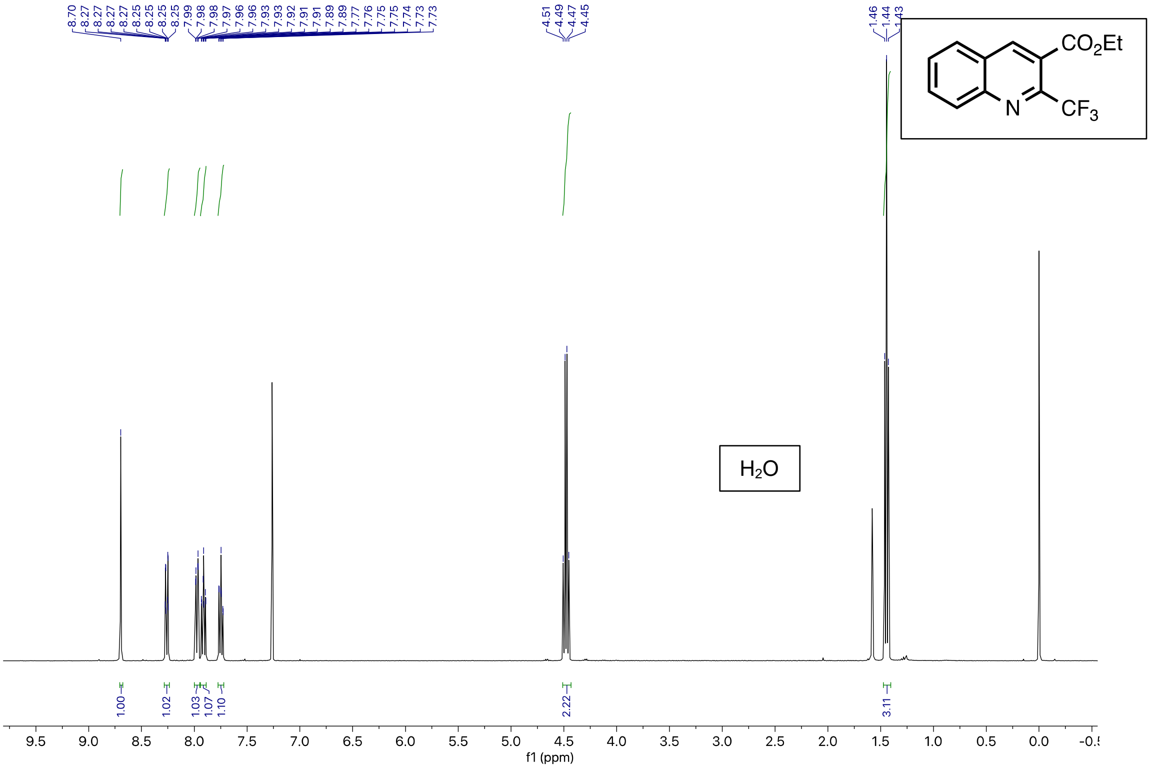

3.3.2. ^{13}C NMR for Ethyl 2-(trifluoromethyl)quinoline-3-carboxylate (Table 1, entry 2)

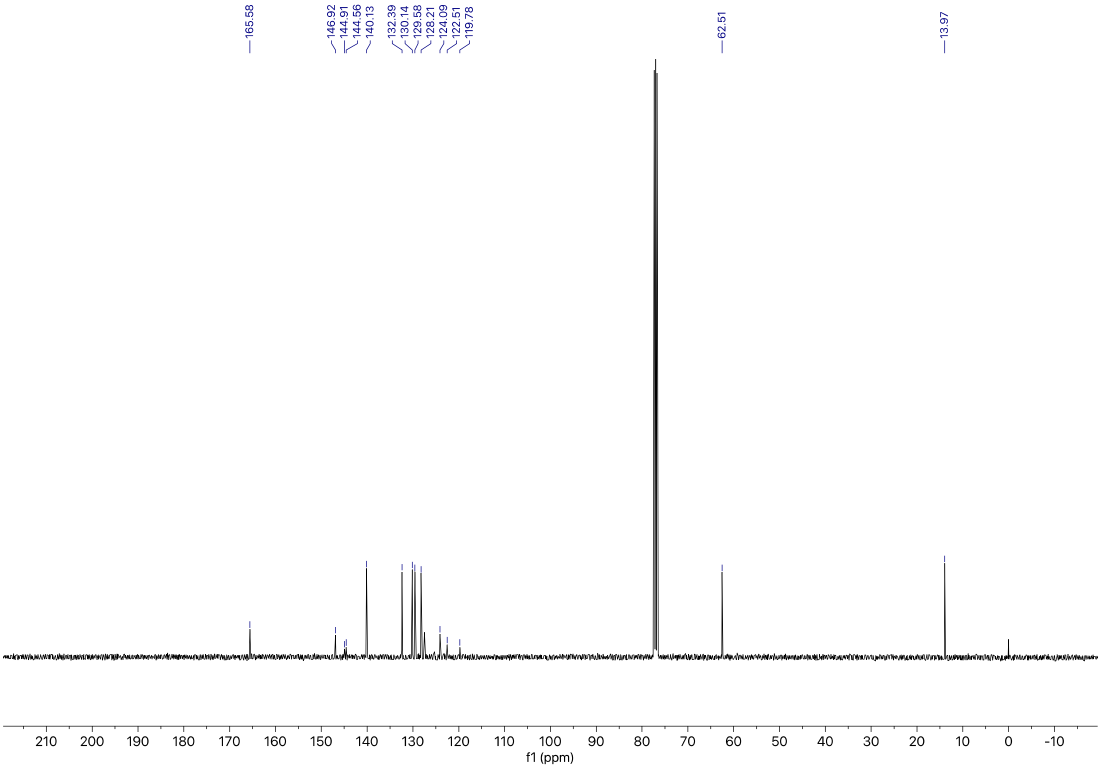

3.3.3. ^{1}H NMR for Methyl 2-ethylquinoline-3-carboxylate (Table 1, entry 3)

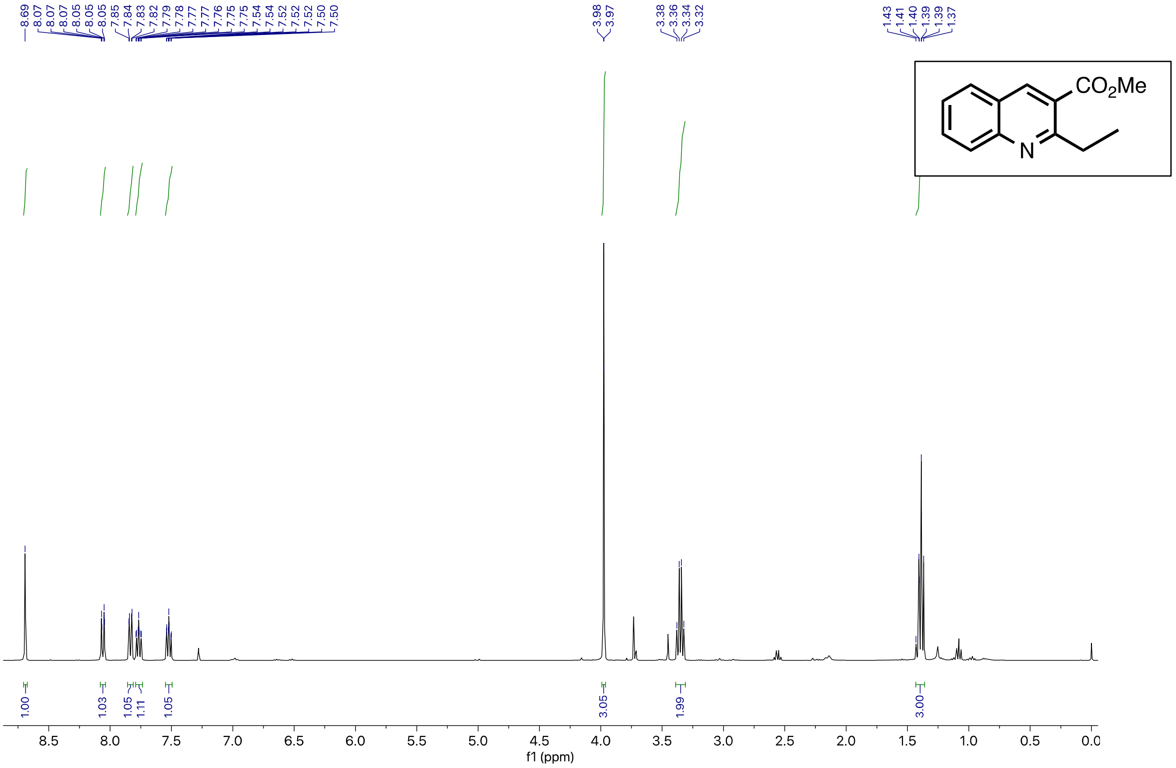

3.3.3. ^{13}C NMR for Methyl 2-ethylquinoline-3-carboxylate (Table 1, entry 3)

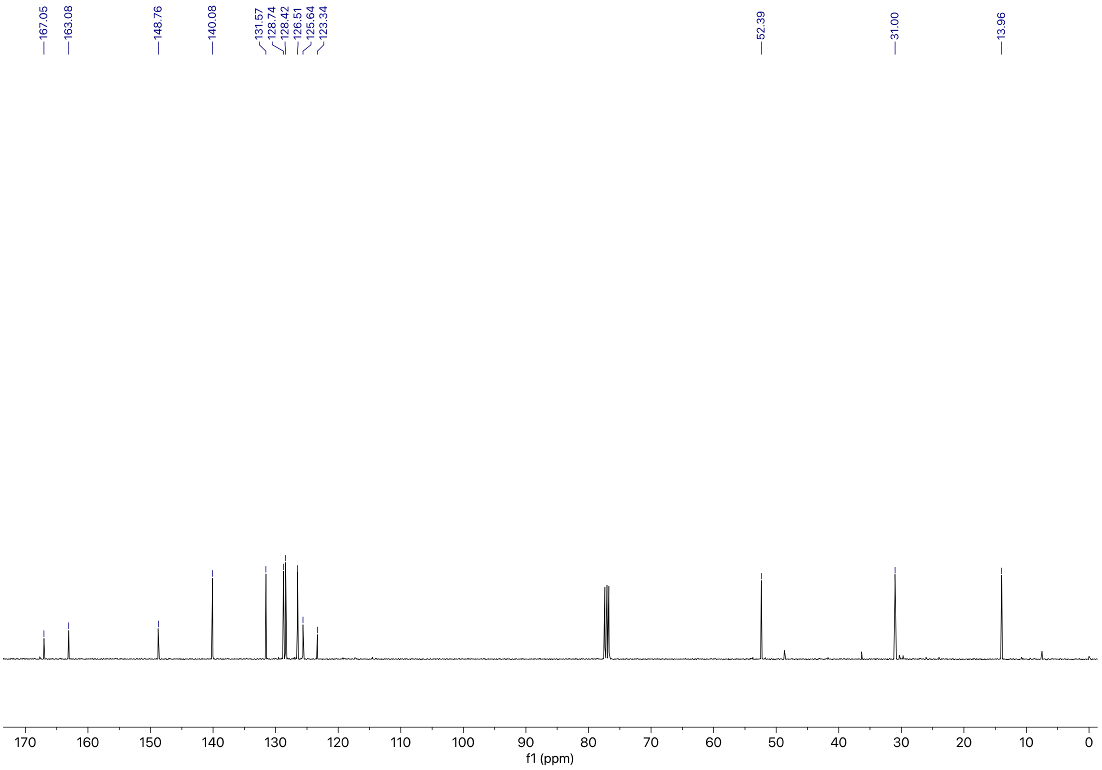

3.3.4. ^{1}H NMR for Methyl 2-pentylquinoline-3-carboxylate (Table 1, entry 4)

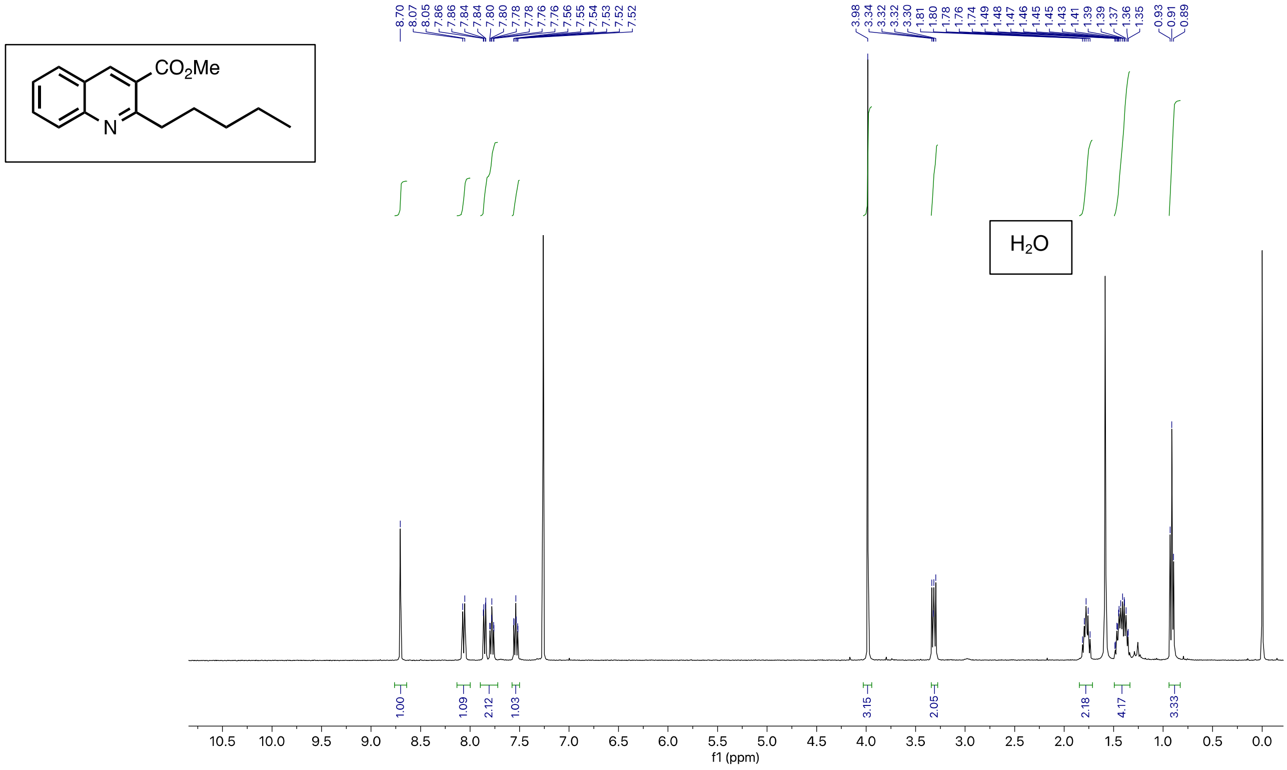

3.3.4. ^{13}C NMR for Methyl 2-pentylquinoline-3-carboxylate (Table 1, entry 4)

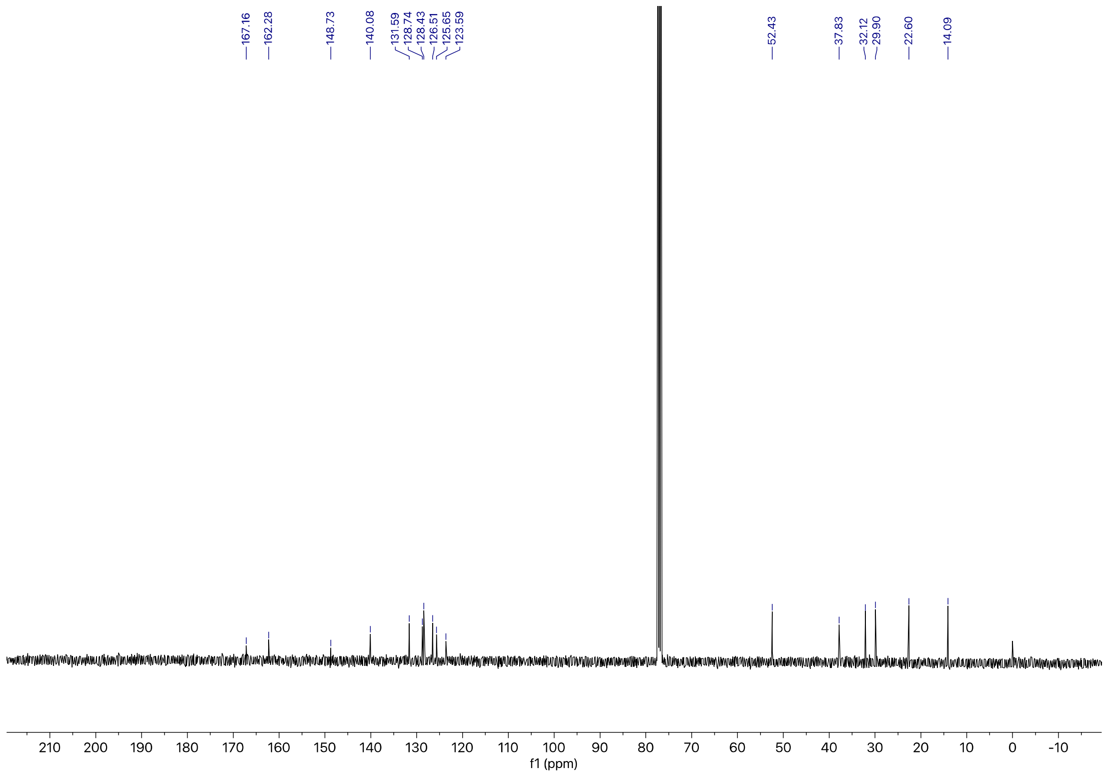

3.3.5. ^{1}H NMR for Methyl 2-isopropylquinoline-3-carboxylate (Table 1, entry 5)

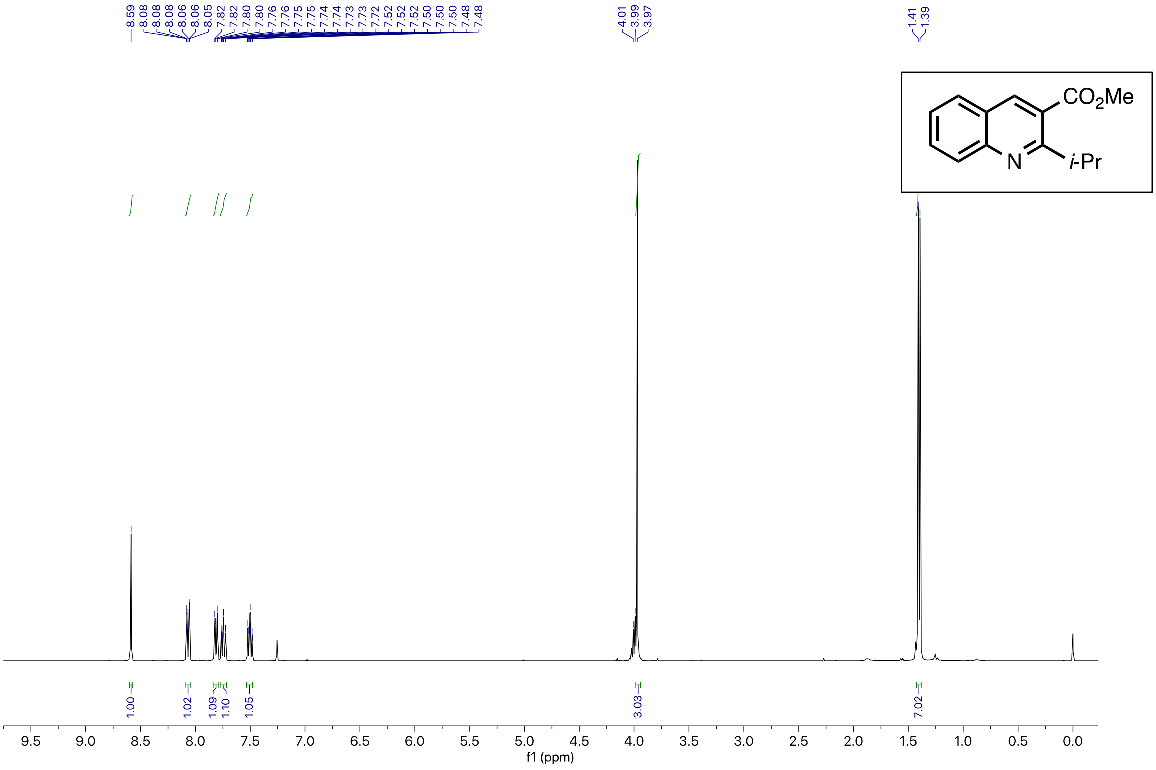

3.3.5. ^{13}C NMR for Methyl 2-isopropylquinoline-3-carboxylate (Table 1, entry 5)

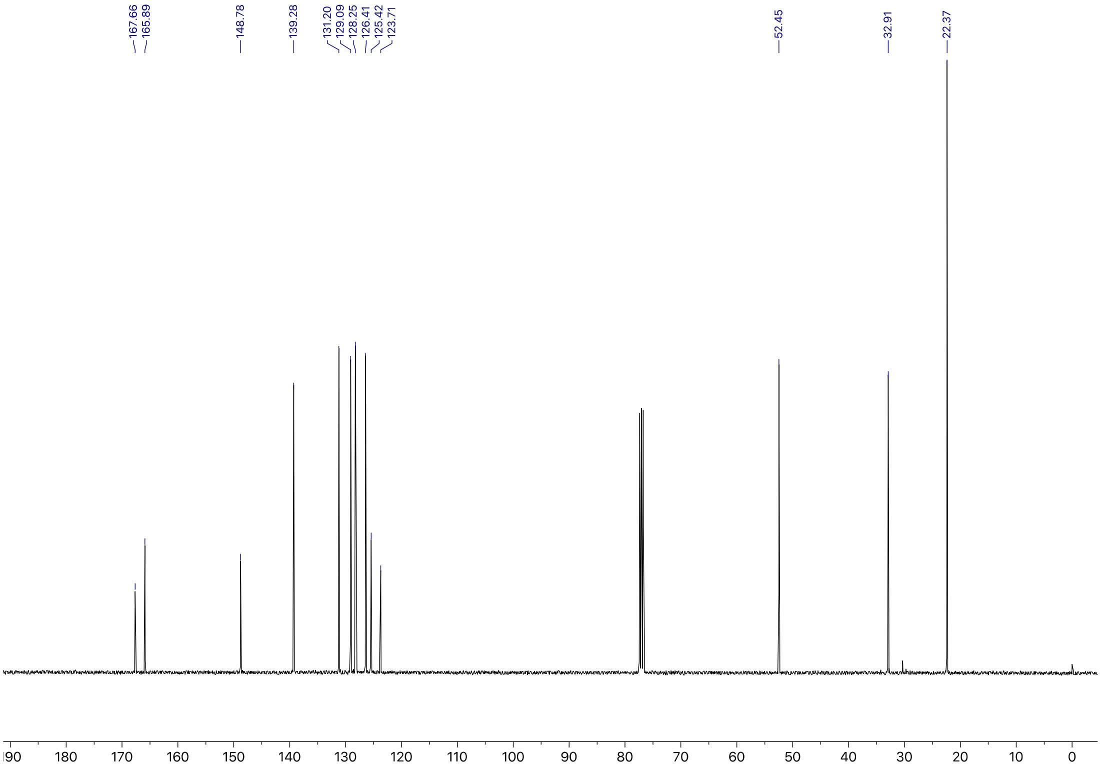

3.3.6. ^{1}H NMR for Methyl 2-(*tert*-butyl) quinoline-3-carboxylate (Table 1, entry 6)

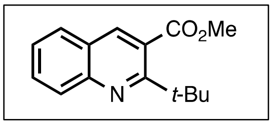

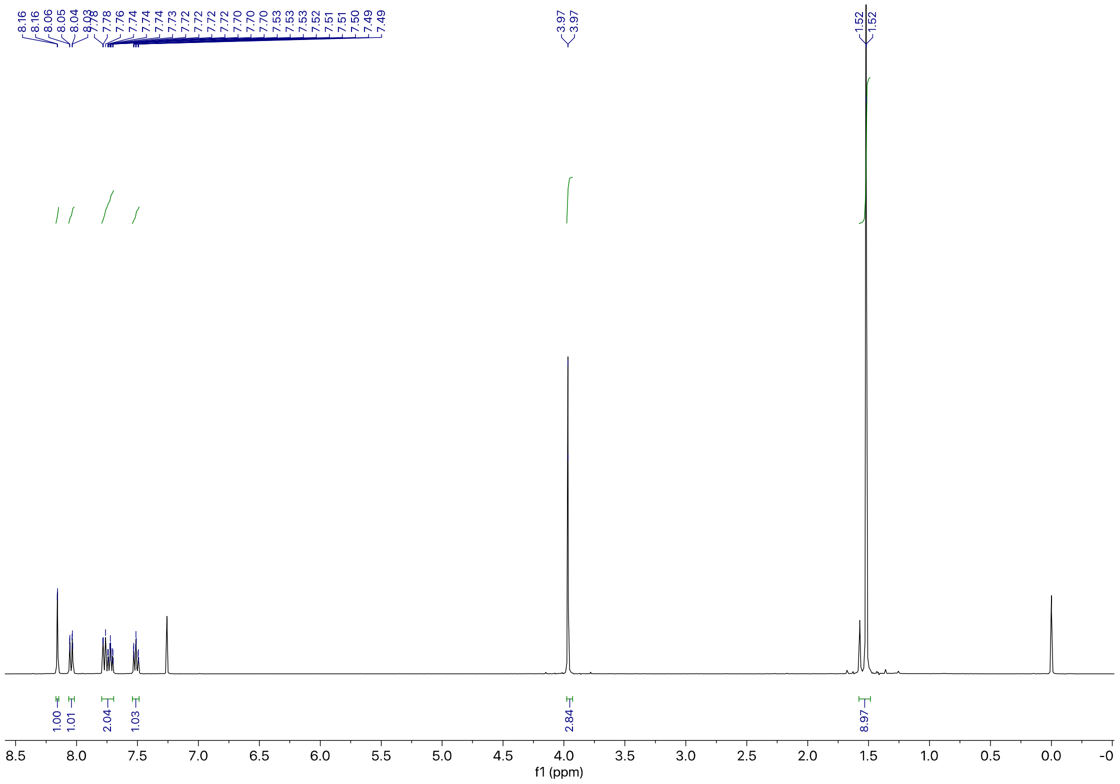

3.3.6. ^{13}C NMR for Methyl 2-(*tert*-butyl) quinoline-3-carboxylate (Table 1, entry 6)

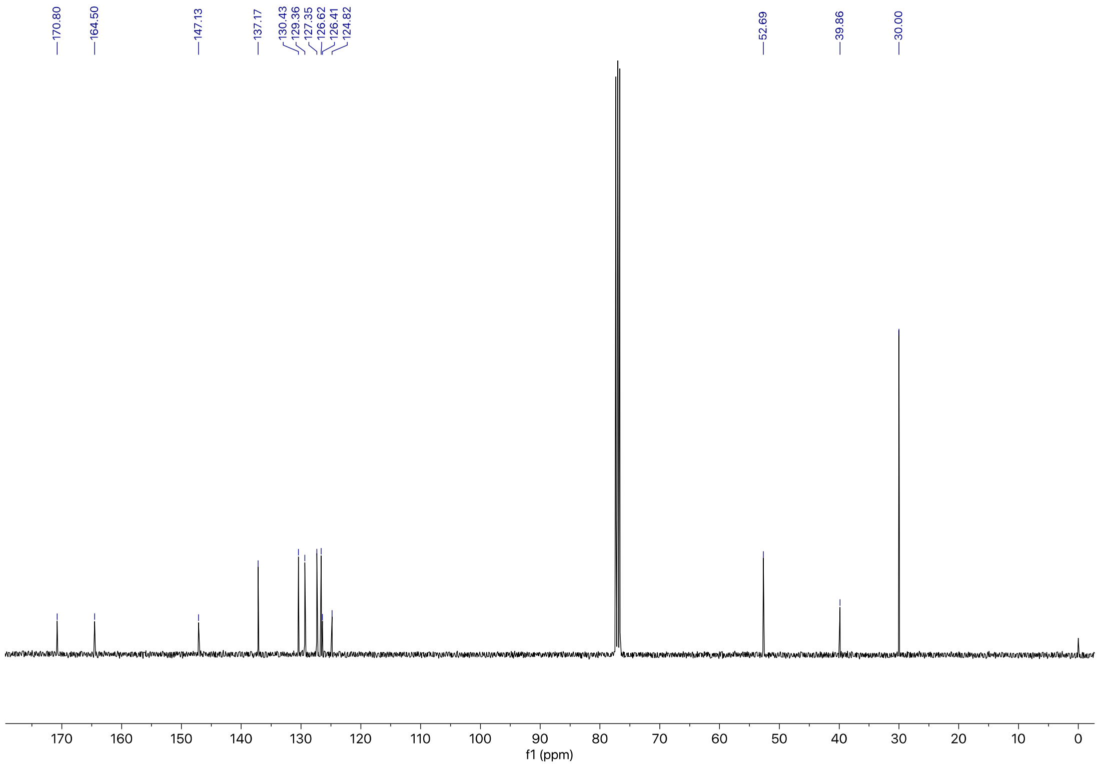

3.3.7. ^{1}H NMR for Ethyl 2-phenylquinoline-3-carboxylate (Table 1, entry 7)

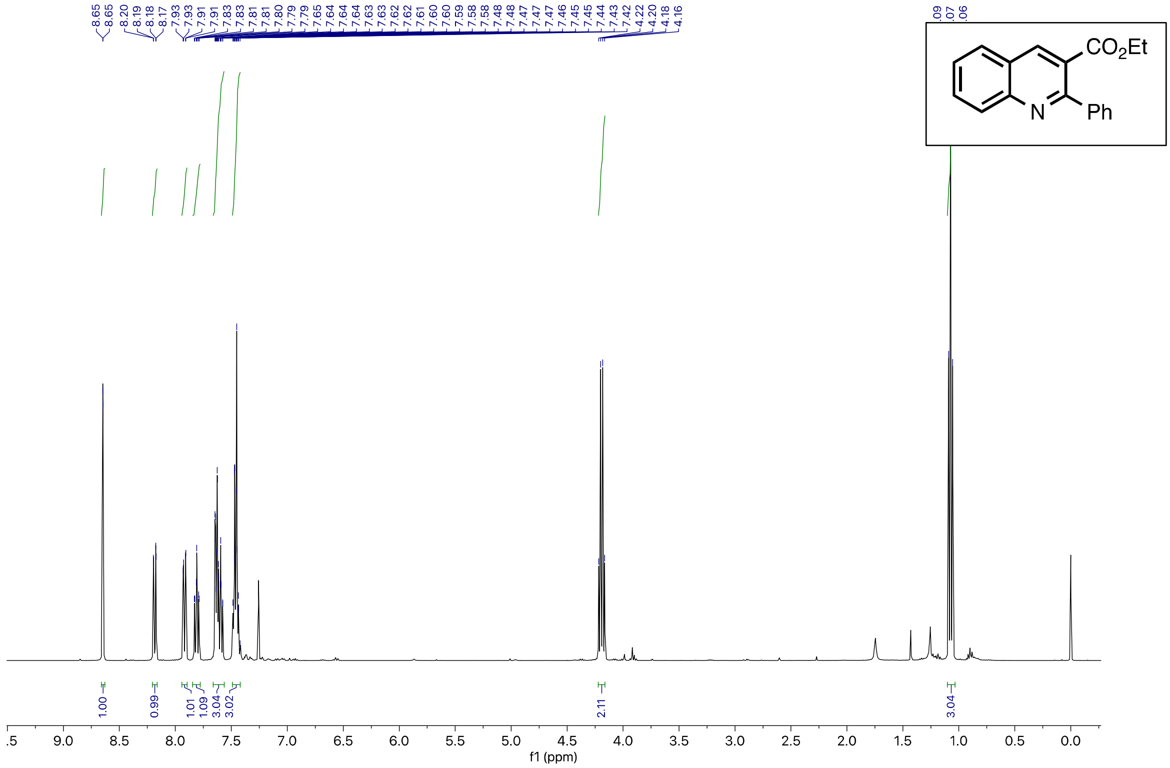

3.3.7. ^{13}C NMR for Ethyl 2-phenylquinoline-3-carboxylate (Table 1, entry 7)

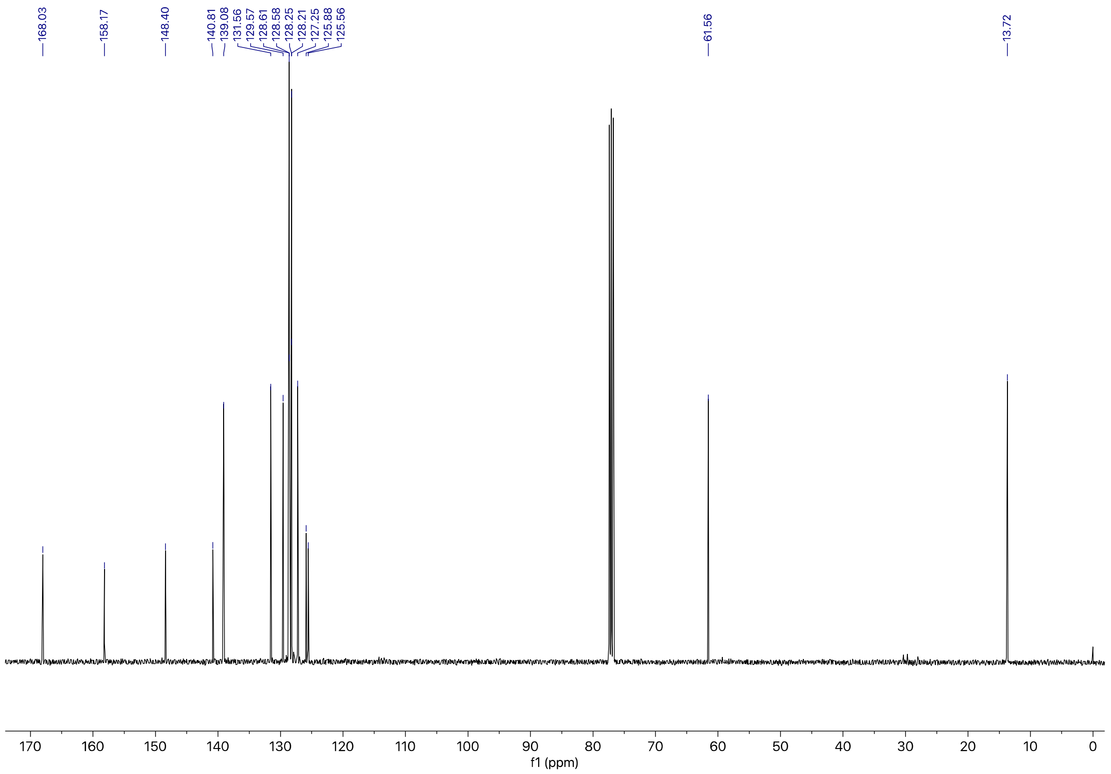

3.3.8. ^{1}H NMR for Methyl 2-benzylquinoline-3-carboxylate (Table 1, entry 8)

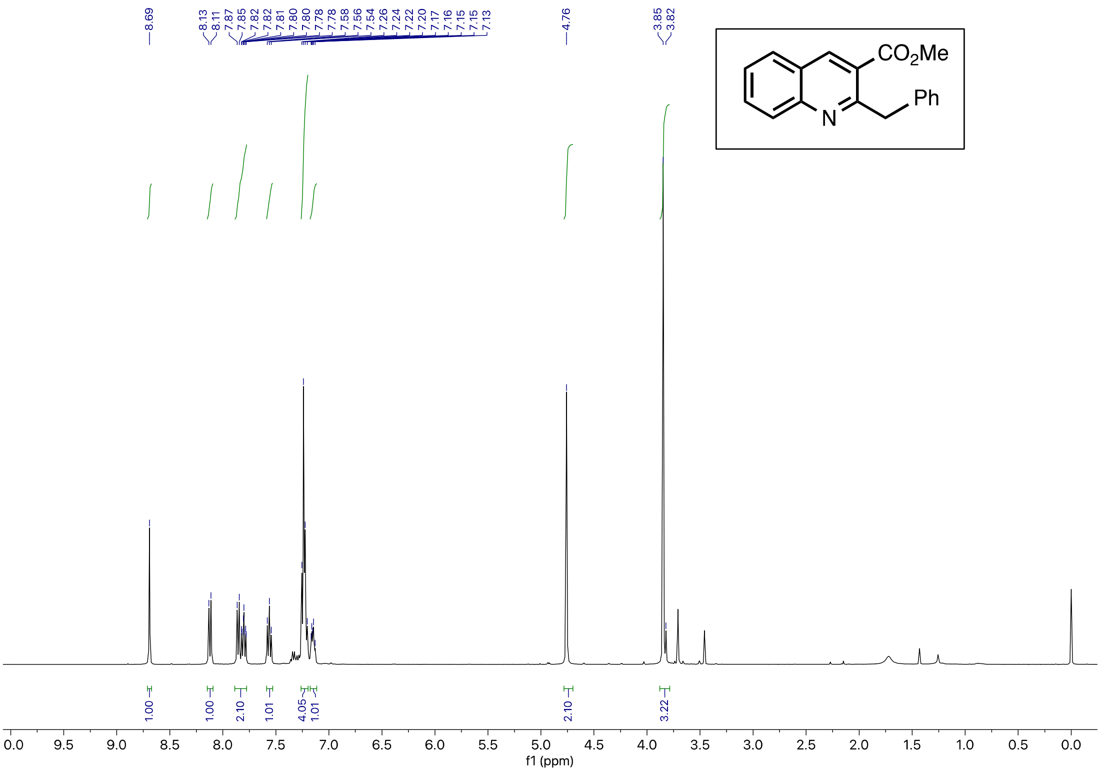

3.3.8. ^{13}C NMR for Methyl 2-benzylquinoline-3-carboxylate (Table 1, entry 8)

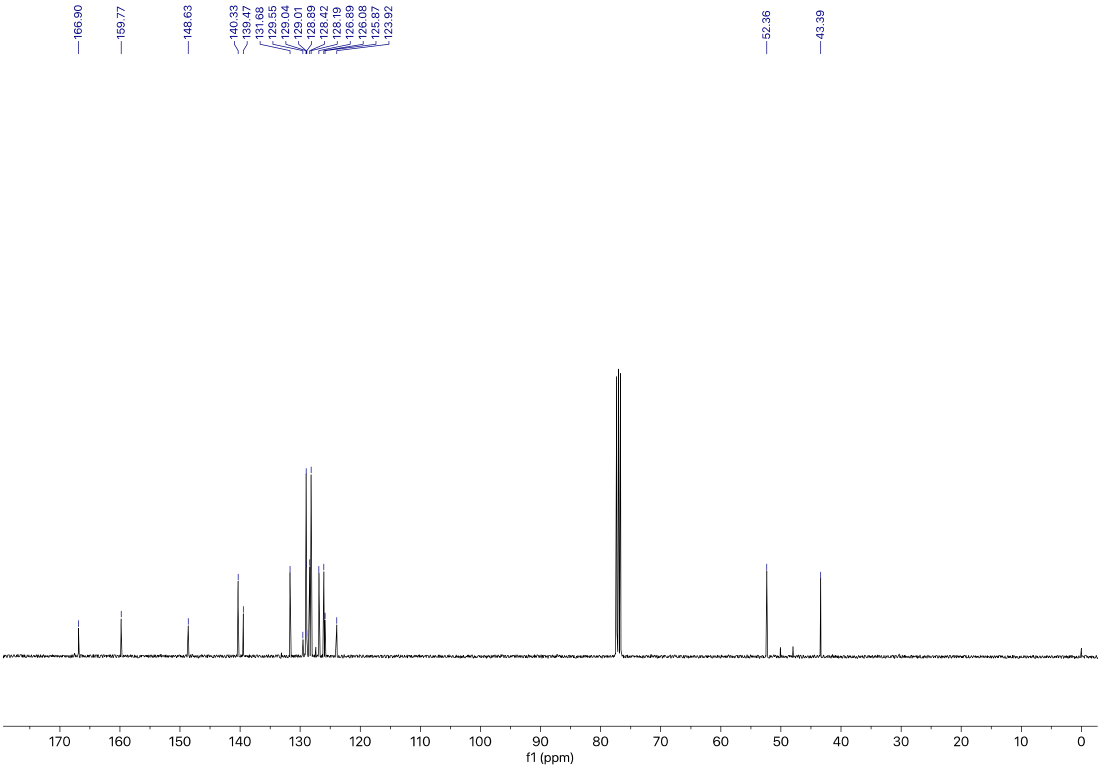

3.3.9. ^{1}H NMR for Methyl 2-phenethylquinoline-3-carboxylate (Table 1, entry 9)

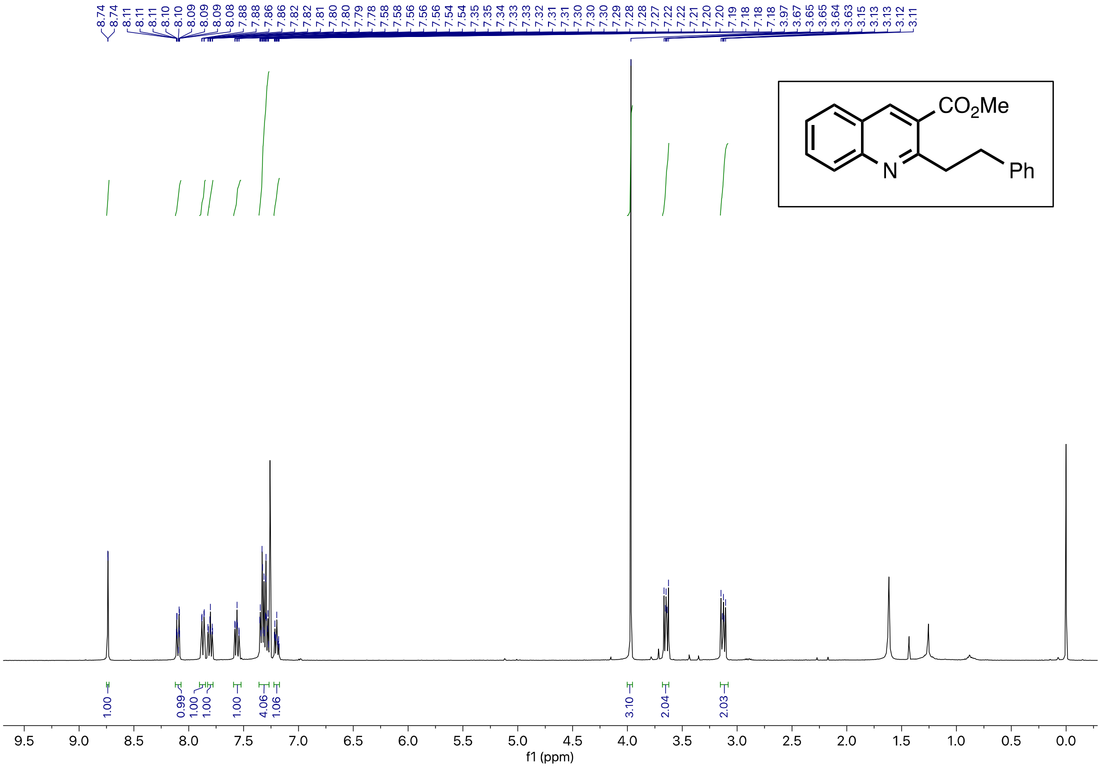

3.3.9. ^{13}C NMR for Methyl 2-phenethylquinoline-3-carboxylate (Table 1, entry 9)

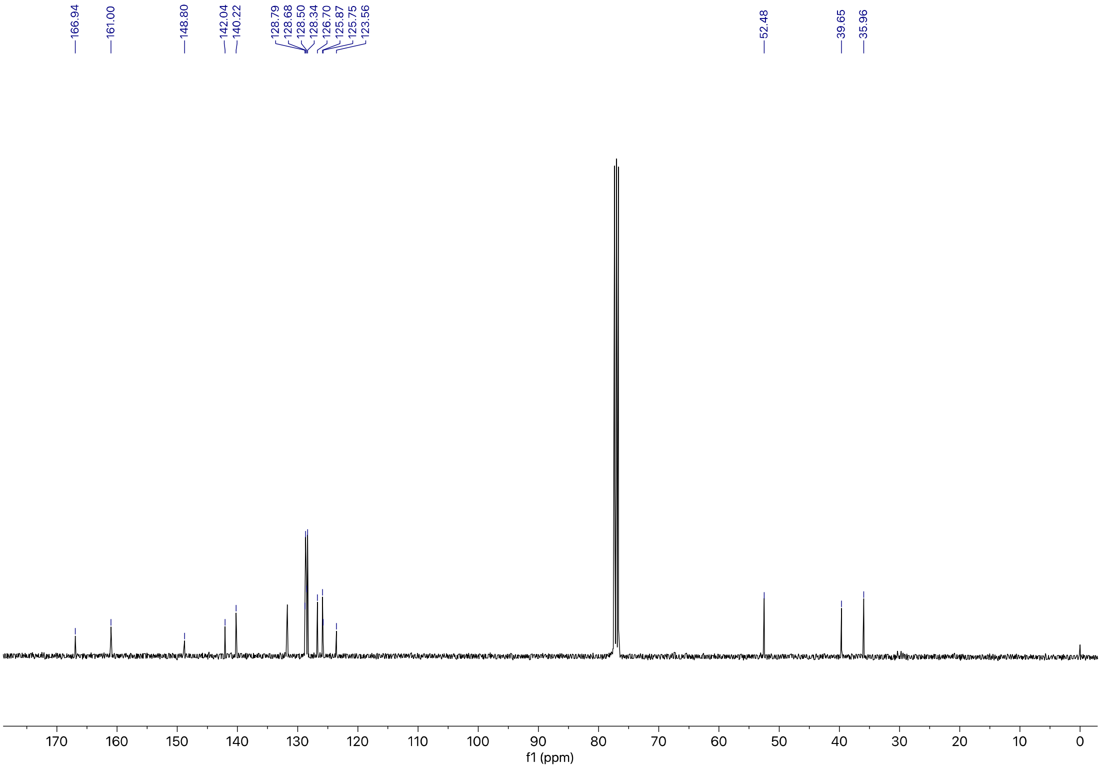

3.3.10. ^{1}H NMR for Methyl 2-(phenoxymethyl)quinoline-3-carboxylate (Table 1, entry 10)

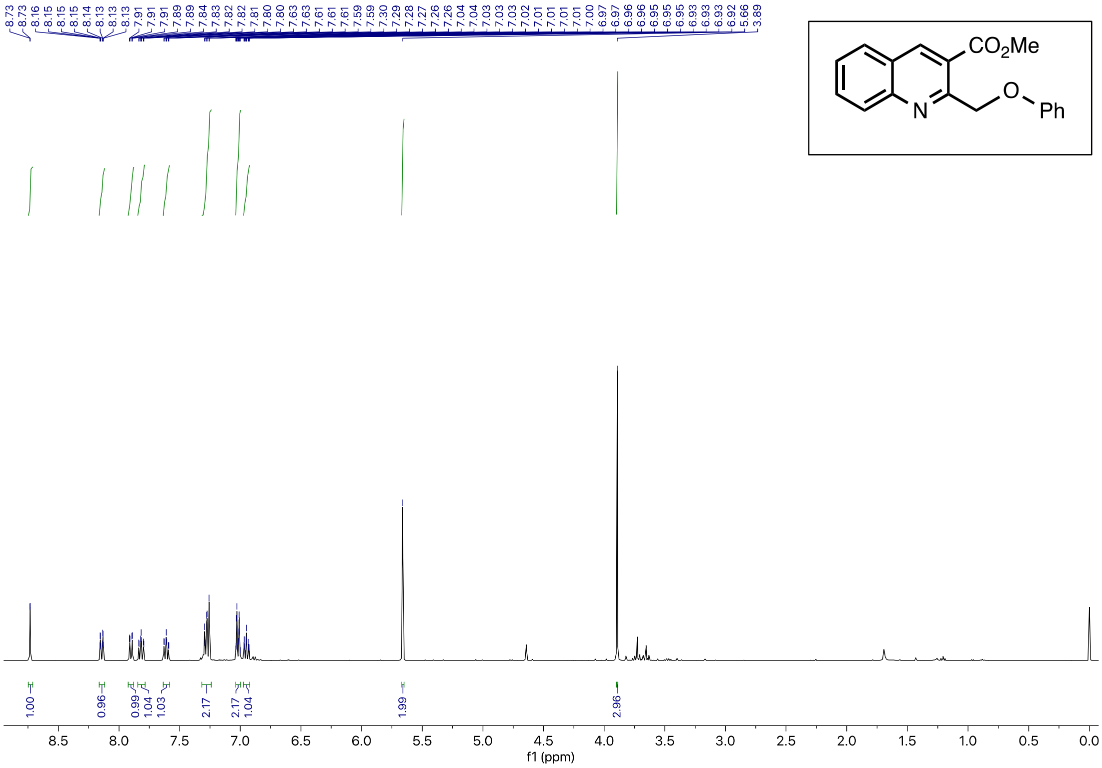

3.3.10. ^{13}C NMR for Methyl 2-(phenoxymethyl)quinoline-3-carboxylate (Table 1, entry 10)

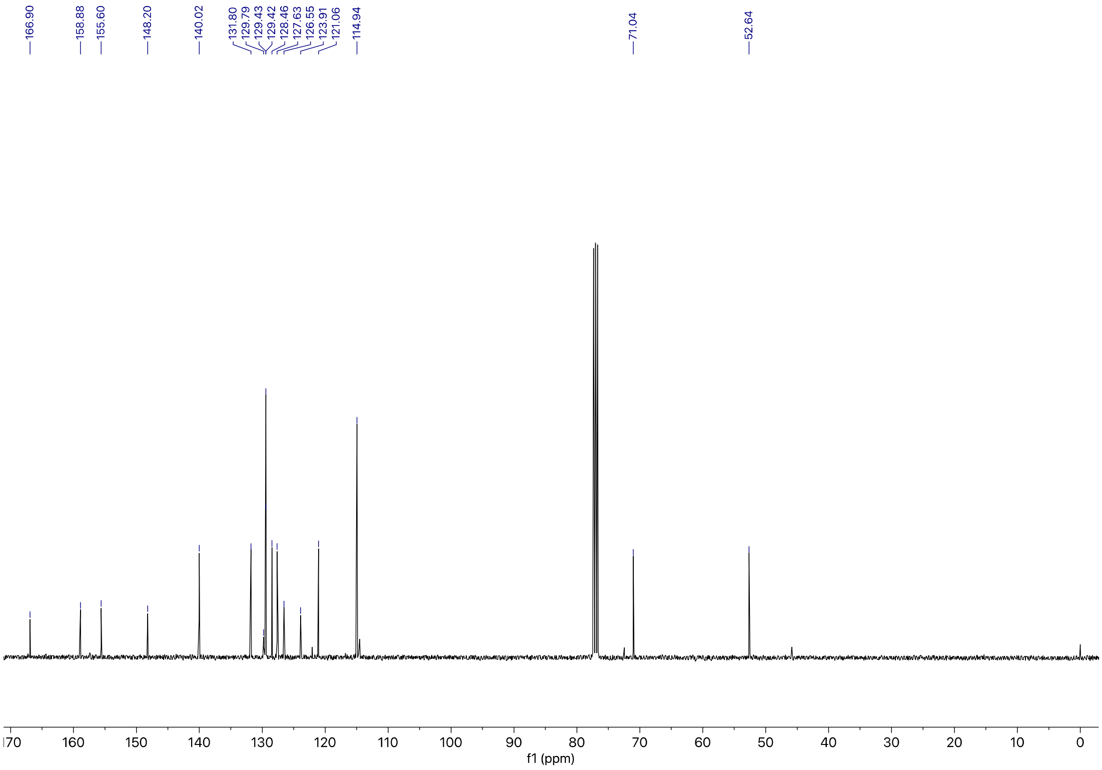

3.3.11. ^{1}H NMR for 2-Methyl-3-(phenylsulfonyl)quinoline (Table 1, entry 11)

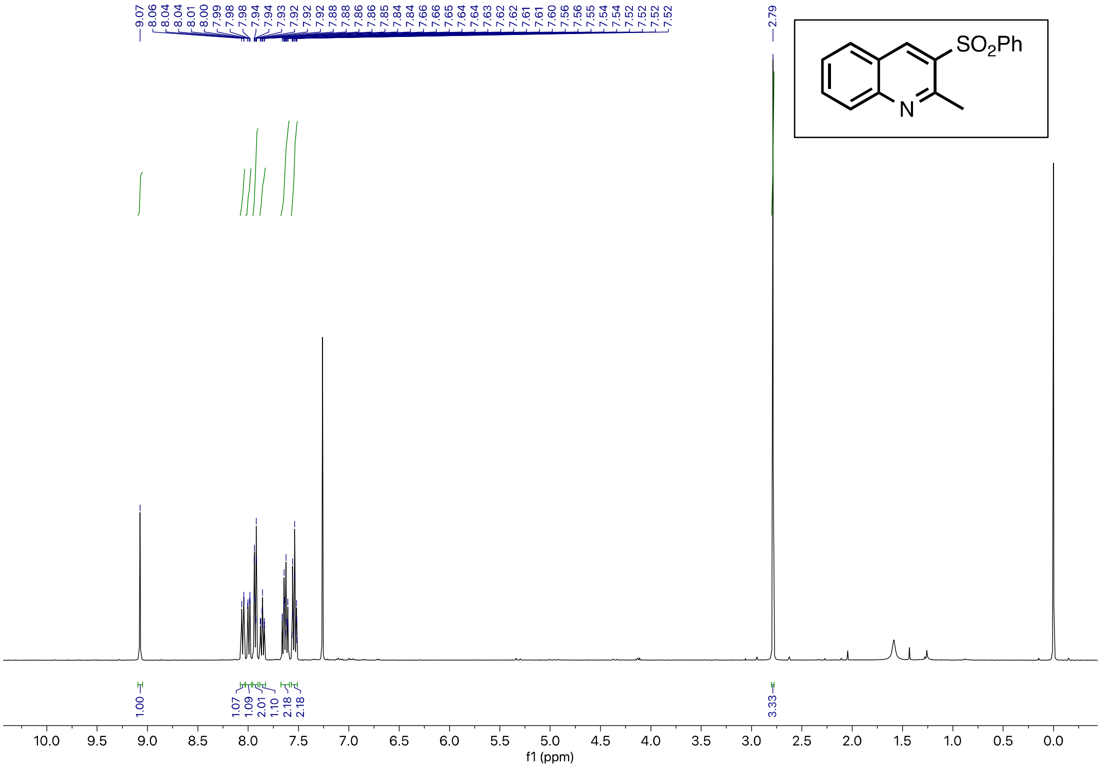

3.3.11. ^{13}C NMR for 2-Methyl-3-(phenylsulfonyl)quinoline (Table 1, entry 11)

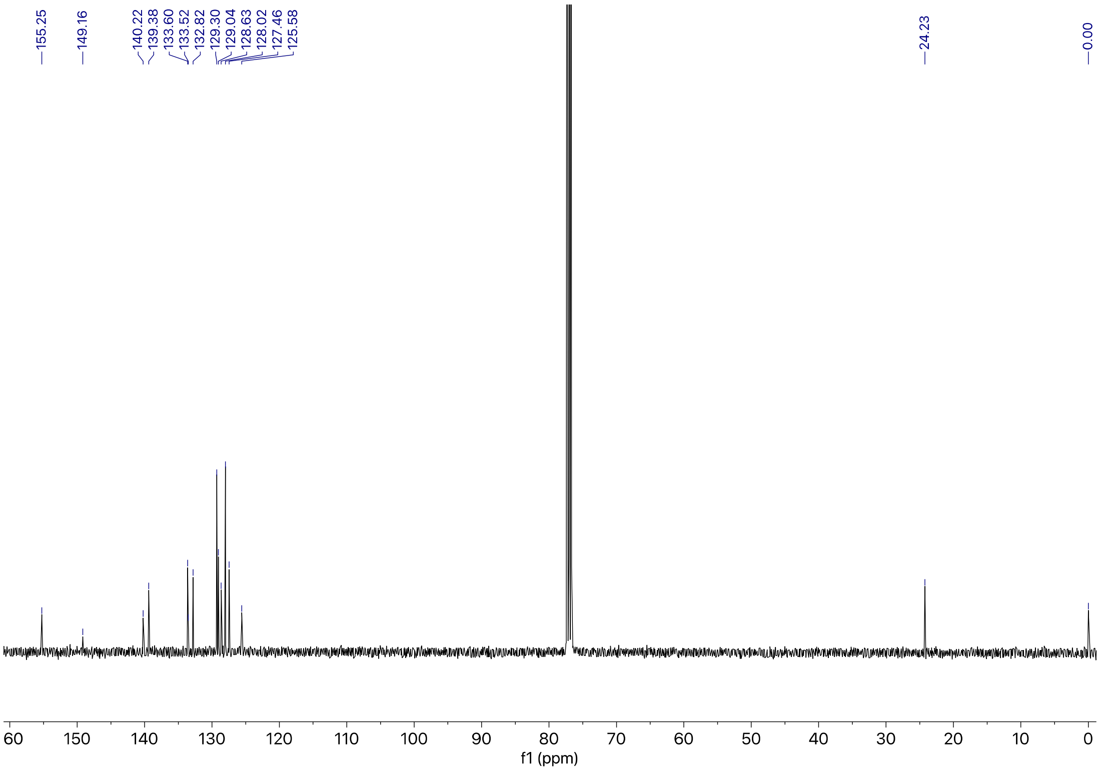

3.3.12. ^{1}H NMR for 2-Methylquinoline-3-carbonitrile (Table 1, entry 12)

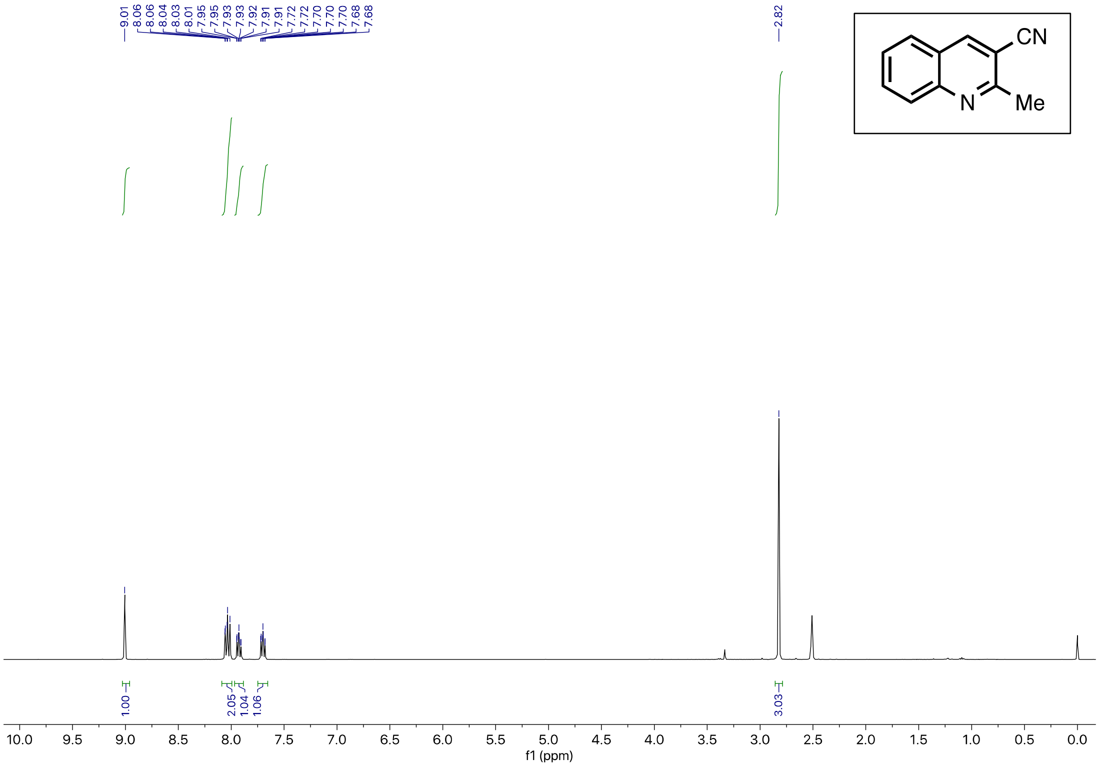

3.3.12. ^{13}C NMR for 2-Methylquinoline-3-carbonitrile (Table 1, entry 12)

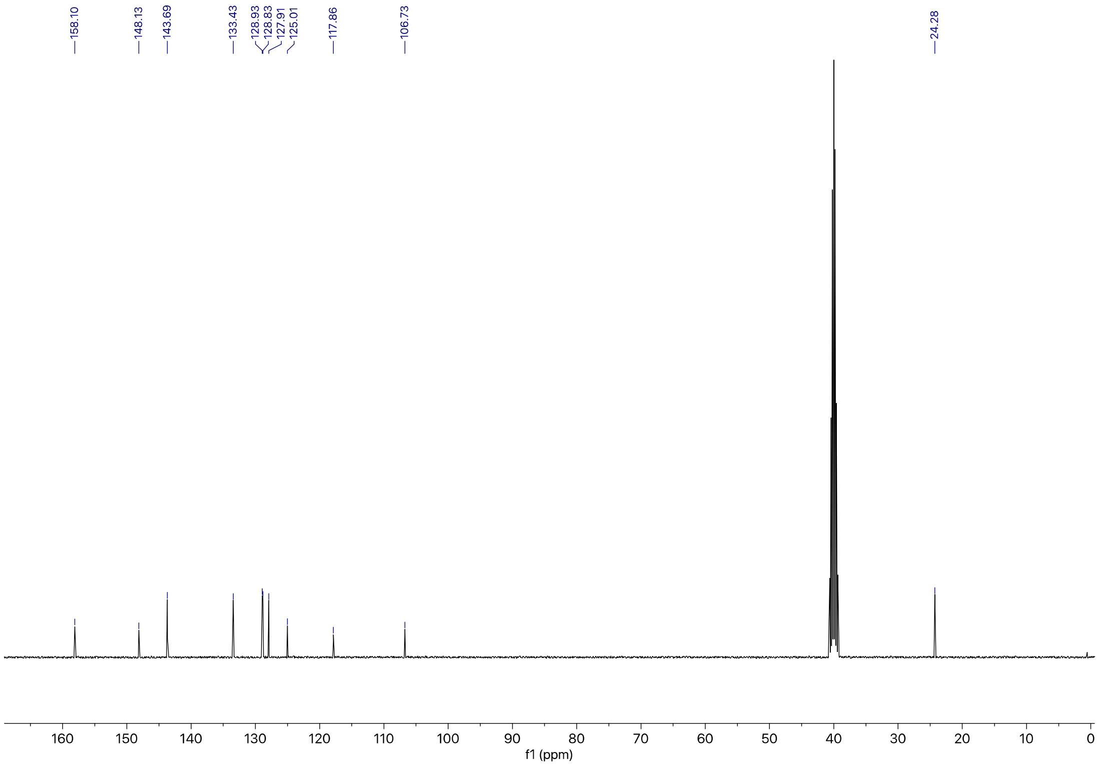

3.3.13. ^{1}H NMR for 2-Phenylquinoline-3-carbonitrile (Table 1, entry 13)

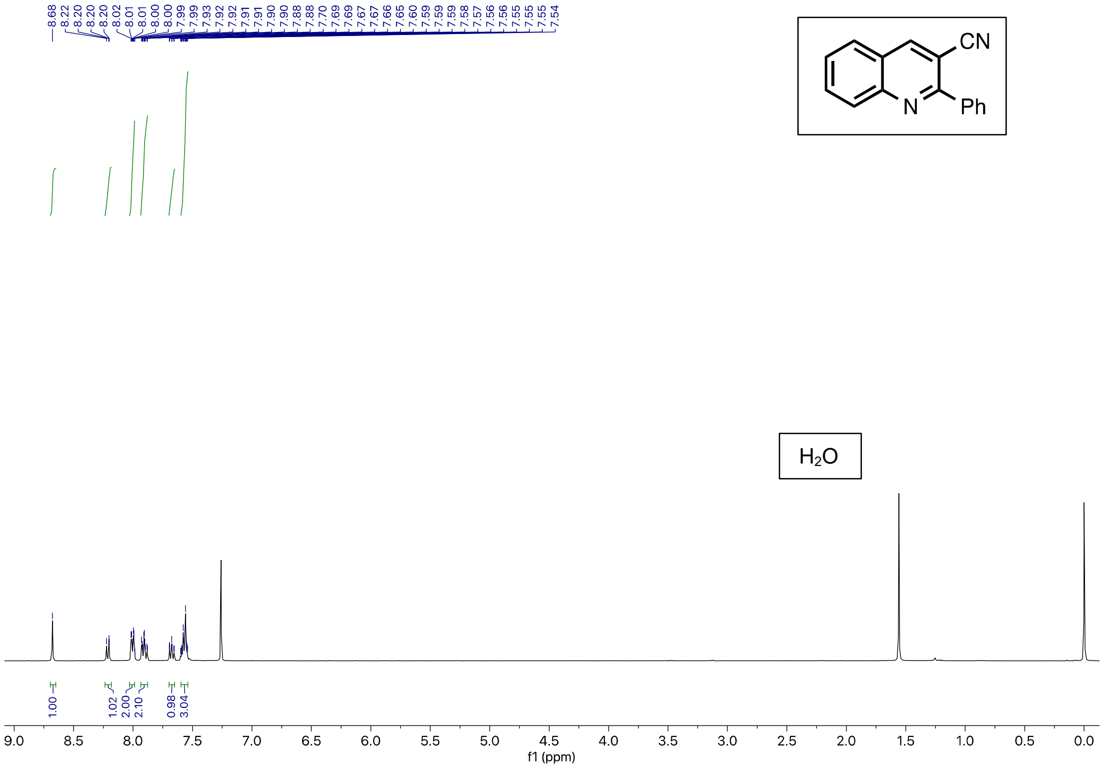

3.3.13. ^{13}C NMR for 2-Phenylquinoline-3-carbonitrile (Table 1, entry 13)

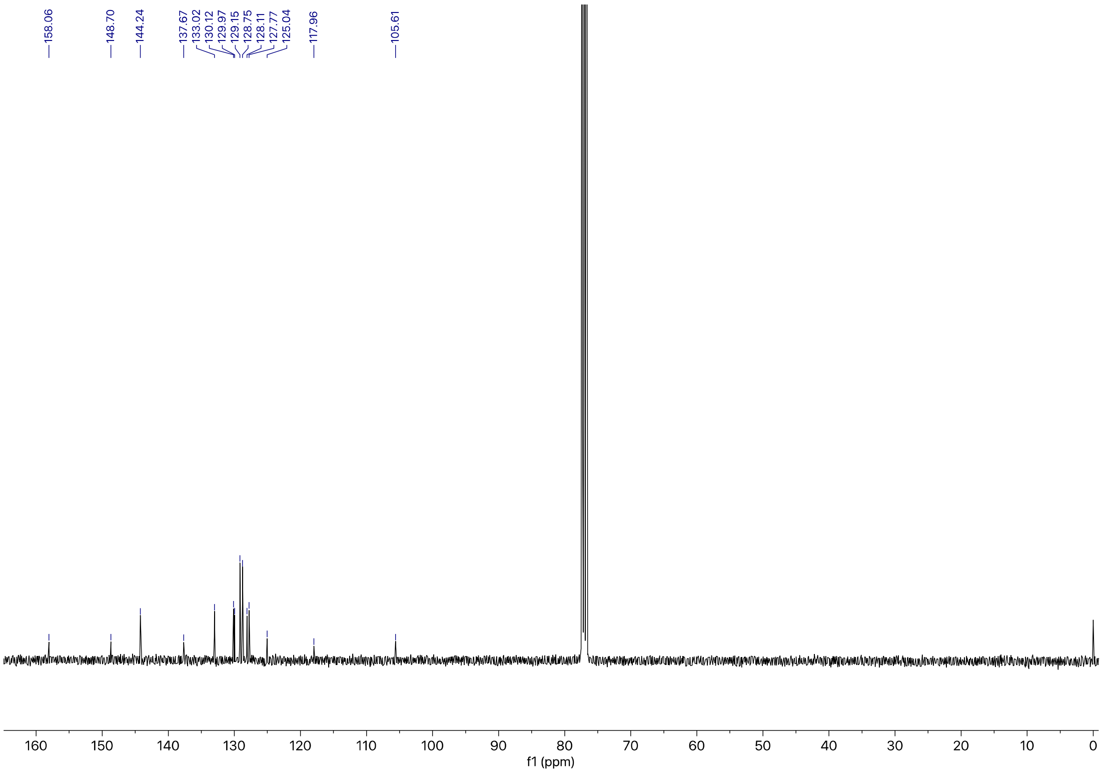

3.3.14. ^{1}H NMR for 3,3-Dimethyl-3,4-dihydroacridin-1(2*H*)-one (Table 1, entry 14)

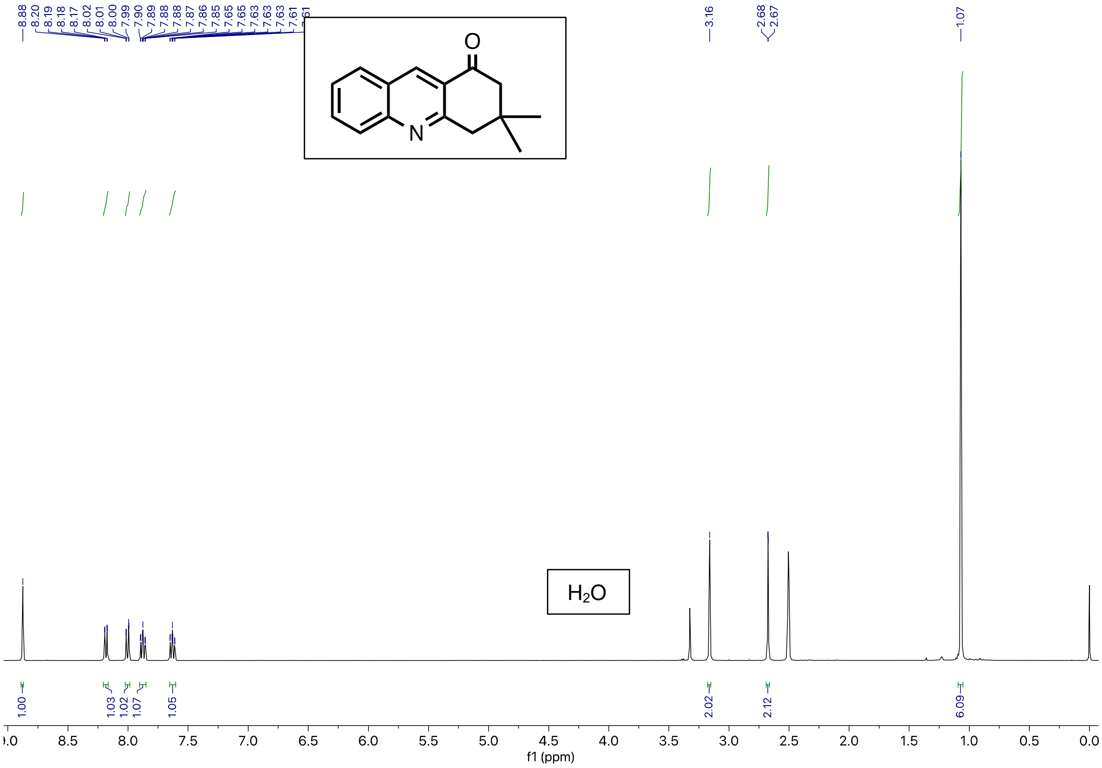

3.3.14. ^{13}C NMR for 3,3-Dimethyl-3,4-dihydroacridin-1(2*H*)-one (Table 1, entry 14)

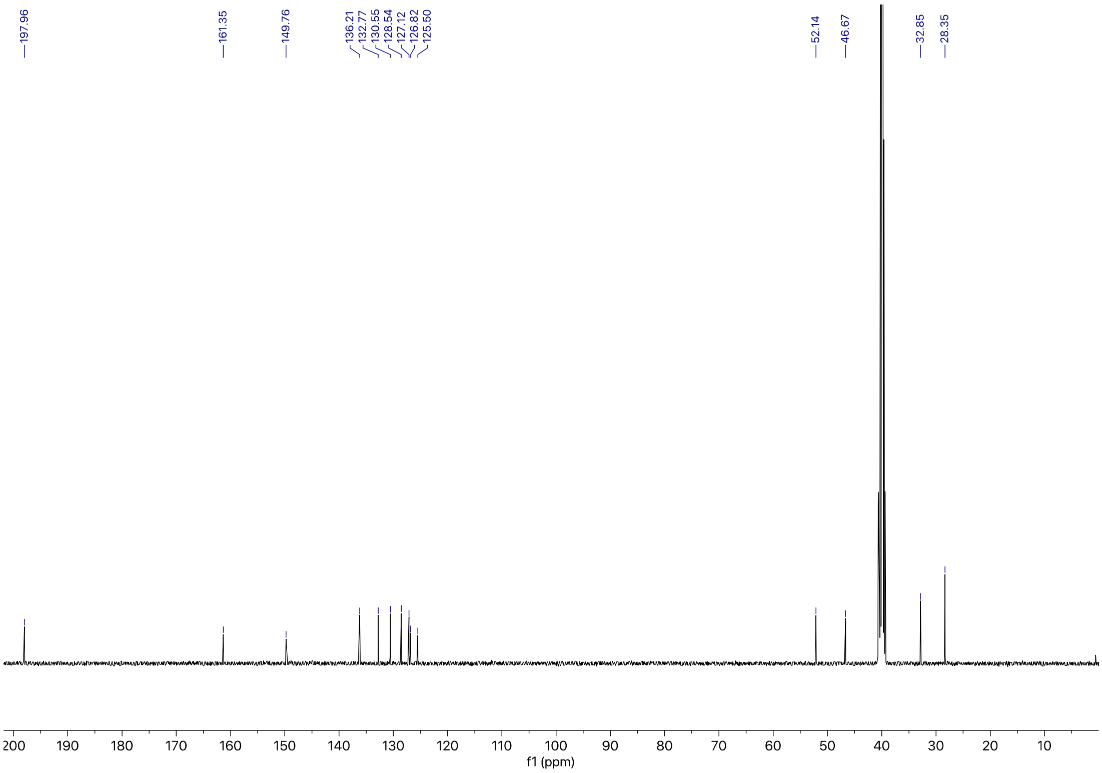

3.3.15. ^{1}H NMR for 1-(2-Methylquinolin-3-yl)ethan-1-one (Table 1, entry 15)

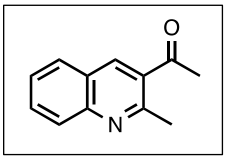

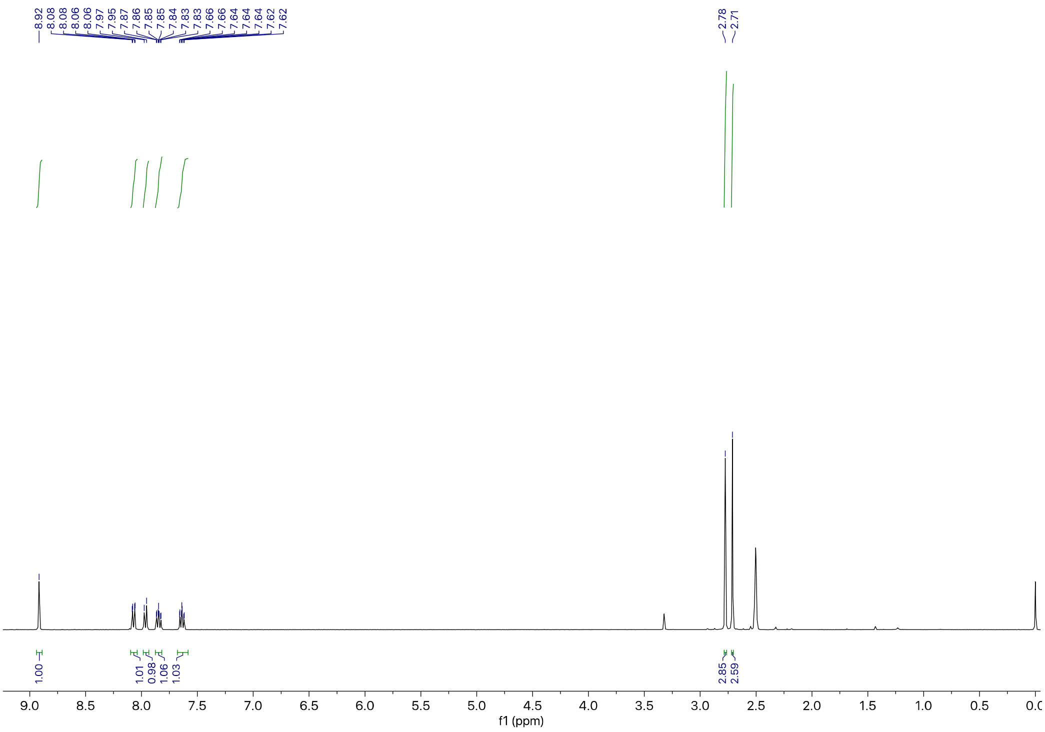

3.3.15. ^{13}C NMR for 1-(2-Methylquinolin-3-yl)ethan-1-one (Table 1, entry 15)

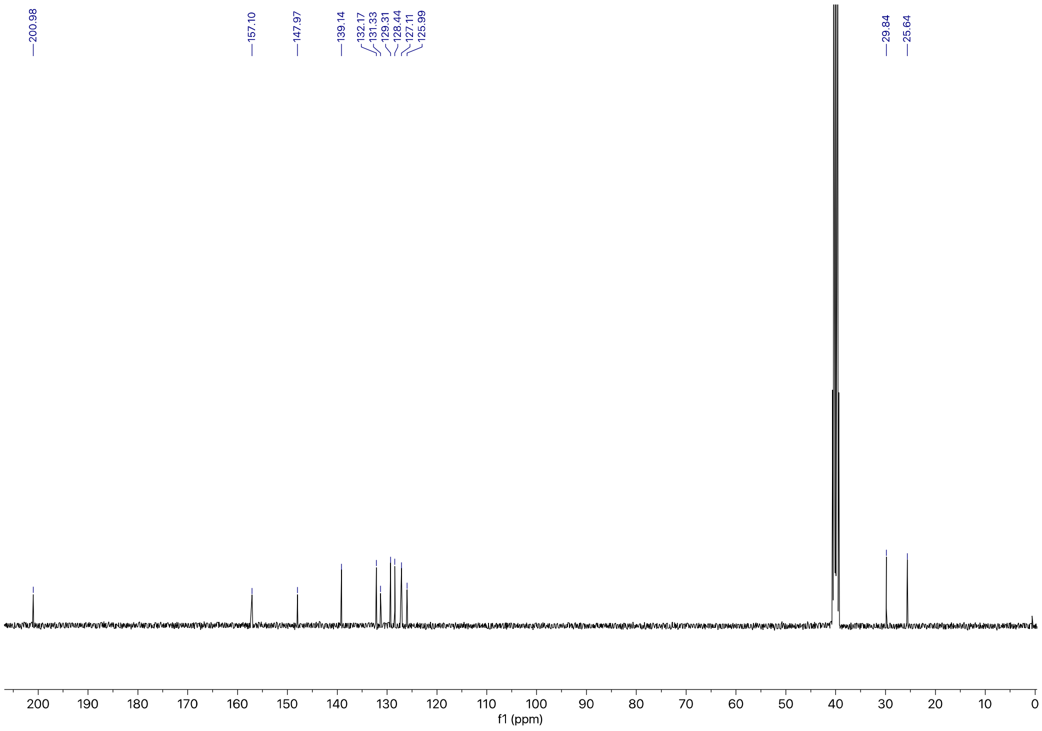

3.3.16. ^{1}H NMR for (2-Methylquinolin-3-yl)(phenyl)methanone (Table 1, entry 16)

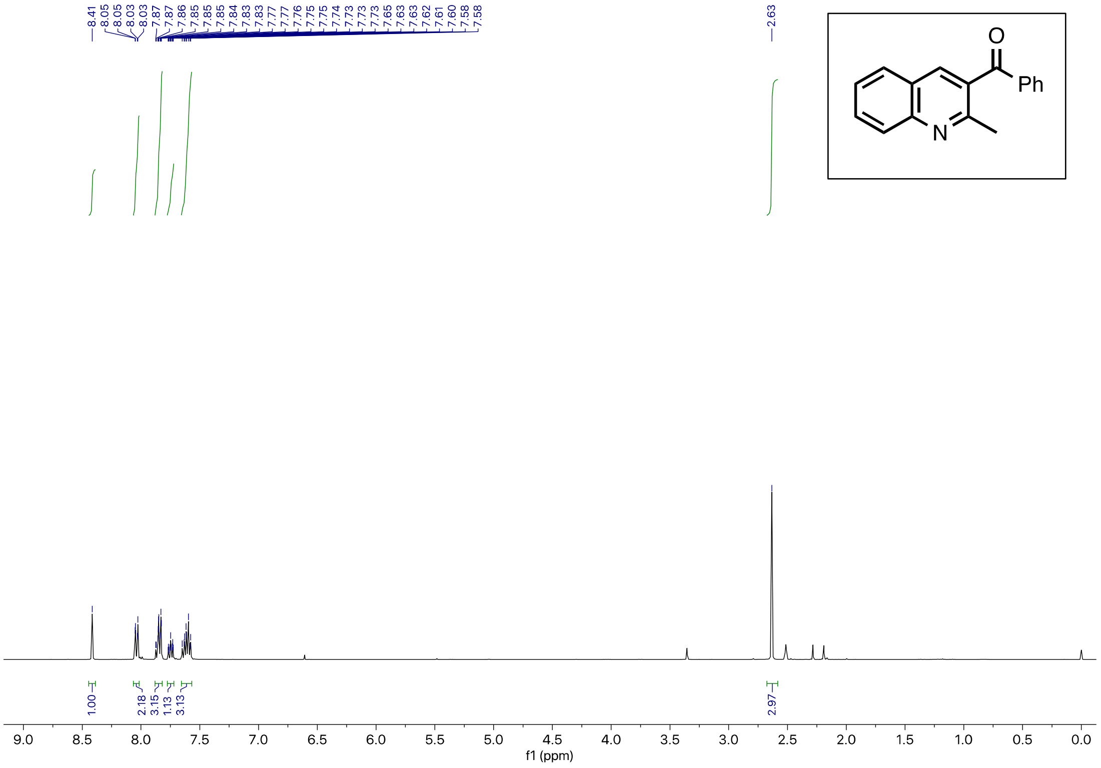

3.3.16. ^{13}C NMR for (2-Methylquinolin-3-yl)(phenyl)methanone (Table 1, entry 16)

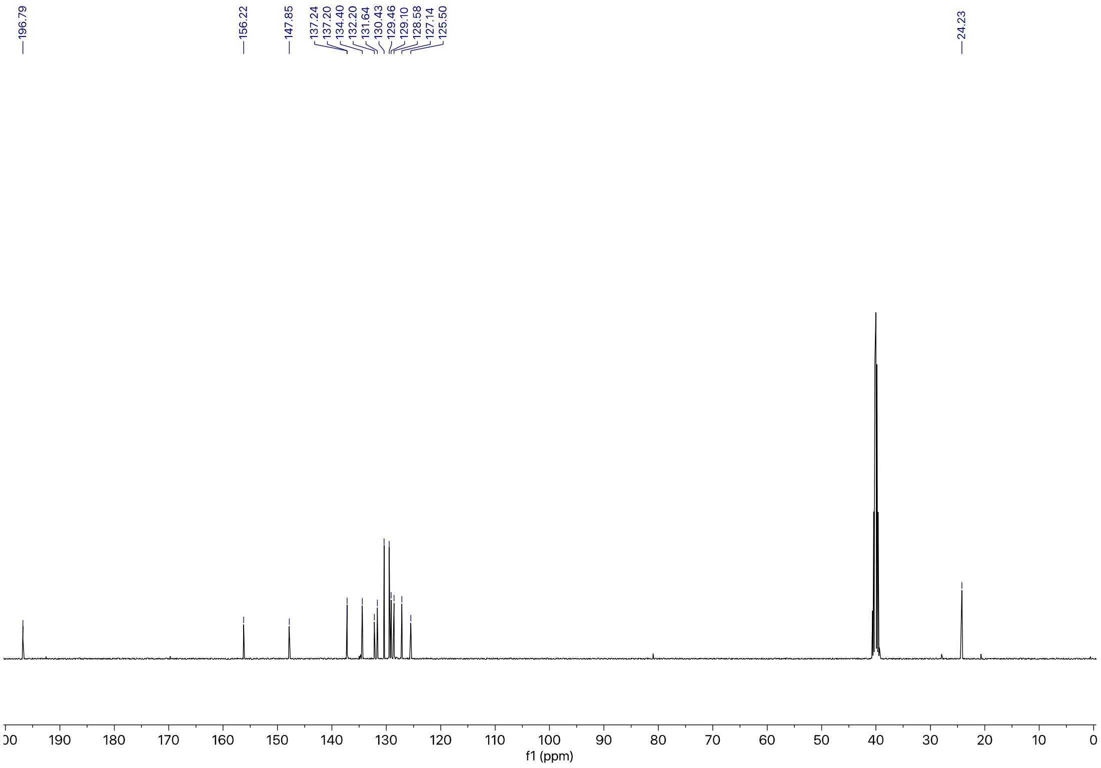

3.3.17. ^{1}H NMR for 2-Methyl-3-phenylquinoline (Table 1, entry 17)

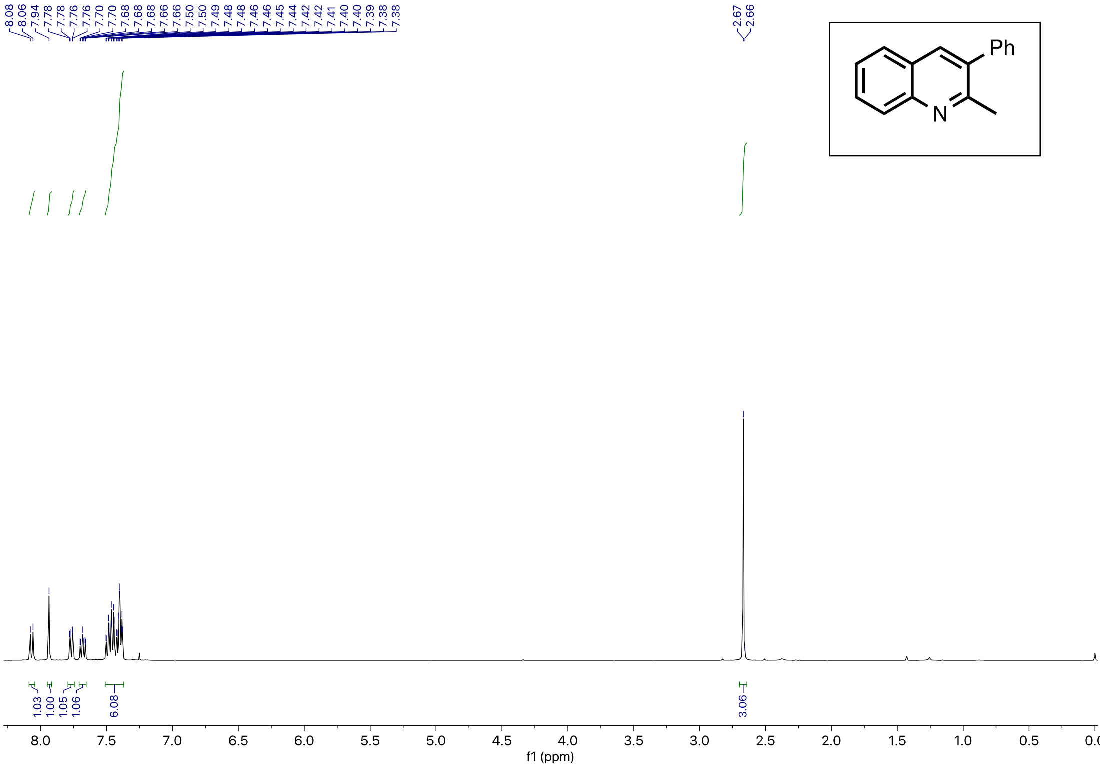

3.3.17. ^{13}C NMR for 2-Methyl-3-phenylquinoline (Table 1, entry 17)

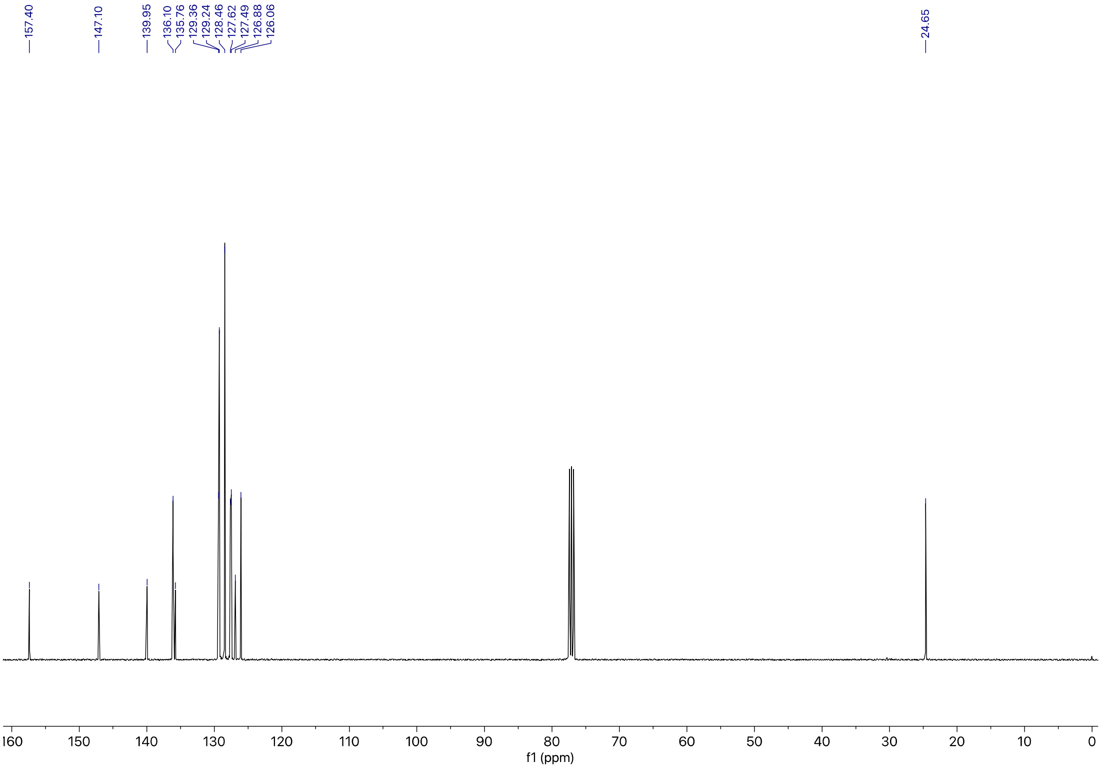

3.3.18. ^{1}H NMR for 2,3-Diphenylquinoline (Table 1, entry 18)

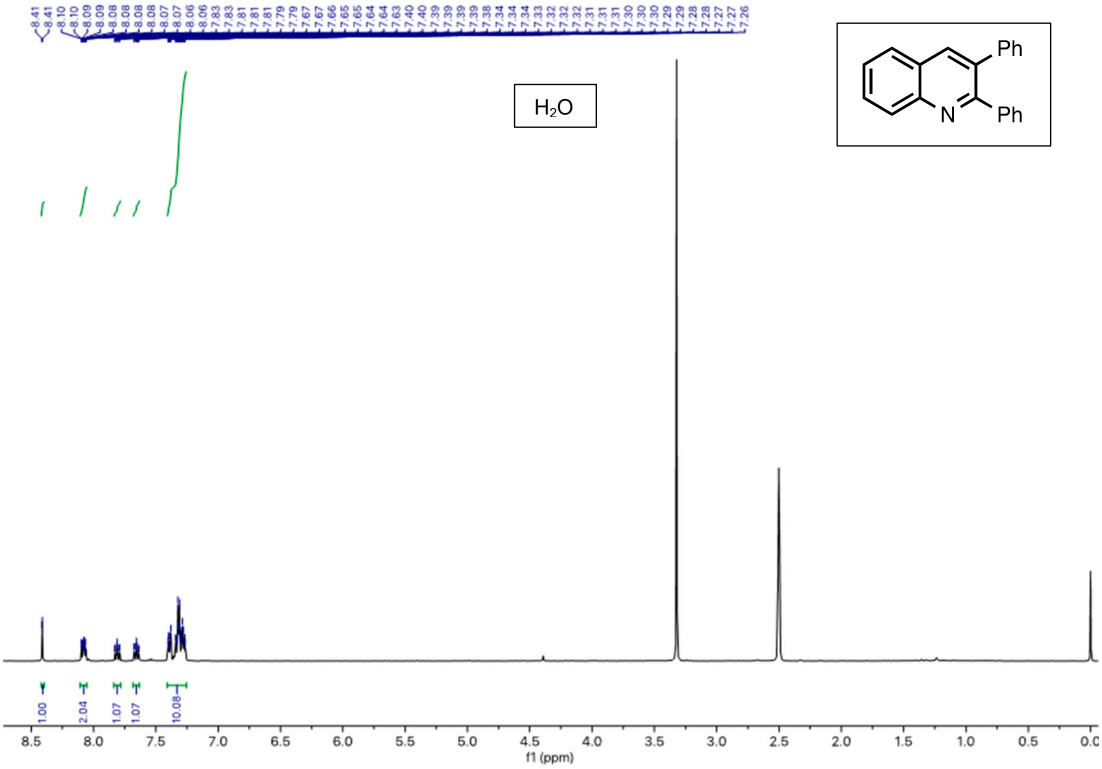

3.3.18. ^{13}C NMR for 2,3-Diphenylquinoline (Table 1, entry 18)

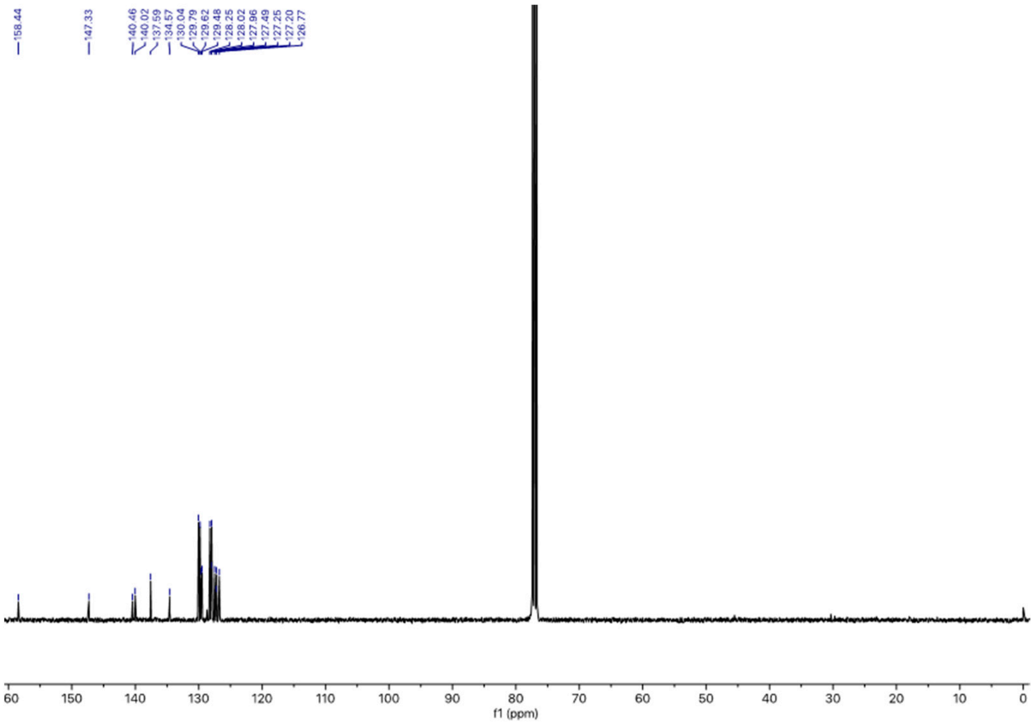

3.3.19. ^{1}H NMR for 5,6-Dihydrobenzo[*a*]acridine (Table 1, entry 19)

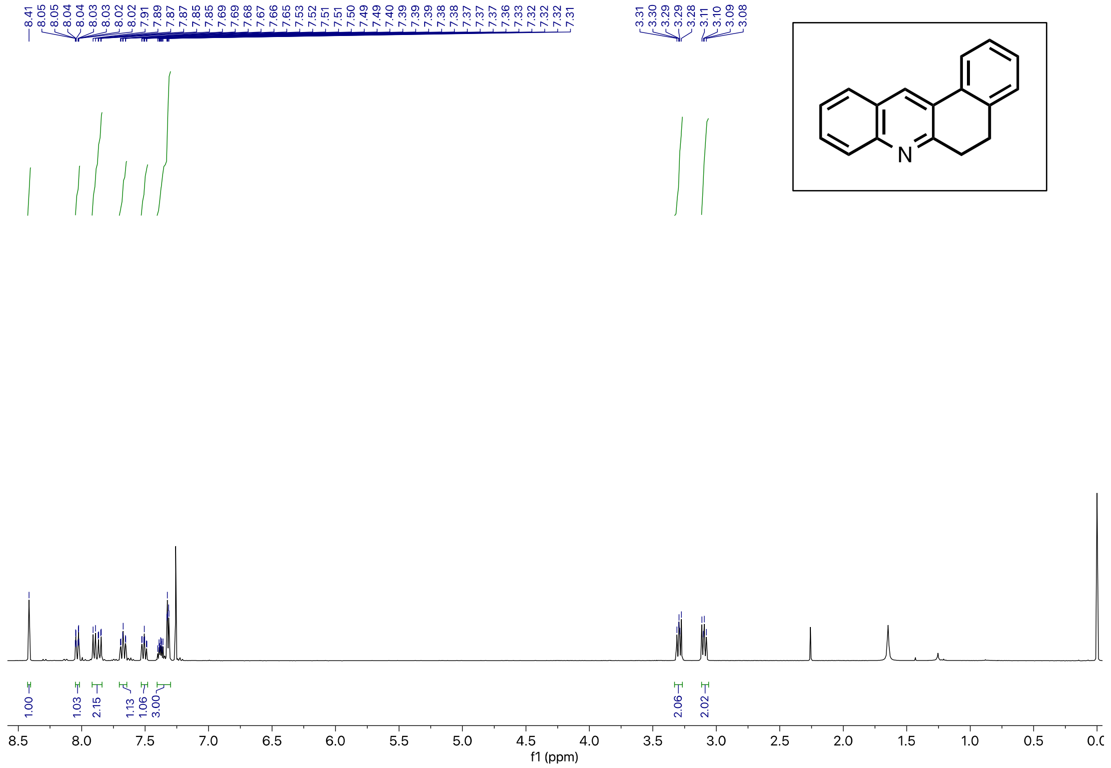

3.3.19. ^{13}C NMR for 5,6-Dihydrobenzo[*a*]acridine (Table 1, entry 19)

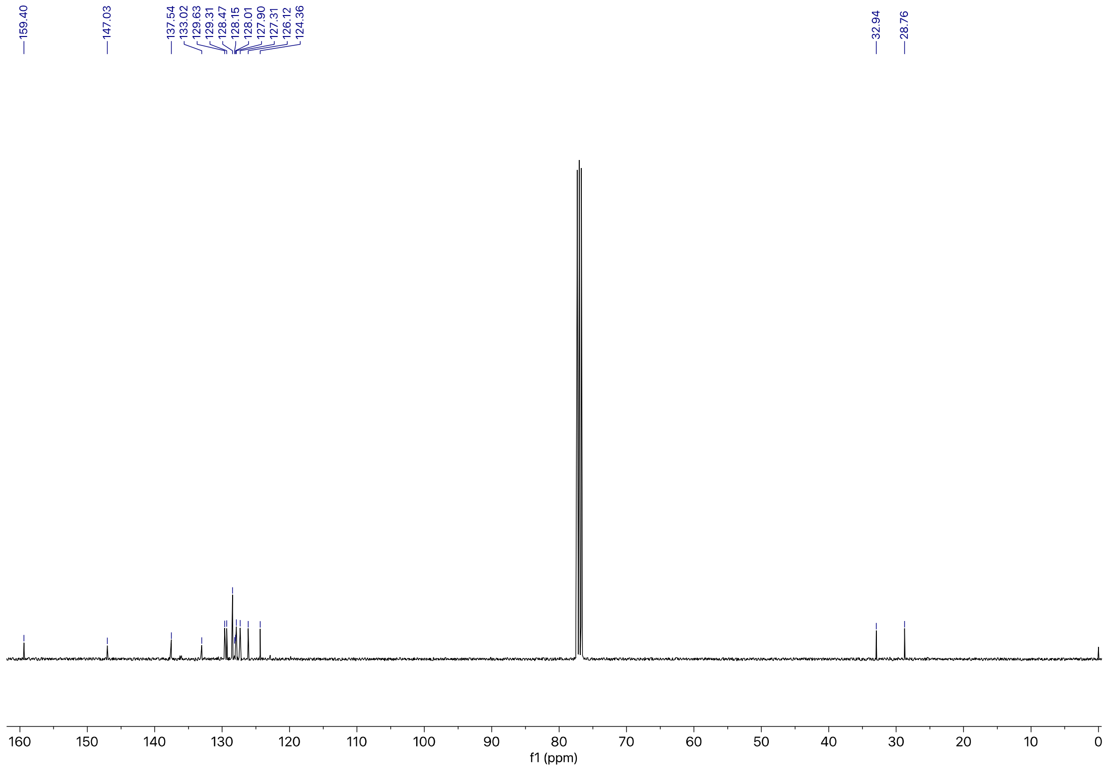

3.4.1. ^{1}H NMR for Ethyl 6-fluoro-2-(trifluoromethyl)quinoline-3-carboxylate (Table 2, entry 1)

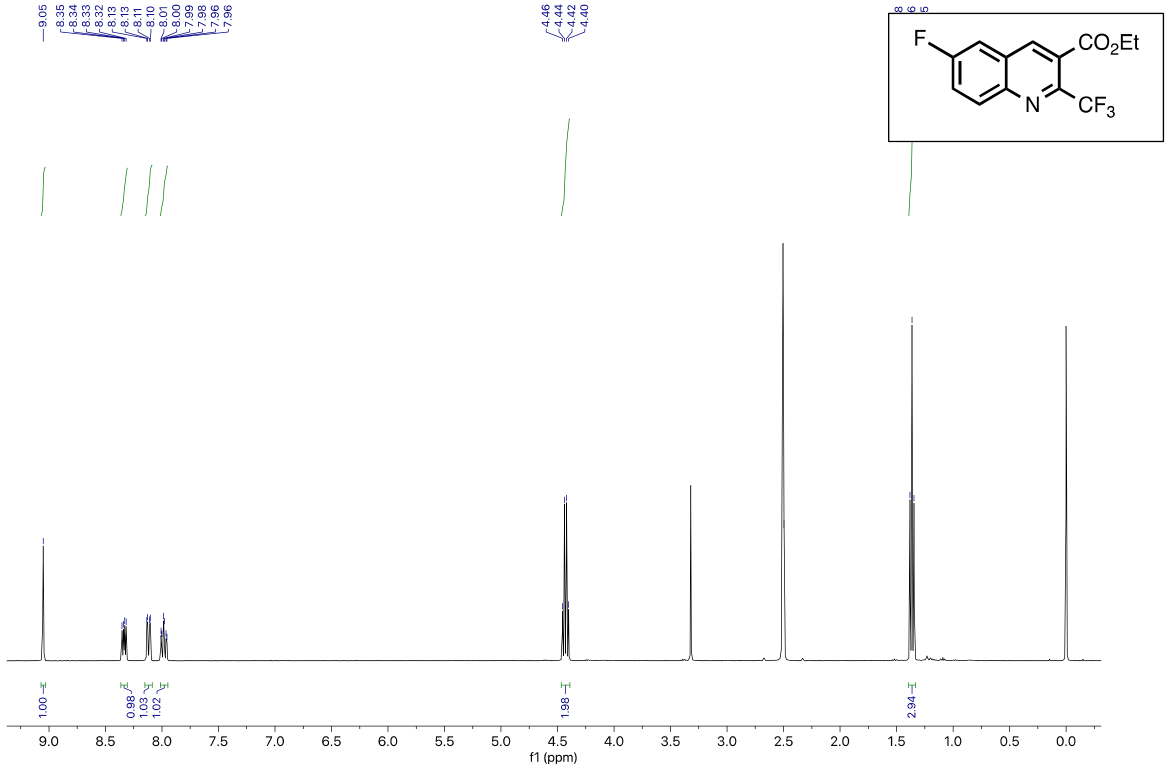

3.4.1. ^{13}C NMR for Ethyl 6-fluoro-2-(trifluoromethyl)quinoline-3-carboxylate (Table 2, entry 1)

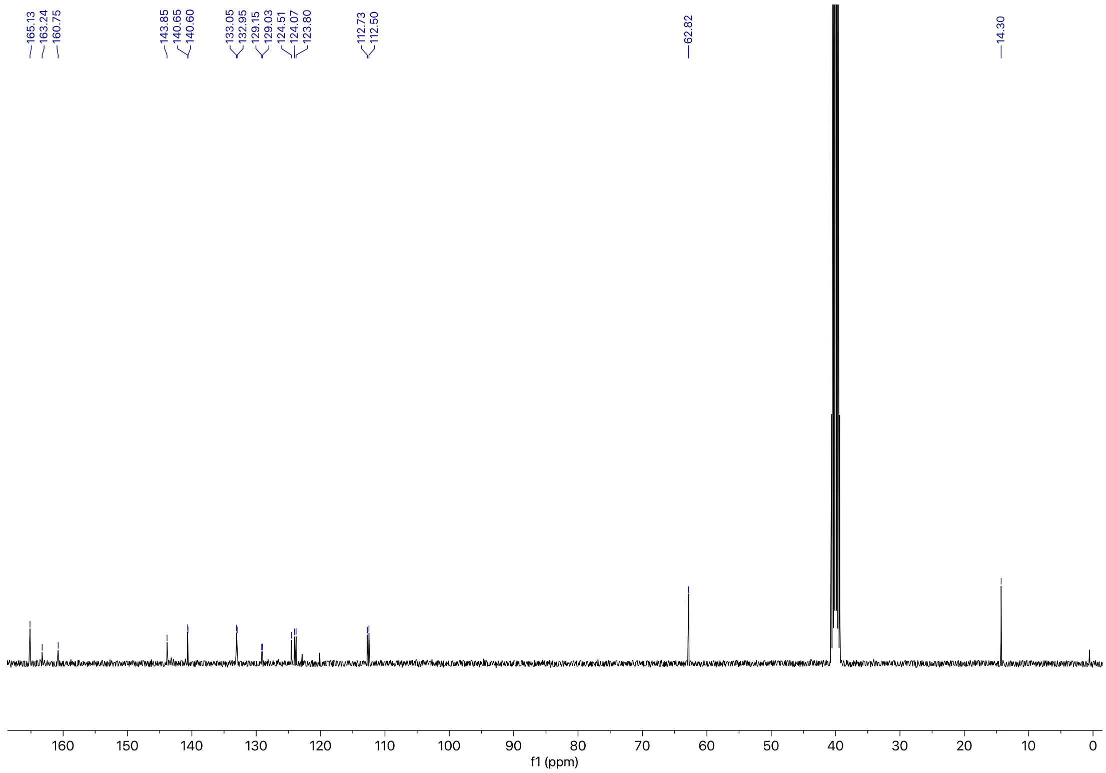

3.4.2. ^{1}H NMR for Methyl 6-fluoro-2-isopropylquinoline-3-carboxylate (Table 2, entry 2)

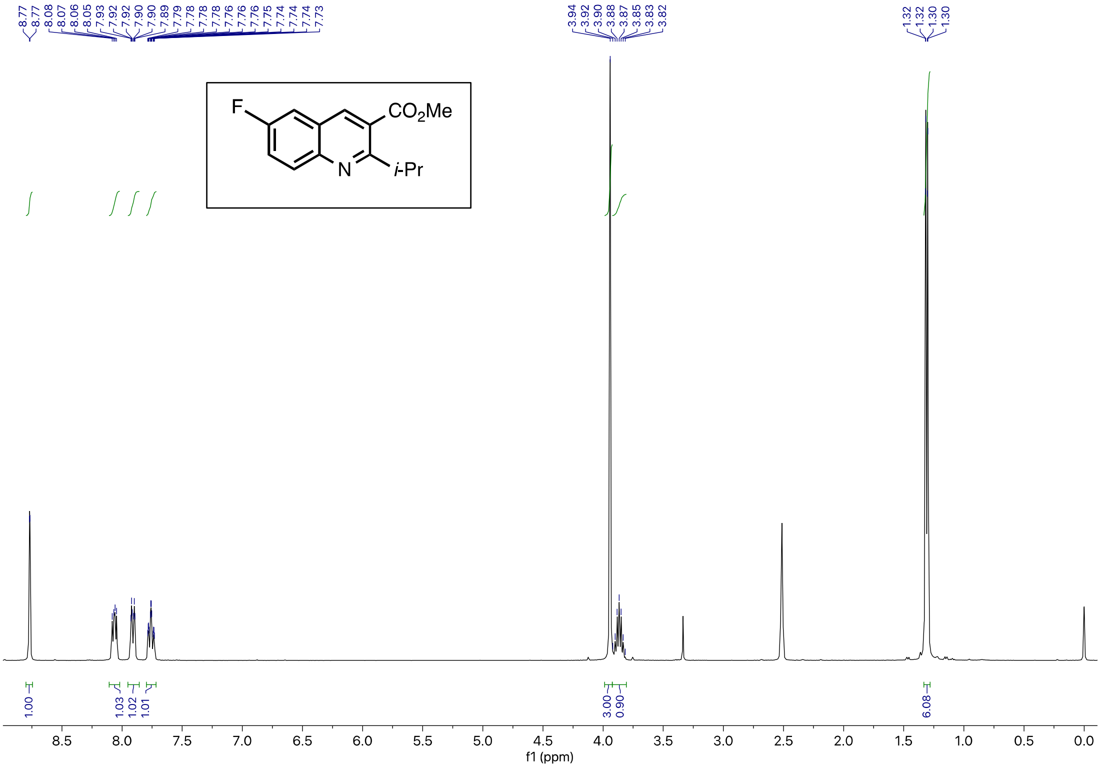

3.4.2. ^{13}C NMR for Methyl 6-fluoro-2-isopropylquinoline-3-carboxylate (Table 2, entry 2)

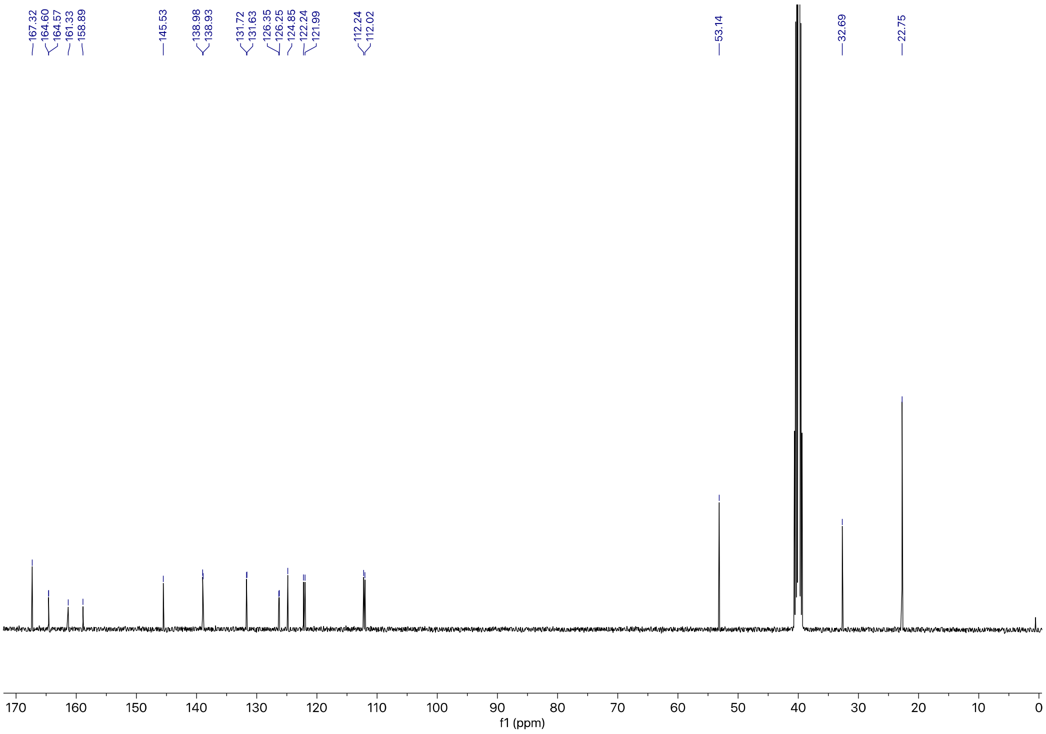

3.4.3. ^{1}H NMR for Ethyl 6-fluoro-2-phenylquinoline-3-carboxylate (Table 2, entry 3)

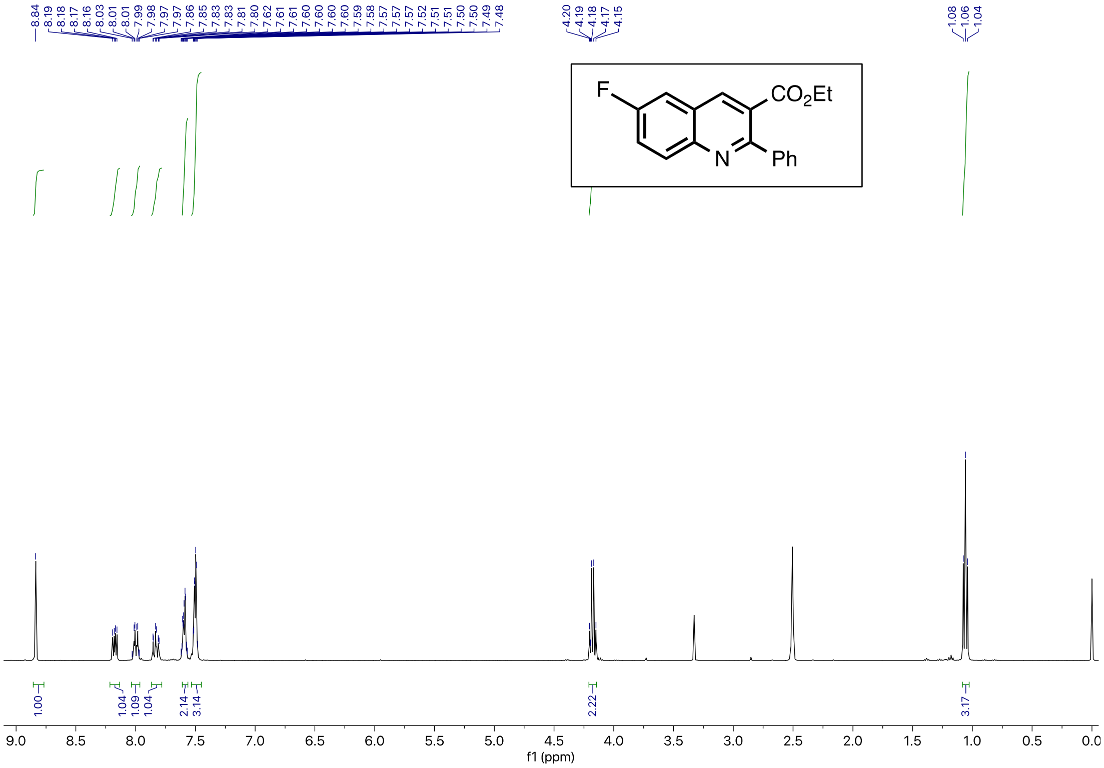

3.4.3. ^{13}C NMR for Ethyl 6-fluoro-2-phenylquinoline-3-carboxylate (Table 2, entry 3)

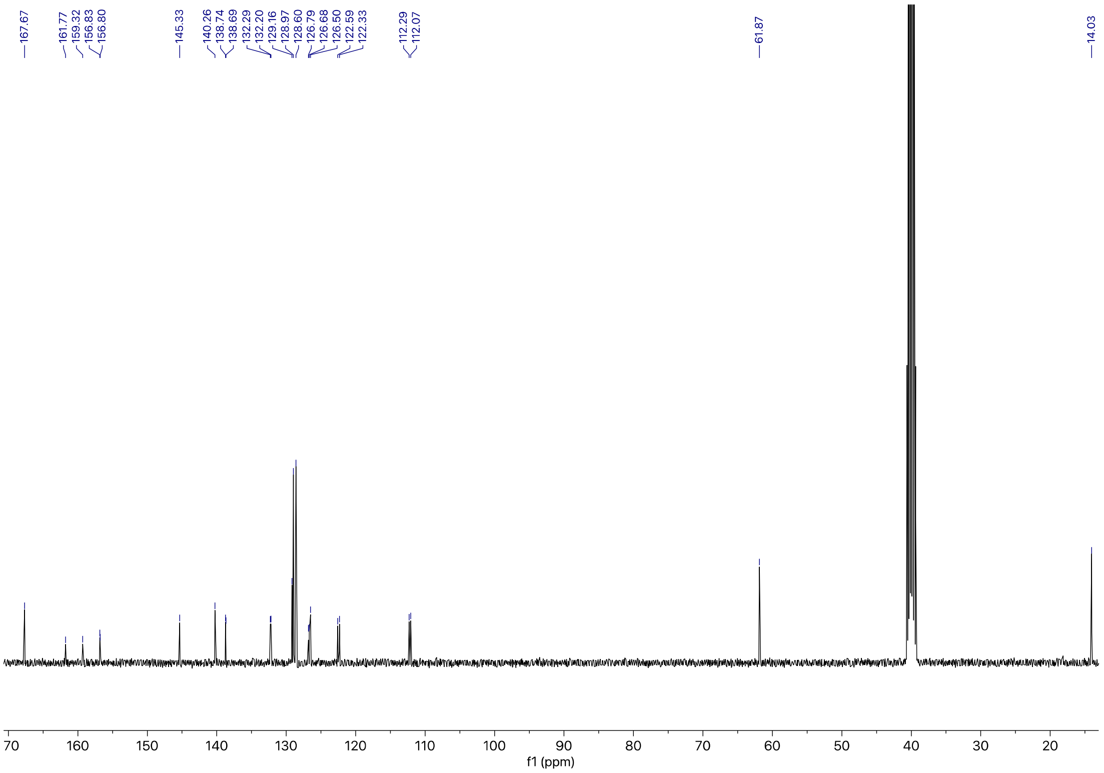

3.4.4. ^{1}H NMR for Methyl 2-benzyl-6-fluoroquinoline-3-carboxylate (Table 2, entry 4)

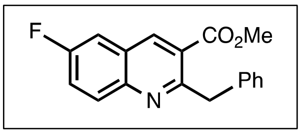

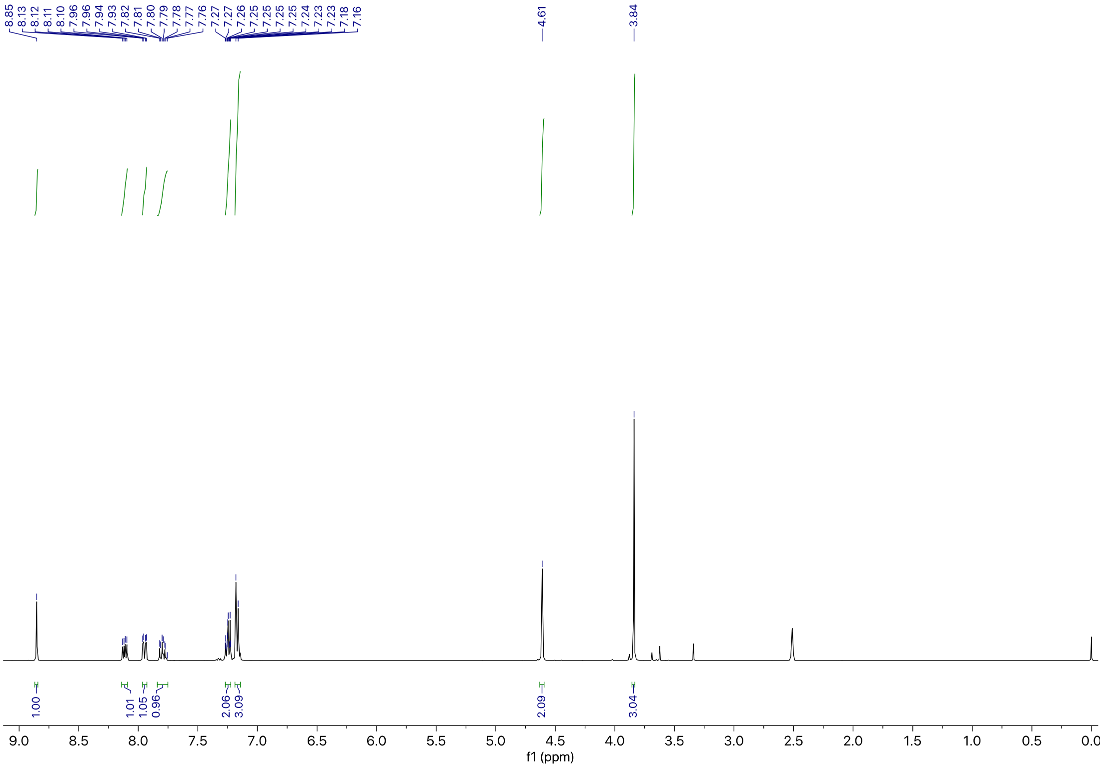

3.4.4. ^{13}C NMR for Methyl 2-benzyl-6-fluoroquinoline-3-carboxylate (Table 2, entry 4)

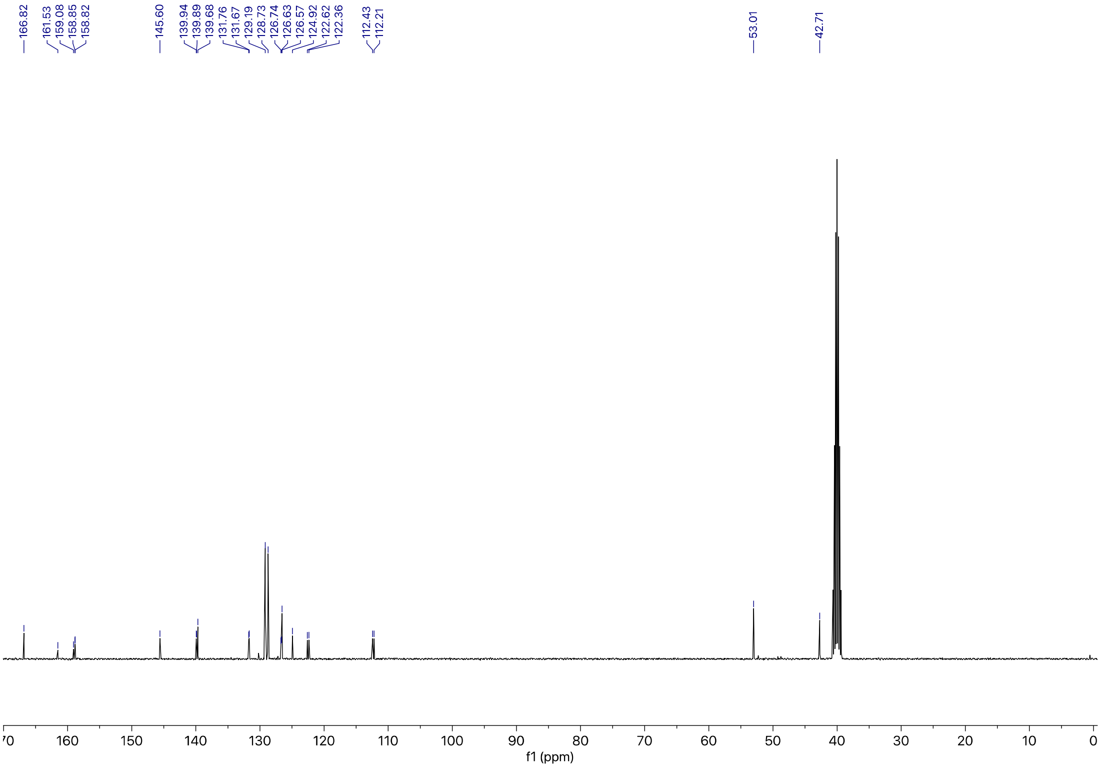

3.4.5. ^{1}H NMR for 6-Fluoro-2-methyl-3-(phenylsulfonyl)quinoline (Table 2, entry 5)

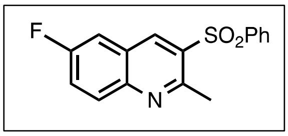

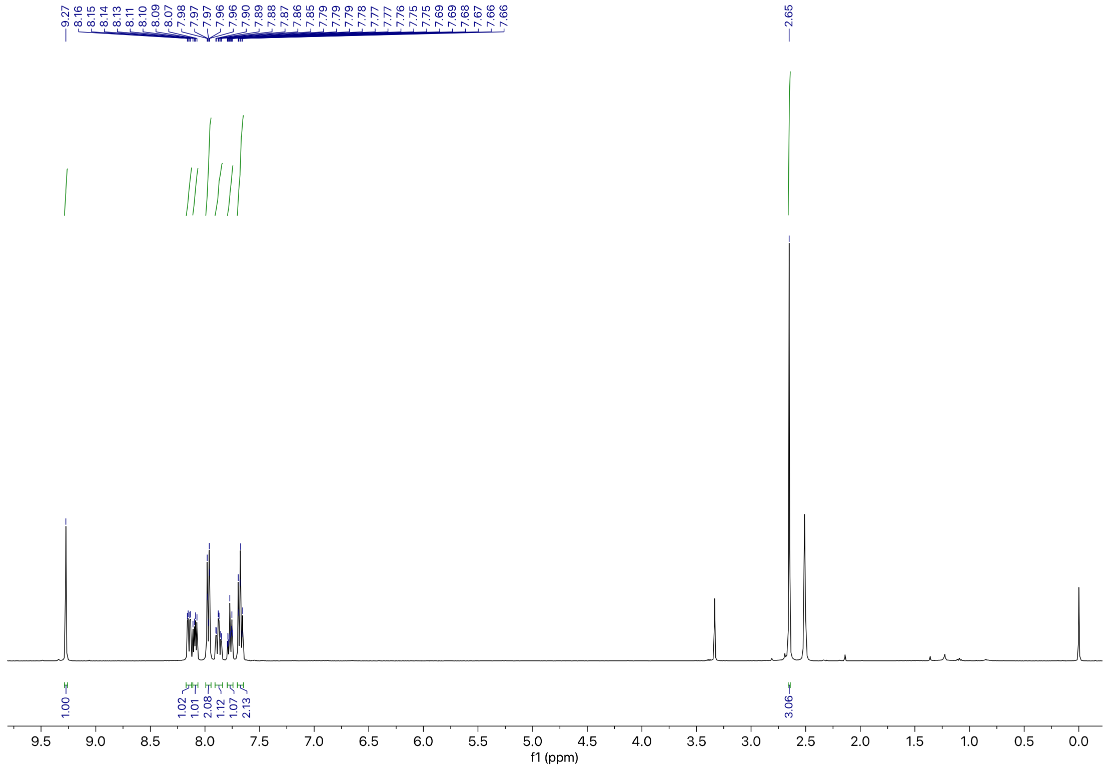

3.4.5. ^{13}C NMR for 6-Fluoro-2-methyl-3-(phenylsulfonyl)quinoline (Table 2, entry 5)

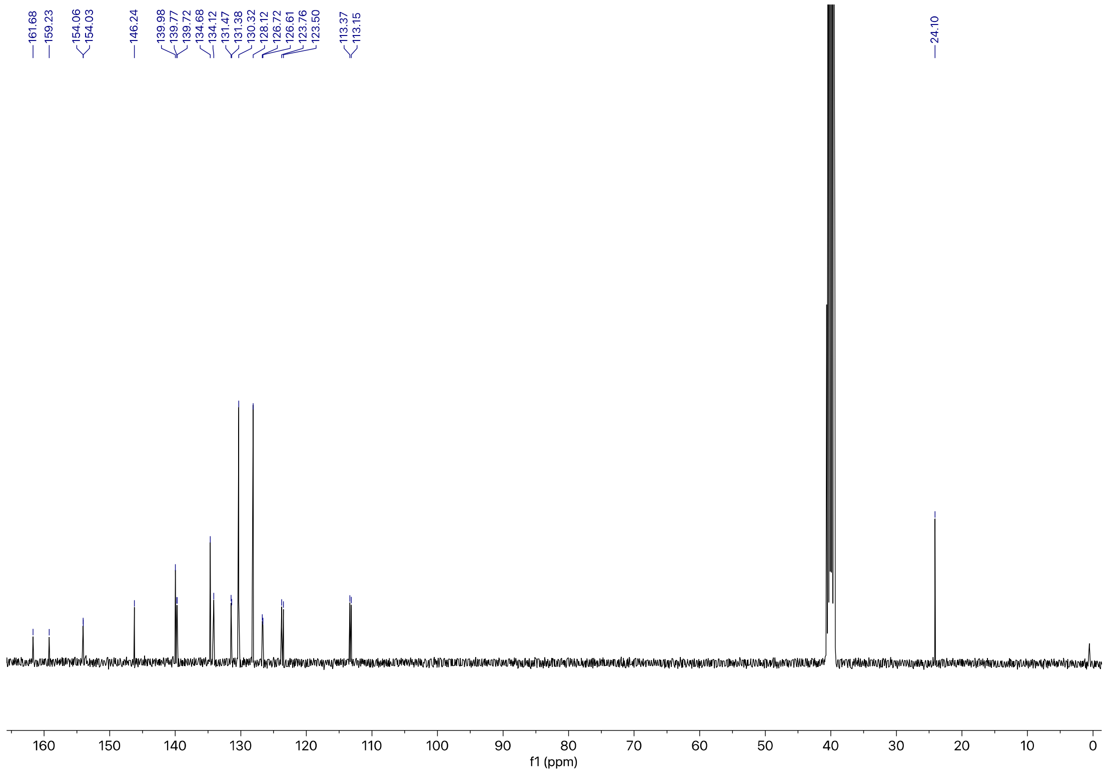

3.4.6. ^{1}H NMR for 6-Fluoro-2-phenylquinoline-3-carbonitrile (Table 2, entry 6)

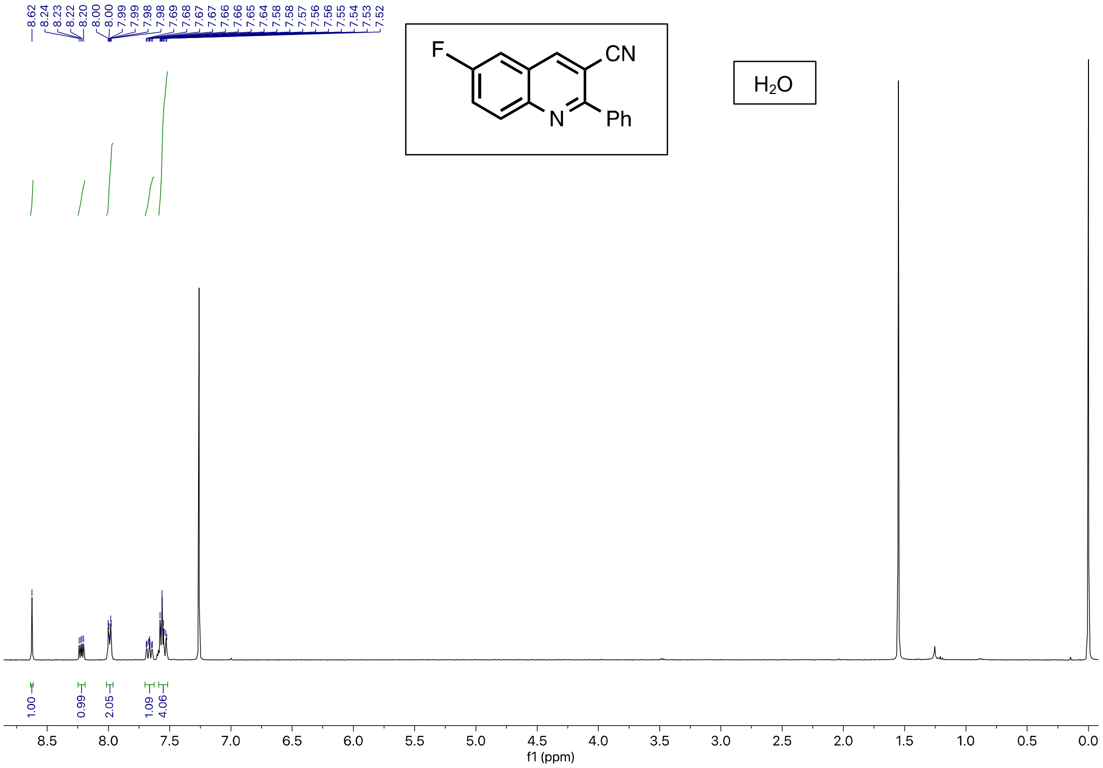

3.4.6. ^{13}C NMR for 6-Fluoro-2-phenylquinoline-3-carbonitrile (Table 2, entry 6)

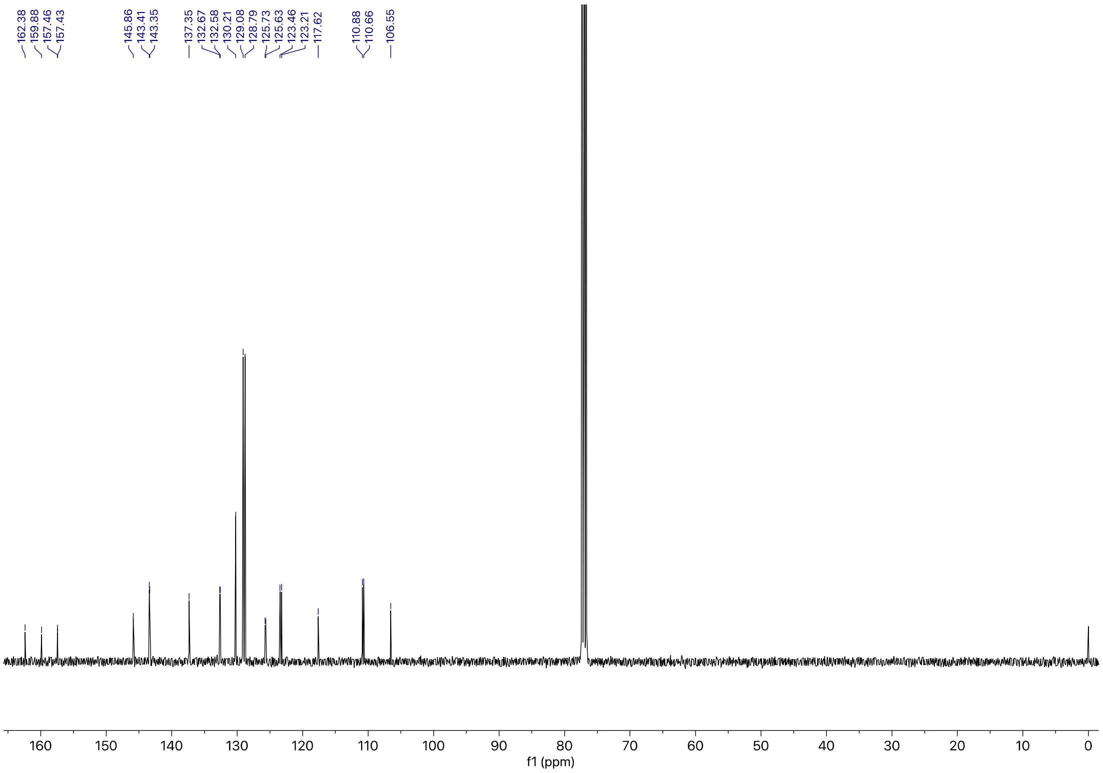

3.4.7. ^{1}H NMR for 7-Fluoro-3,3-dimethyl-3,4-dihydroacridine-1(2*H*)-one (Table 2, entry 7)

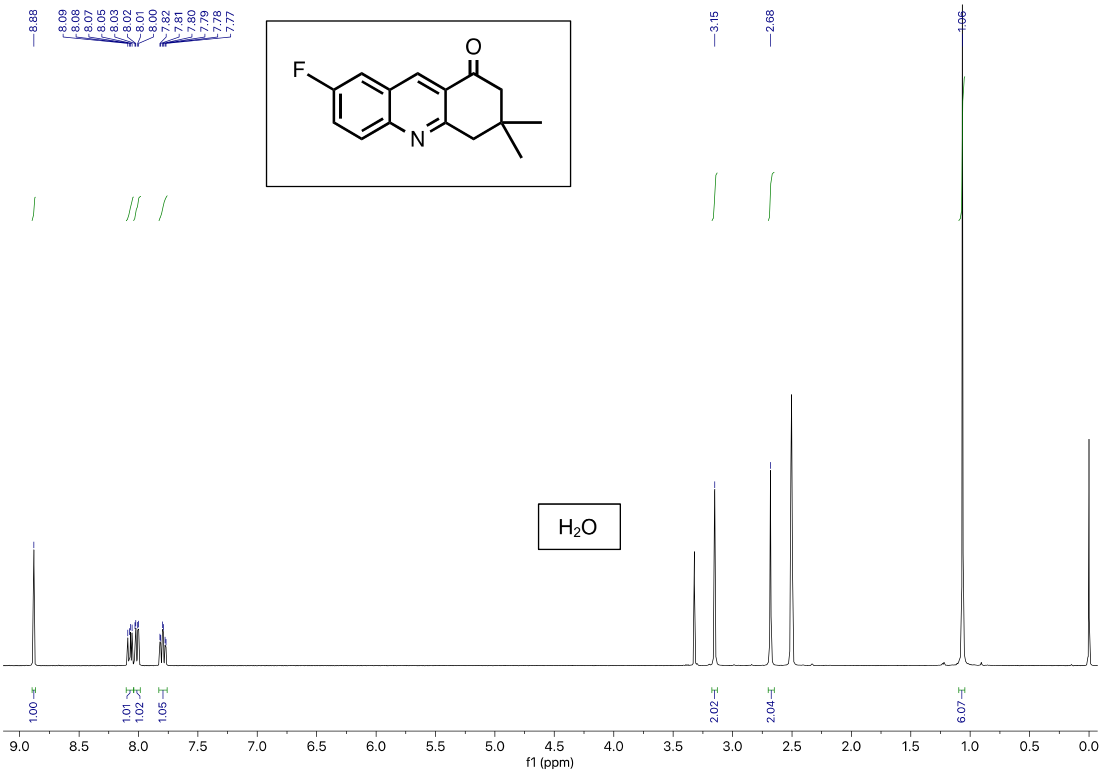

3.4.7. ^{13}C NMR for 7-Fluoro-3,3-dimethyl-3,4-dihydroacridin-1(2*H*)-one (Table 2, entry 7)

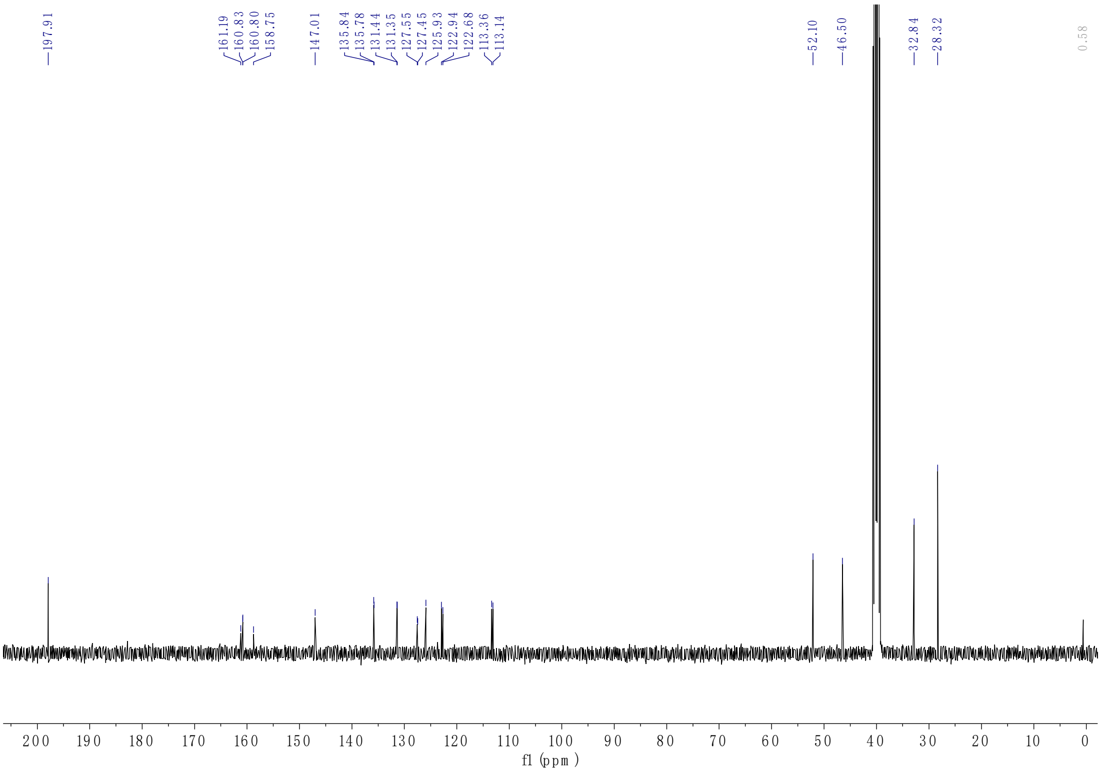

3.5.1. ^{1}H NMR for Ethyl 6-methoxy-2-(trifluoromethyl)quinoline-3-carboxylate (Table 3, entry 1)

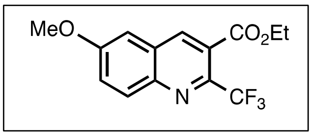

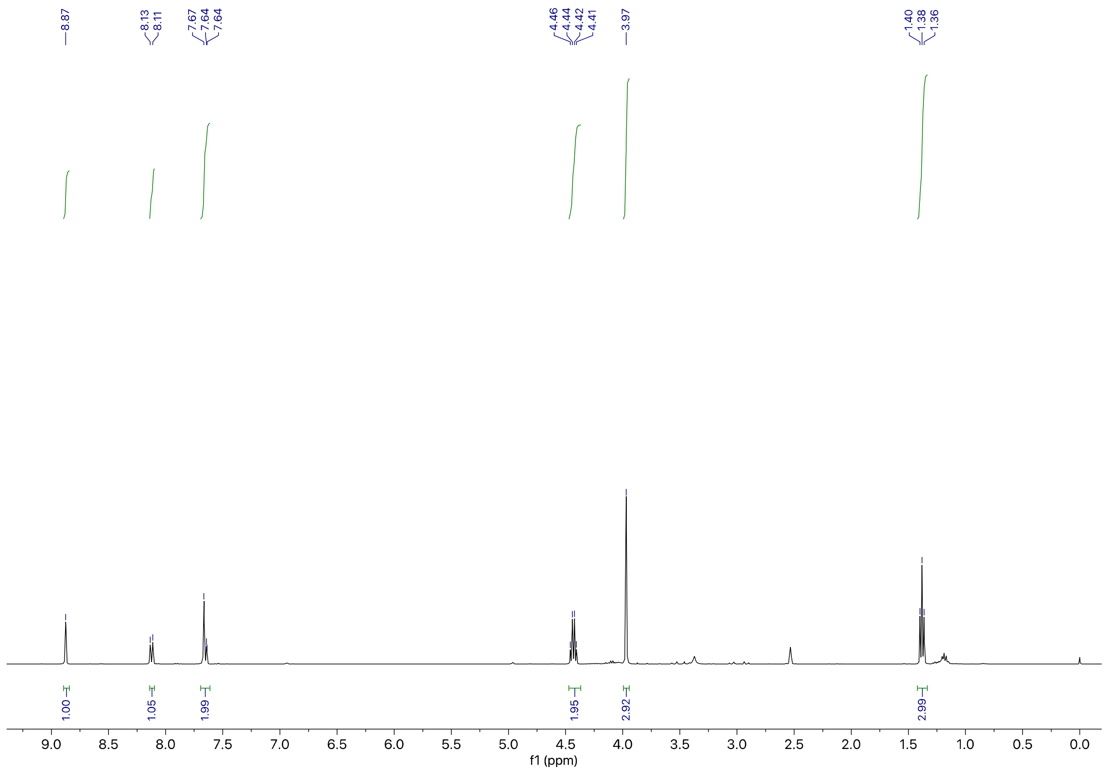

3.5.1. ^{13}C NMR for Ethyl 6-methoxy-2-(trifluoromethyl)quinoline-3-carboxylate (Table 3, entry 1)

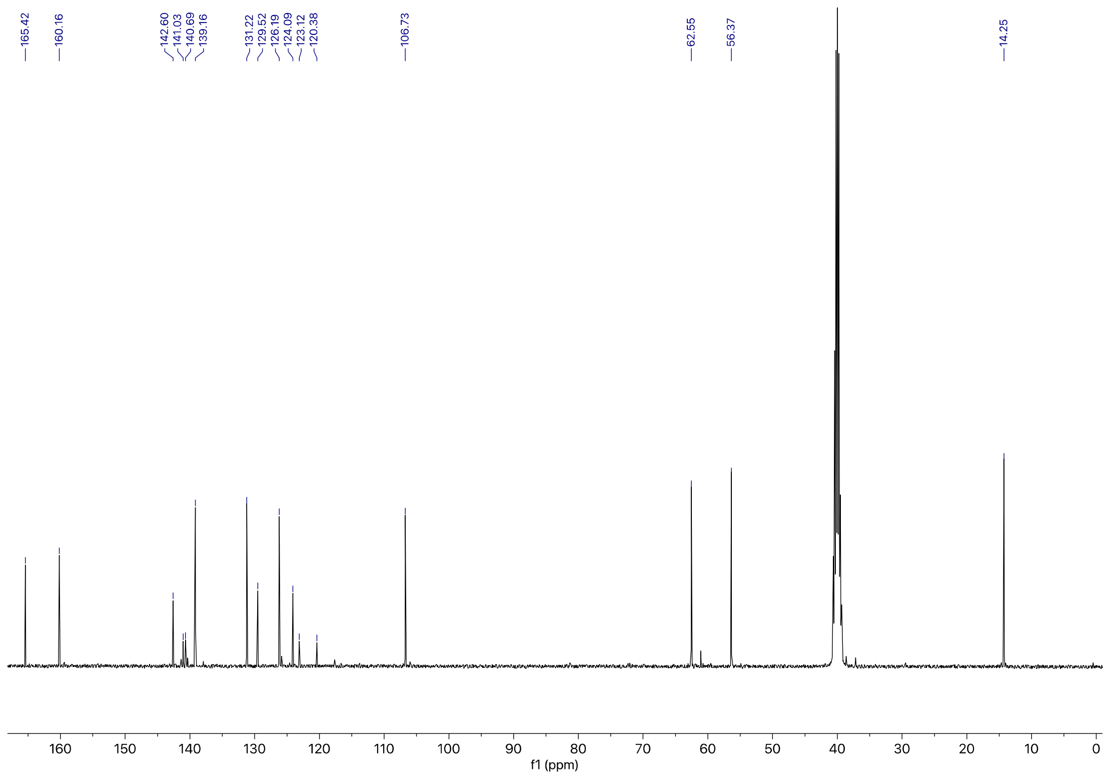

3.5.2. ^{1}H NMR for Methyl 2-isopropyl-6-methoxyquinoline-3-carboxylate (Table 3, entry 2)

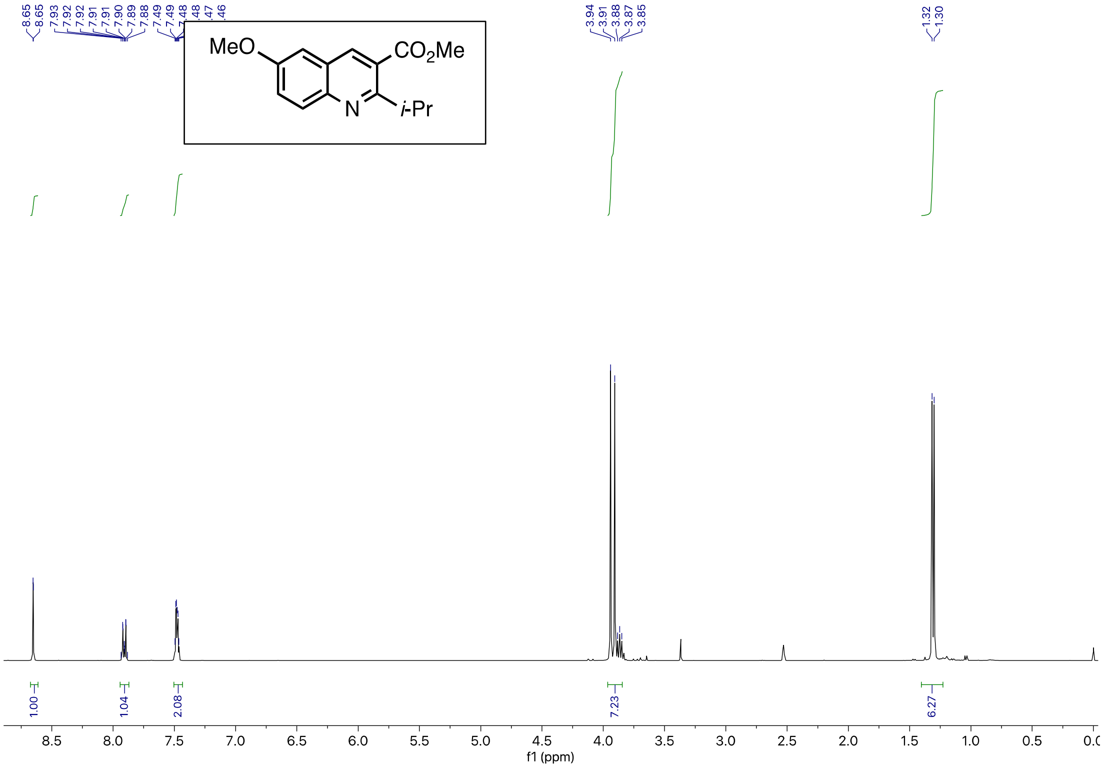

3.5.2. ^{13}C NMR for Methyl 2-isopropyl-6-methoxyquinoline-3-carboxylate (Table 3, entry 2)

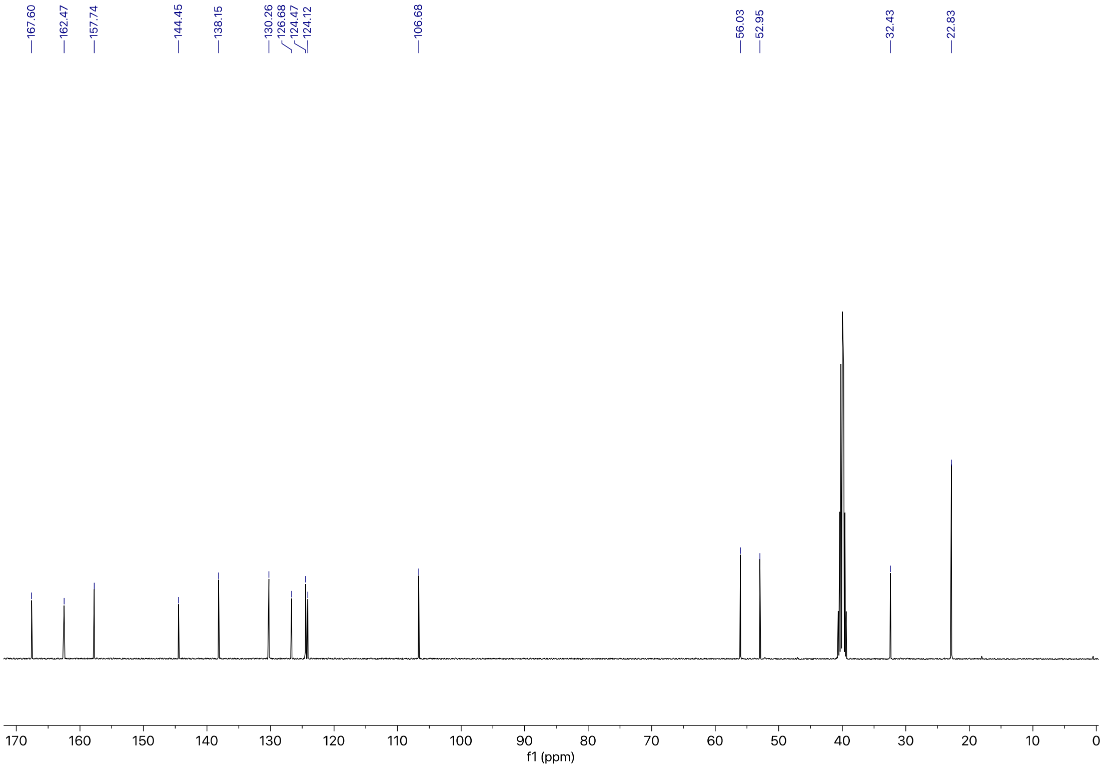

3.5.3. ^{1}H NMR for Ethyl 6-methoxy-2-phenylquinoline-3-carboxylate (Table 3, entry 3)

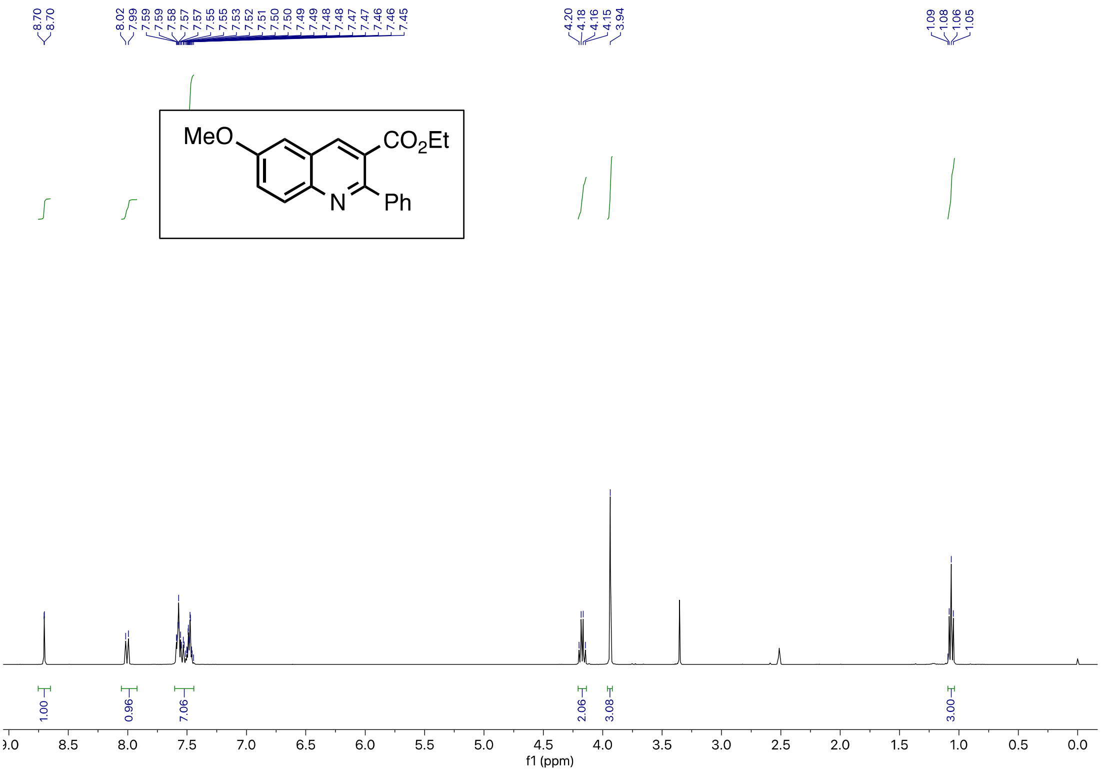

3.5.3. ^{13}C NMR for Ethyl 6-methoxy-2-phenylquinoline-3-carboxylate (Table 3, entry 3)

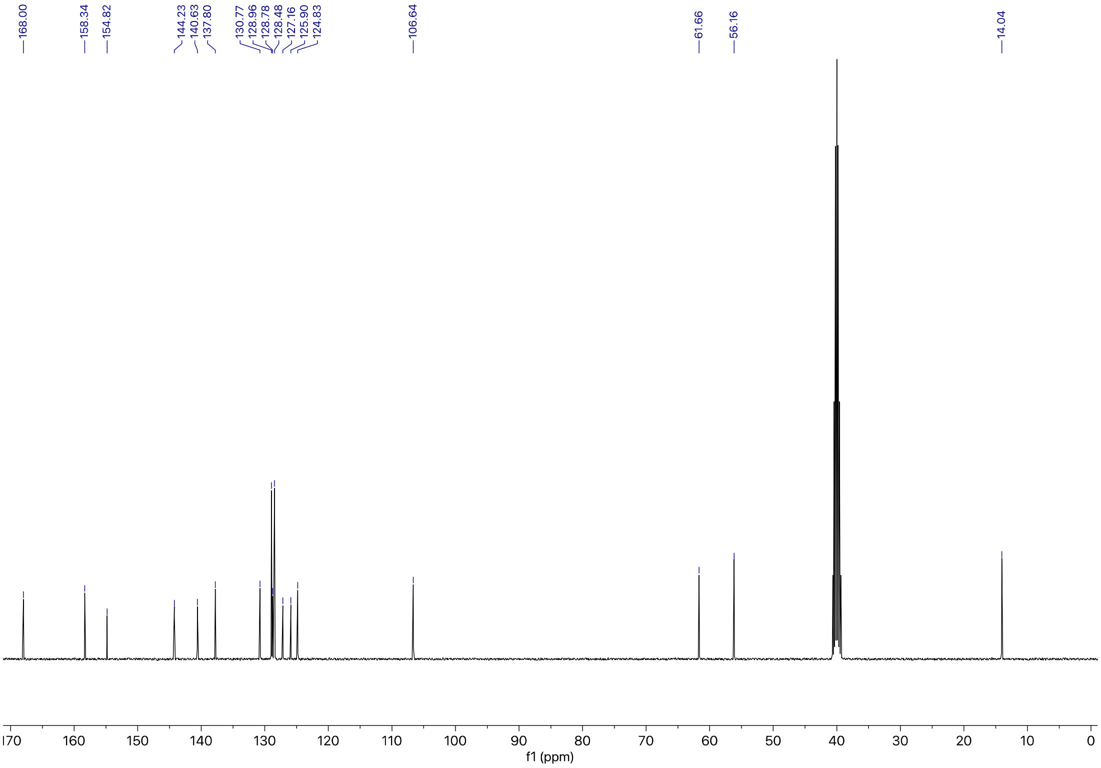

3.5.4. ^{1}H NMR for Methyl 2-benzyl-6-methoxyquinoline-3-carboxylate (Table 3, entry 4)

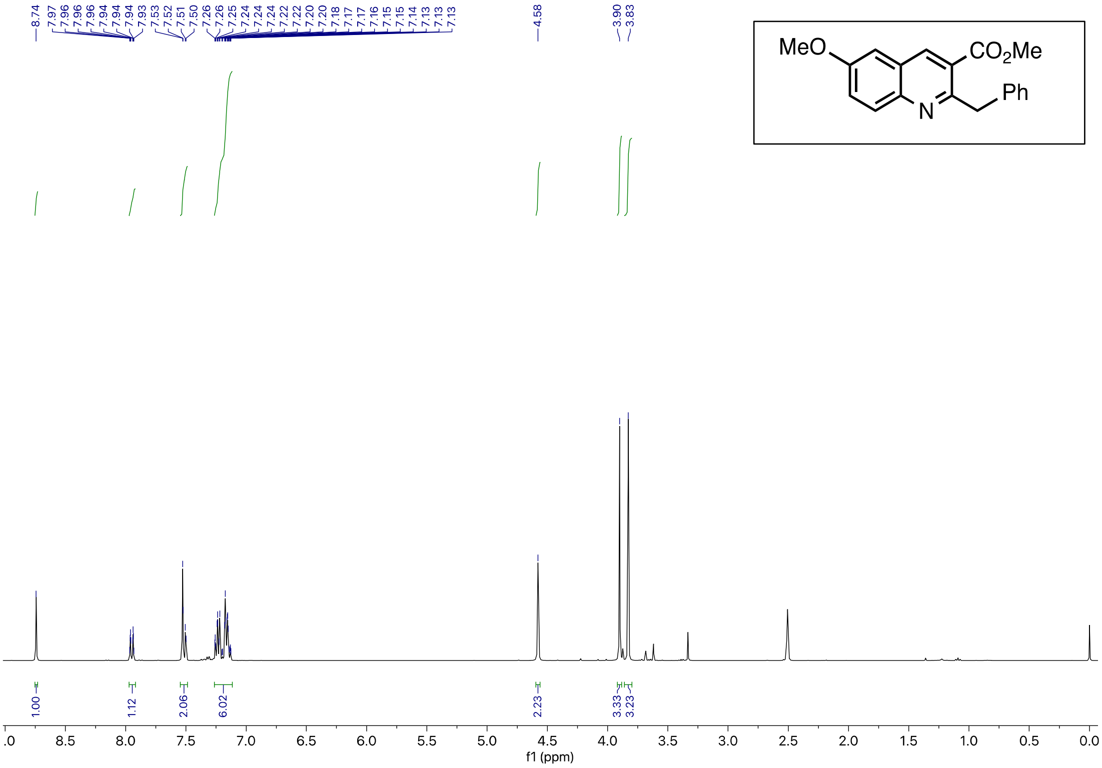

3.5.4. ^{13}C NMR for Methyl 2-benzyl-6-methoxyquinoline-3-carboxylate (Table 3, entry 4)

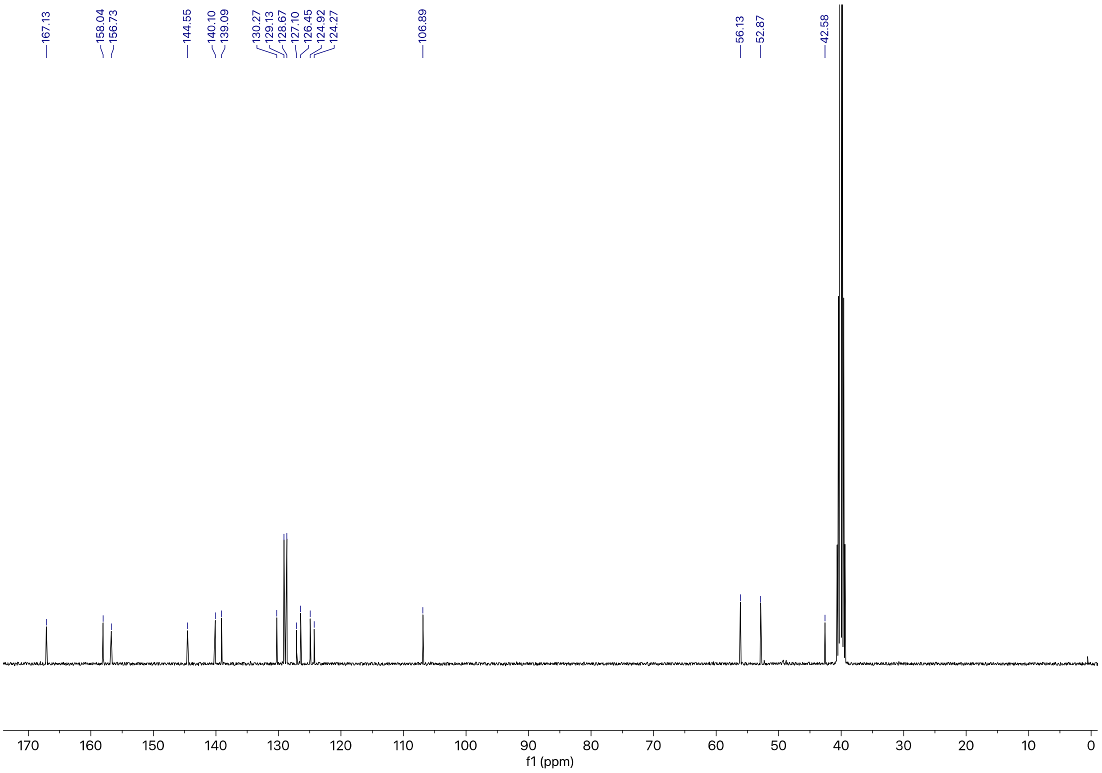

3.5.5. ^{1}H NMR for 6-Methoxy-2-methyl-3-(phenylsulfonyl)quinoline (Table 3, entry 5)

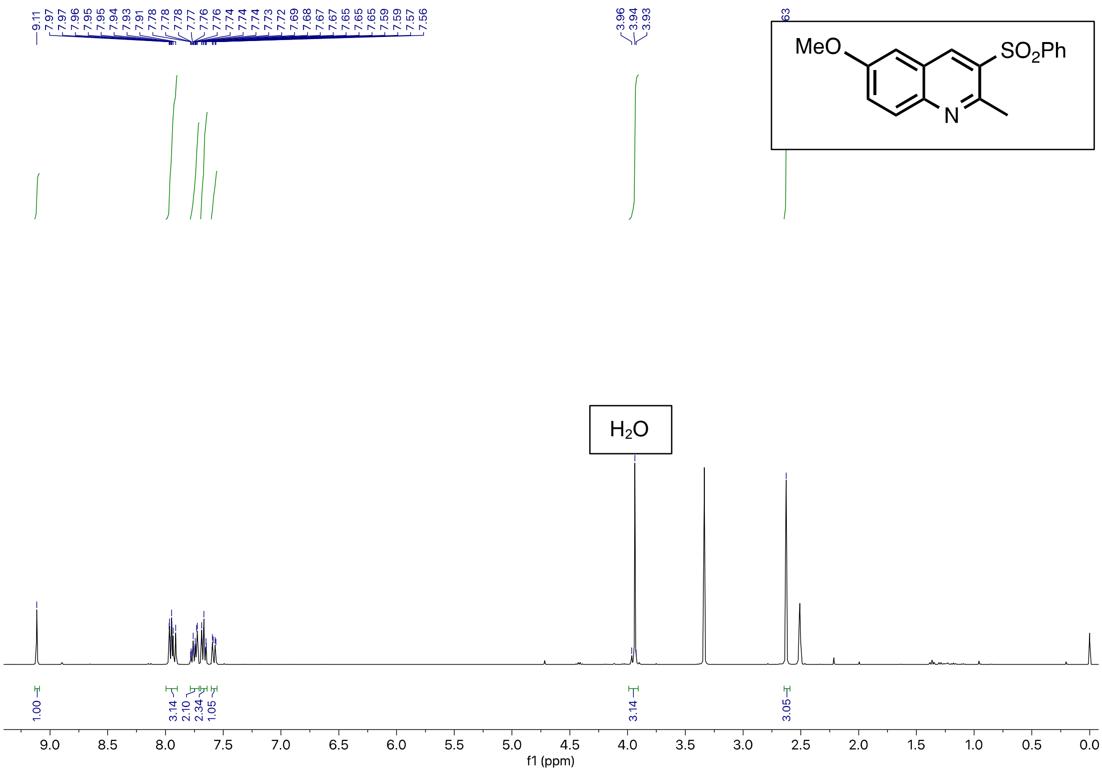

3.5.5. ^{13}C NMR for 6-Methoxy-2-methyl-3-(phenylsulfonyl)quinoline (Table 3, entry 5)

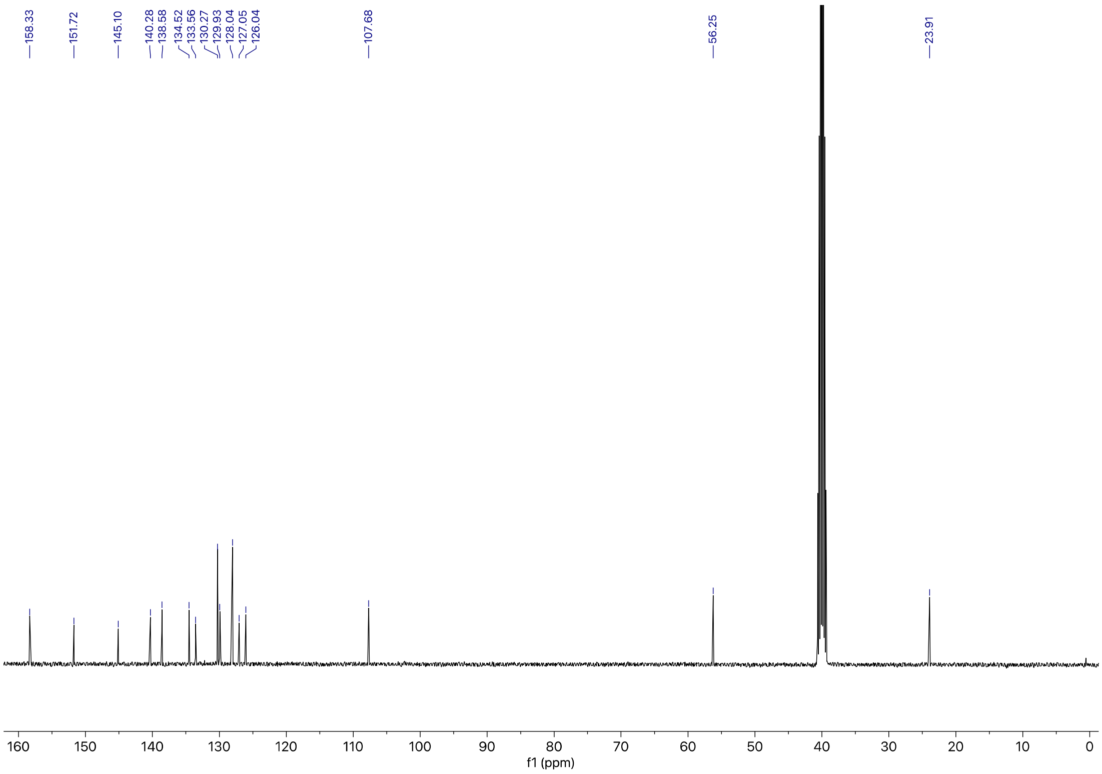

3.5.6. ^{1}H NMR for 6-Methoxy-2-phenylquinoline-3-carbonitrile (Table 3, entry 6)

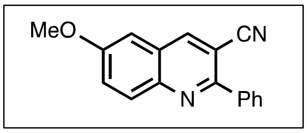

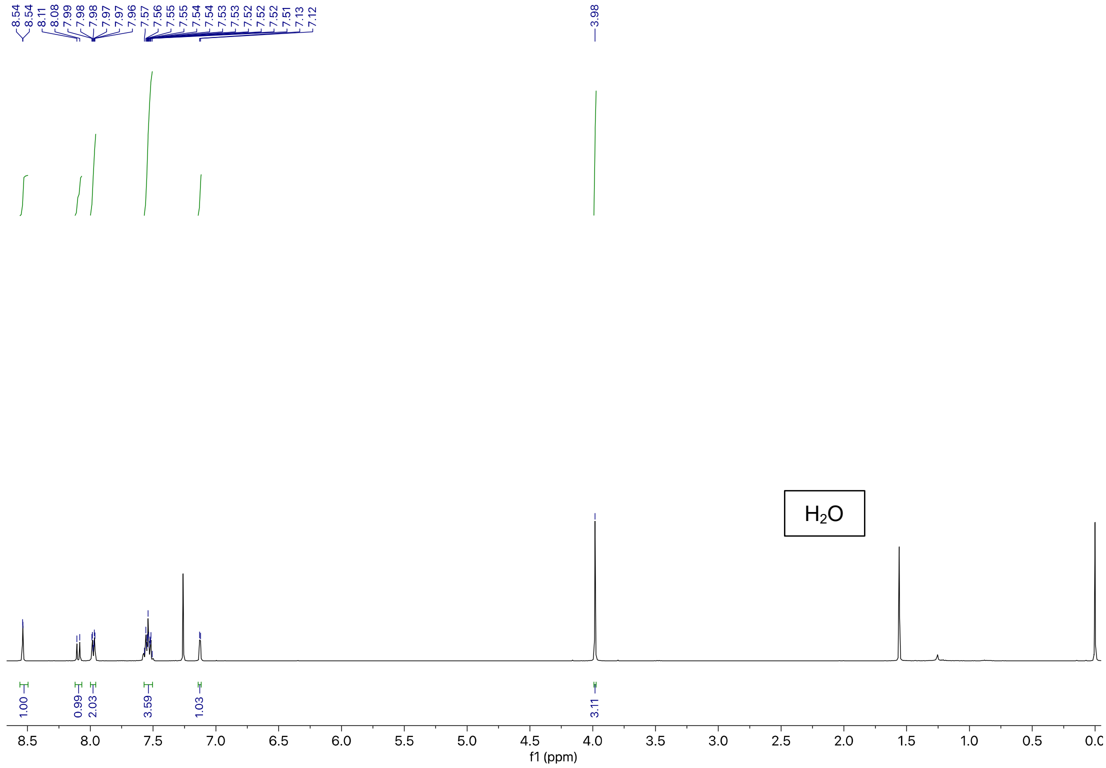

3.5.6. ^{13}C NMR for 6-Methoxy-2-phenylquinoline-3-carbonitrile (Table 3, entry 6)

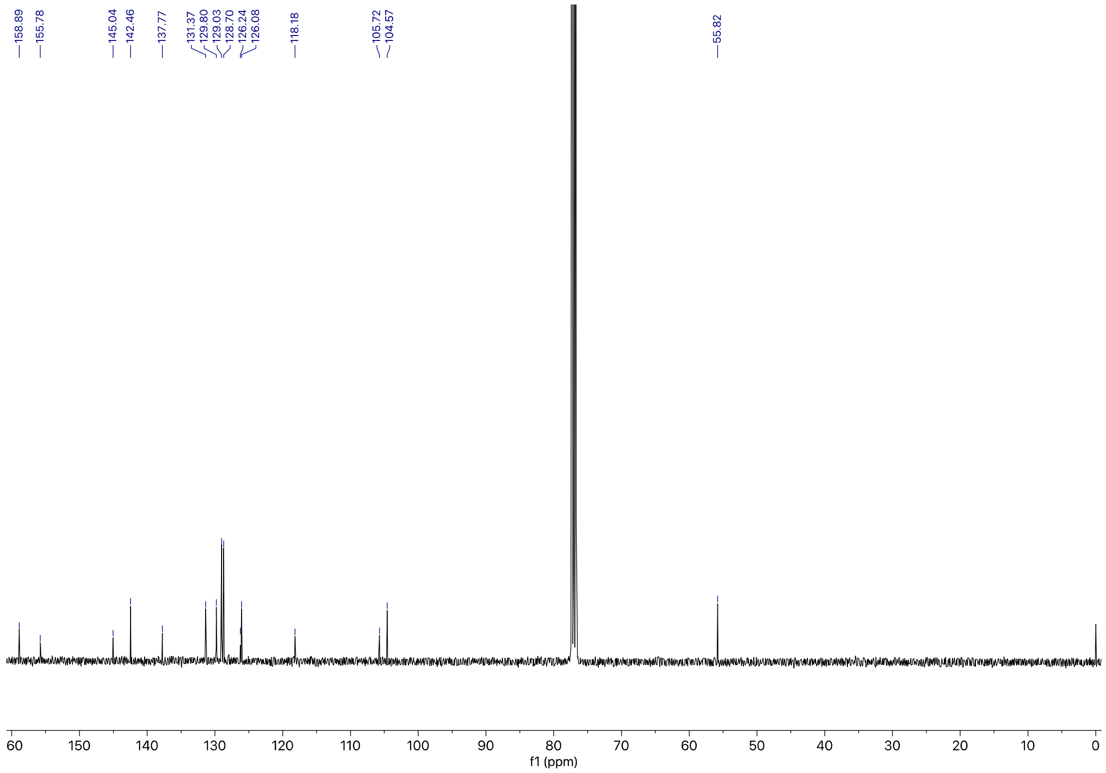

3.5.7. ^{1}H NMR for 7-Methoxy-3,3-dimethyl-3,4-dihydroacridine-1(2*H*)-one (Table 3, entry 7)

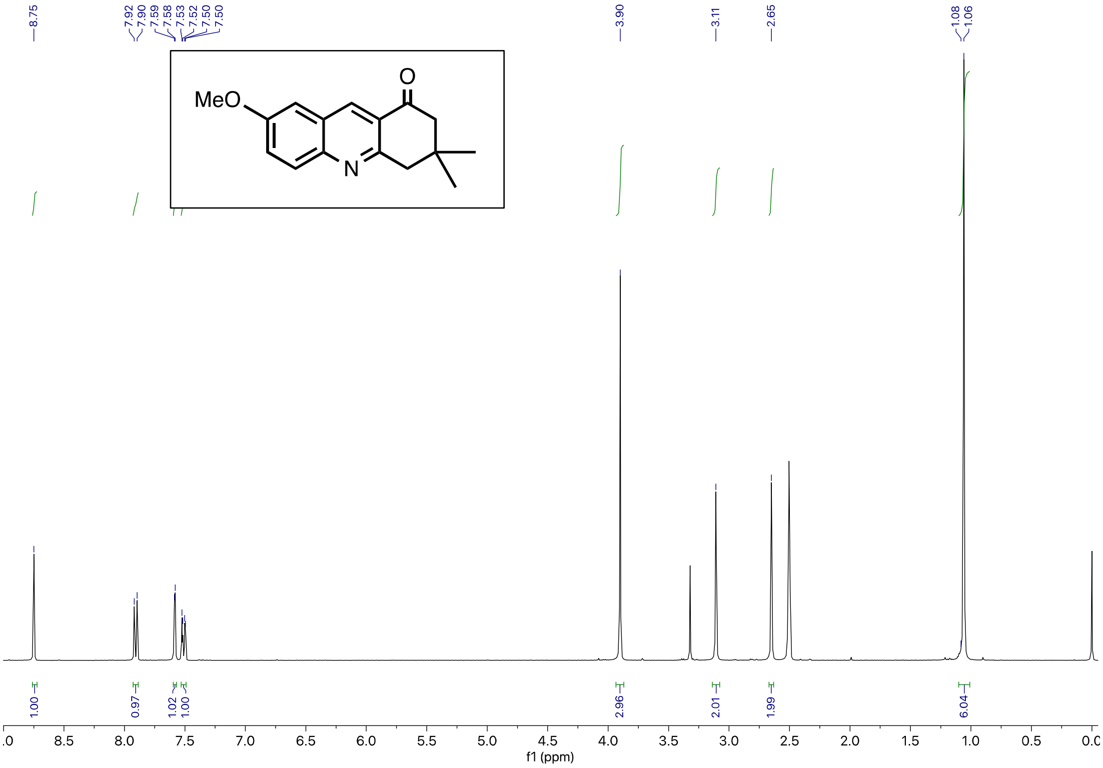

3.5.7. ^{13}C NMR for 7-Methoxy-3,3-dimethyl-3,4-dihydroacridine-1(2*H*)-one (Table 3, entry 7)

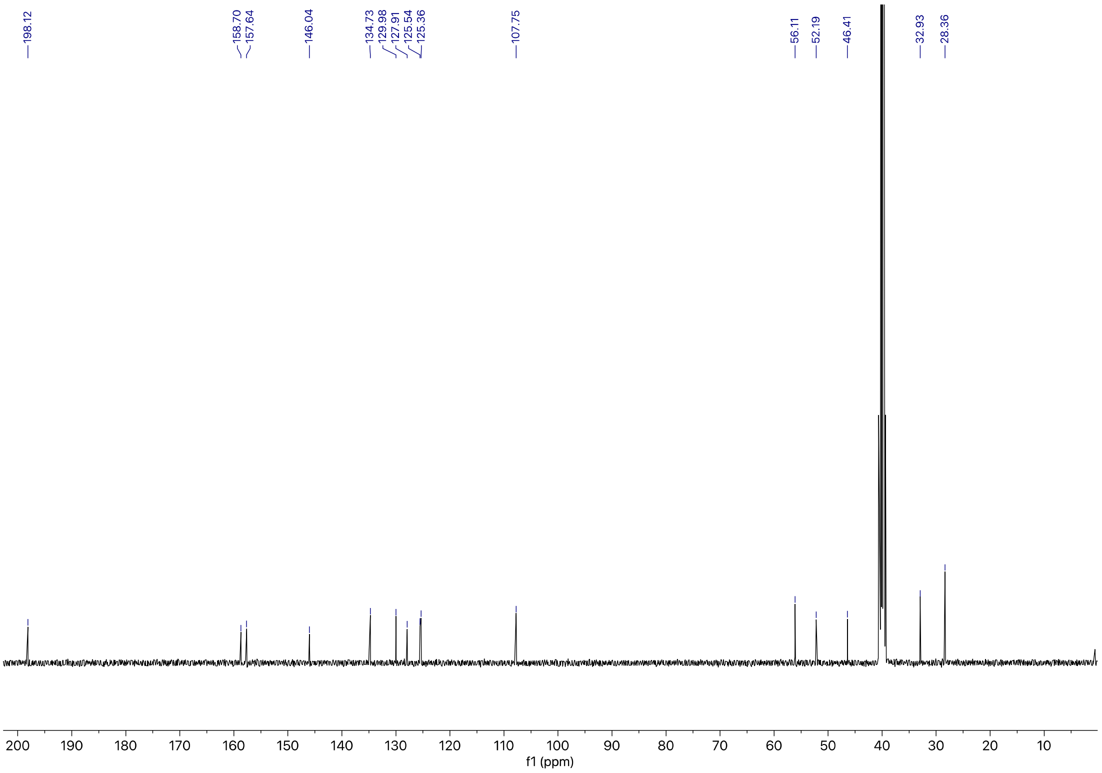

3.6.1. ^{1}H NMR for 4-Methyl-3-propionylquinolin-2(1*H*)-one (Table 4, entry 1)

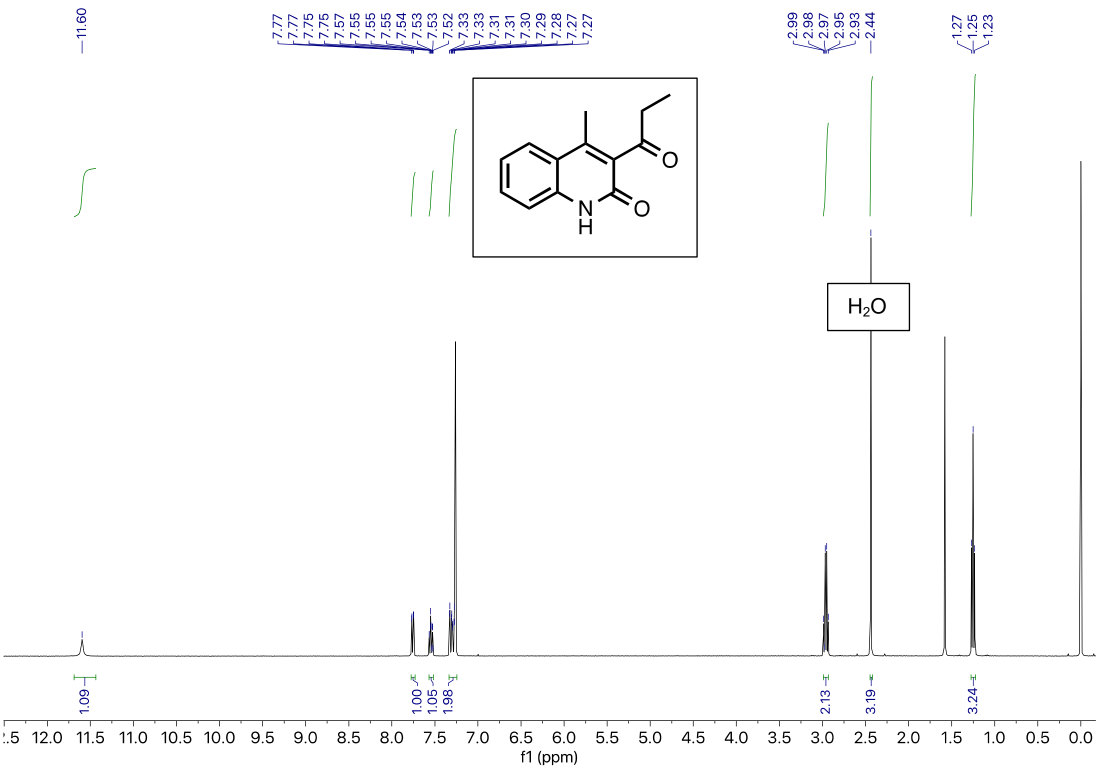

3.6.1. ^{13}C NMR for 4-Methyl-3-propionylquinolin-2(1*H*)-one (Table 4, entry 1)

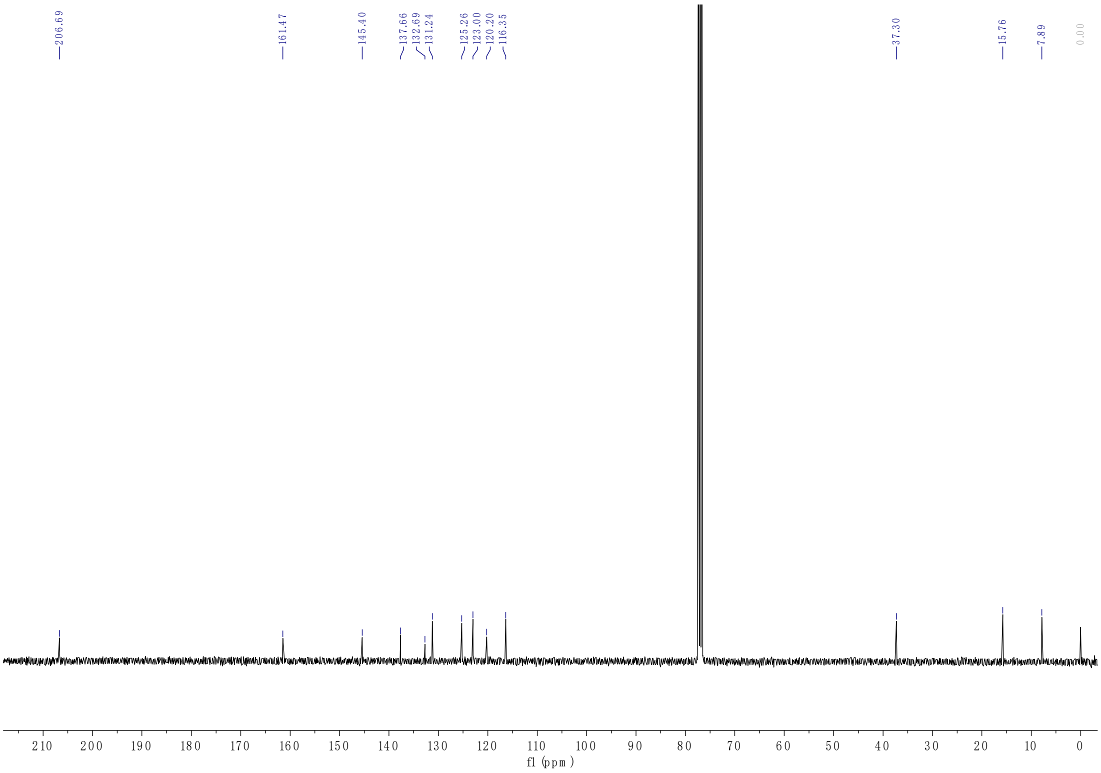

3.6.2. ^{1}H NMR for 3-Benzoyl-4-methylquinolin-2(1*H*)-one (Table 4, entry 2)

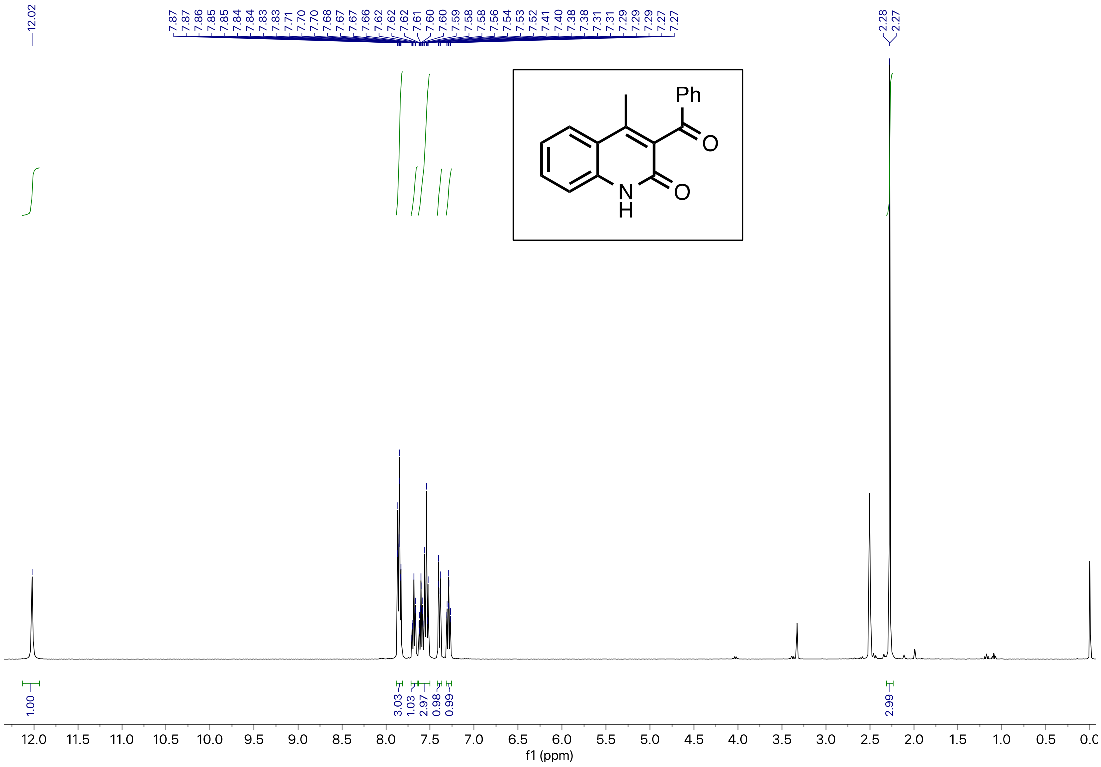

3.6.2. ^{13}C NMR for 3-Benzoyl-4-methylquinolin-2(1*H*)-one (Table 4, entry 2)

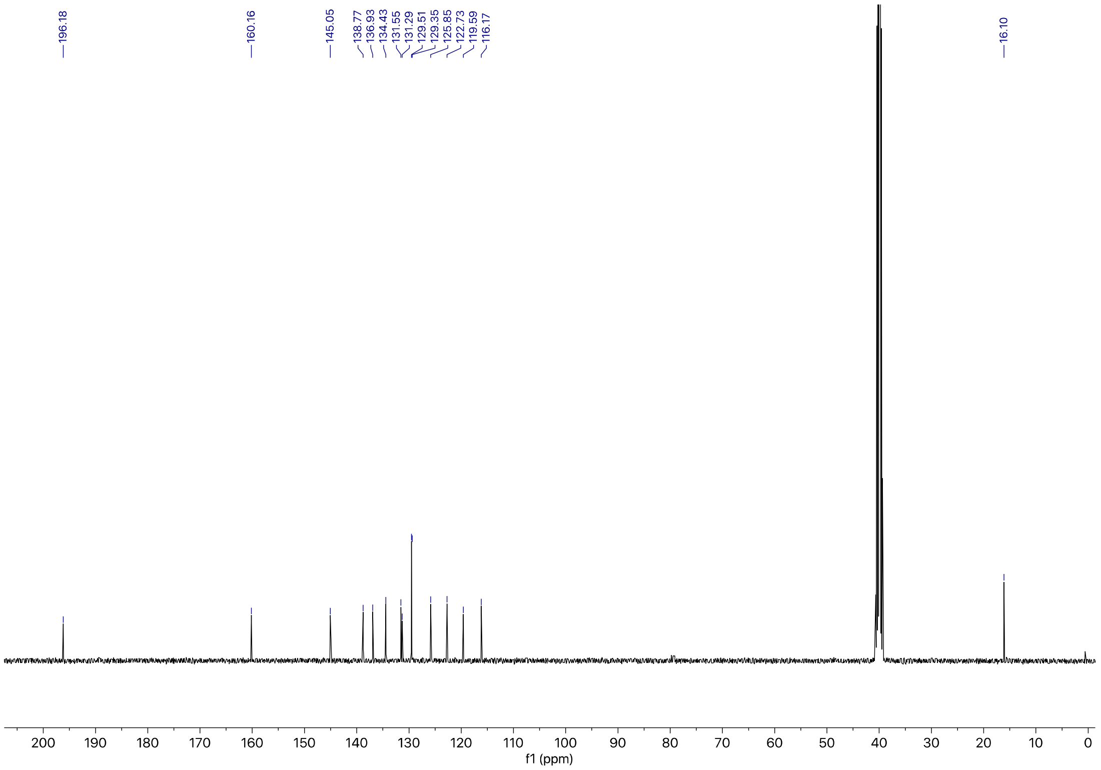

3.6.3. ^{1}H NMR for 4-Methyl-2-phenylquinoline-3-carbonitrile (Table 4, entry 3)

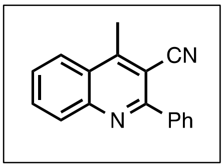

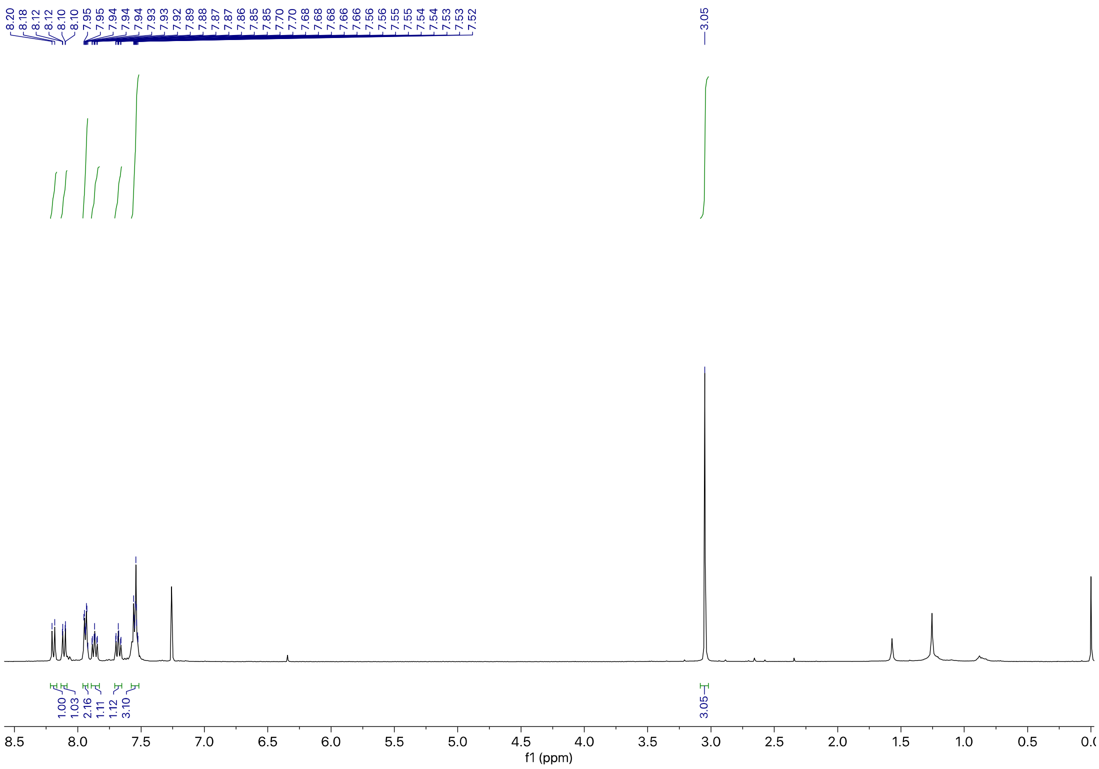

3.6.3. ^{13}C NMR for 4-Methyl-2-phenylquinoline-3-carbonitrile (Table 4, entry 3)

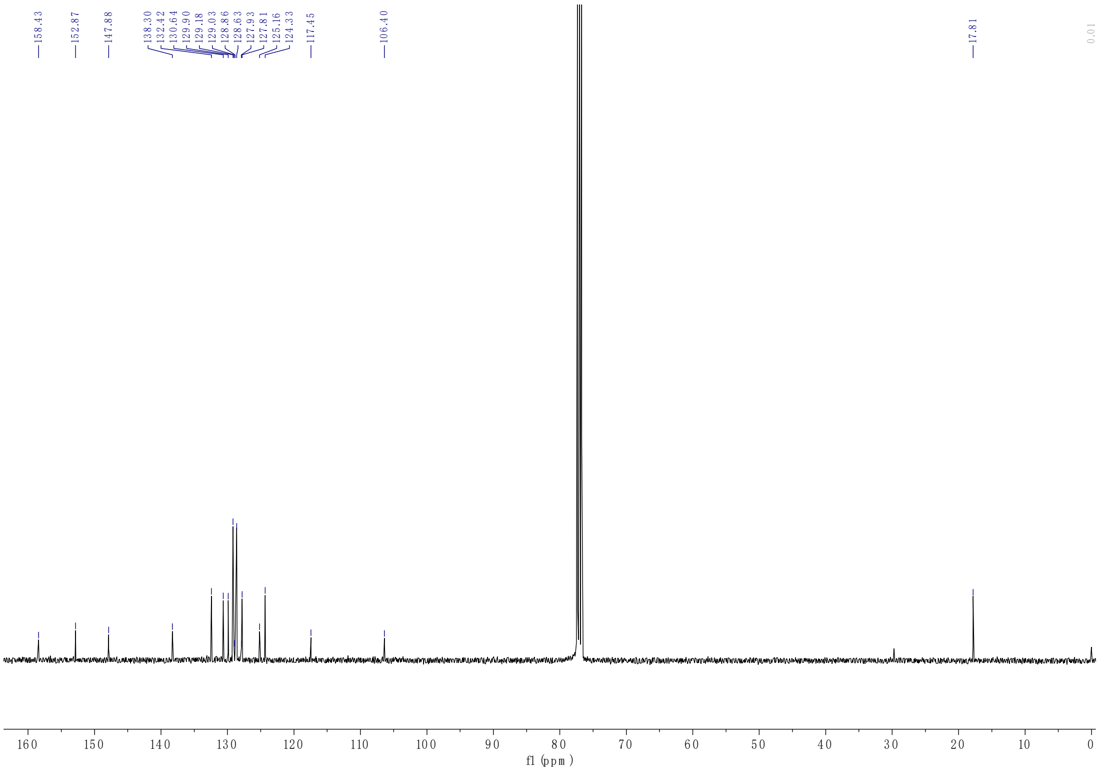

3.6.4. ^{1}H NMR for 1-(2,4-Dimethylquinolin-3-yl)ethan-1-one (Table 4, entry 4)

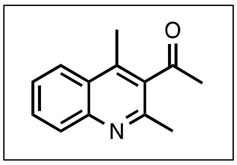

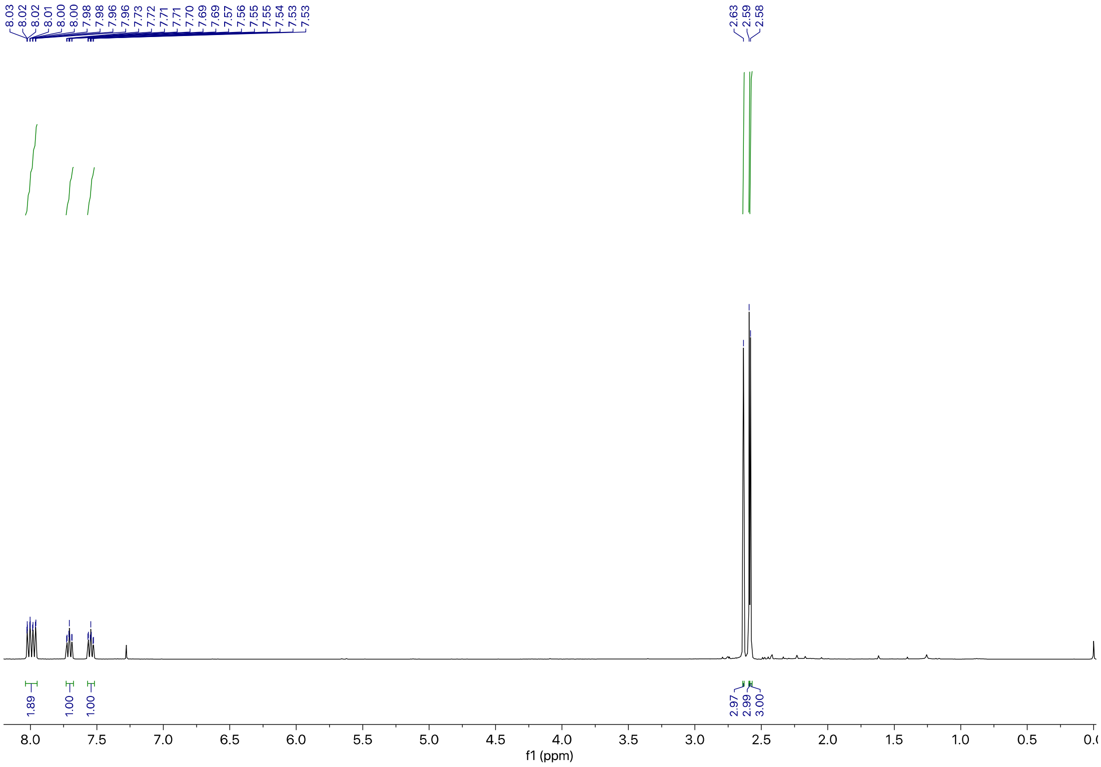

3.6.4. 13C NMR for 1-(2,4-Dimethylquinolin-3-yl)ethan-1-one (Table 4, entry 4)

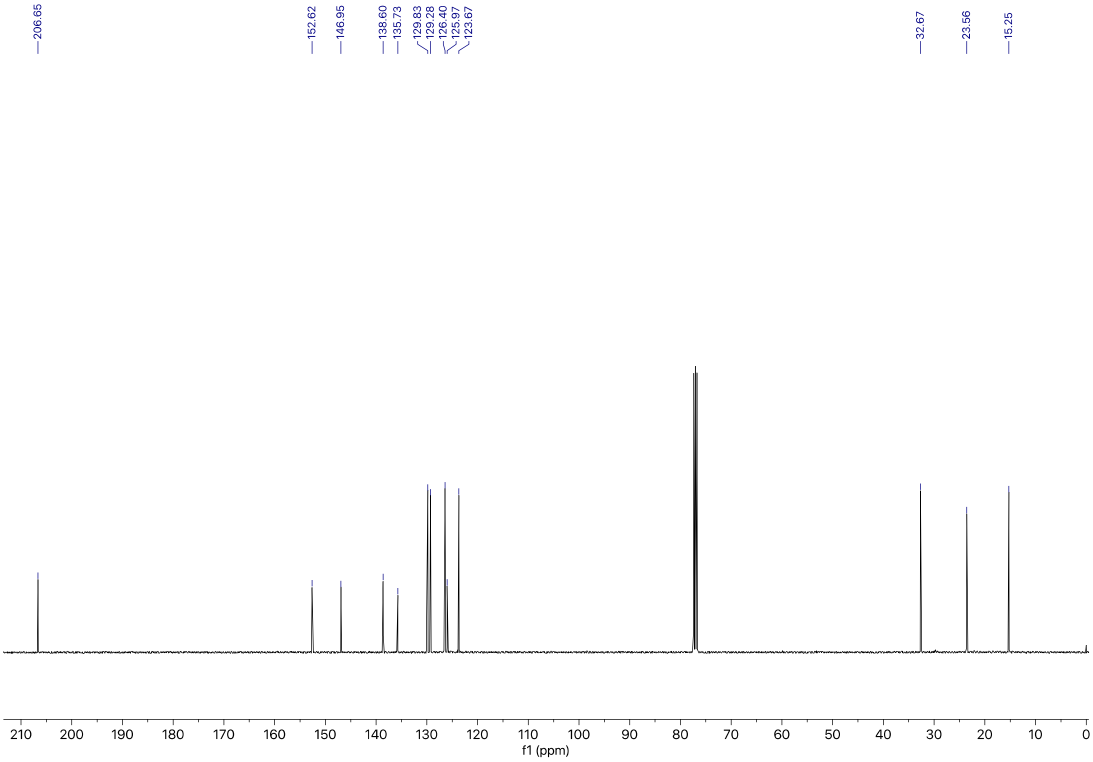

3.6.5. 1H NMR for 3,3,9-Trimethyl-3,4-dihydroacridine-1(2*H*)-one (Table 4, entry 5)

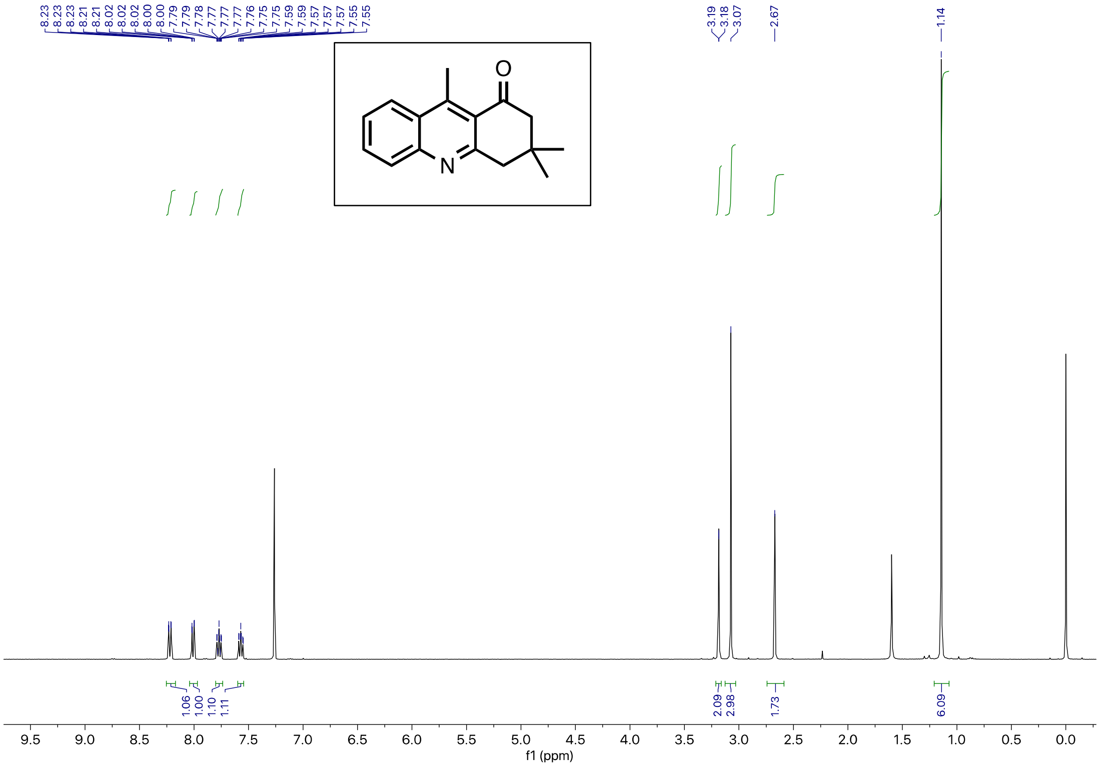

3.6.5. ^{13}C NMR for 3,3,9-Trimethyl-3,4-dihydroacridin-1(2*H*)-one (Table 4, entry 5)

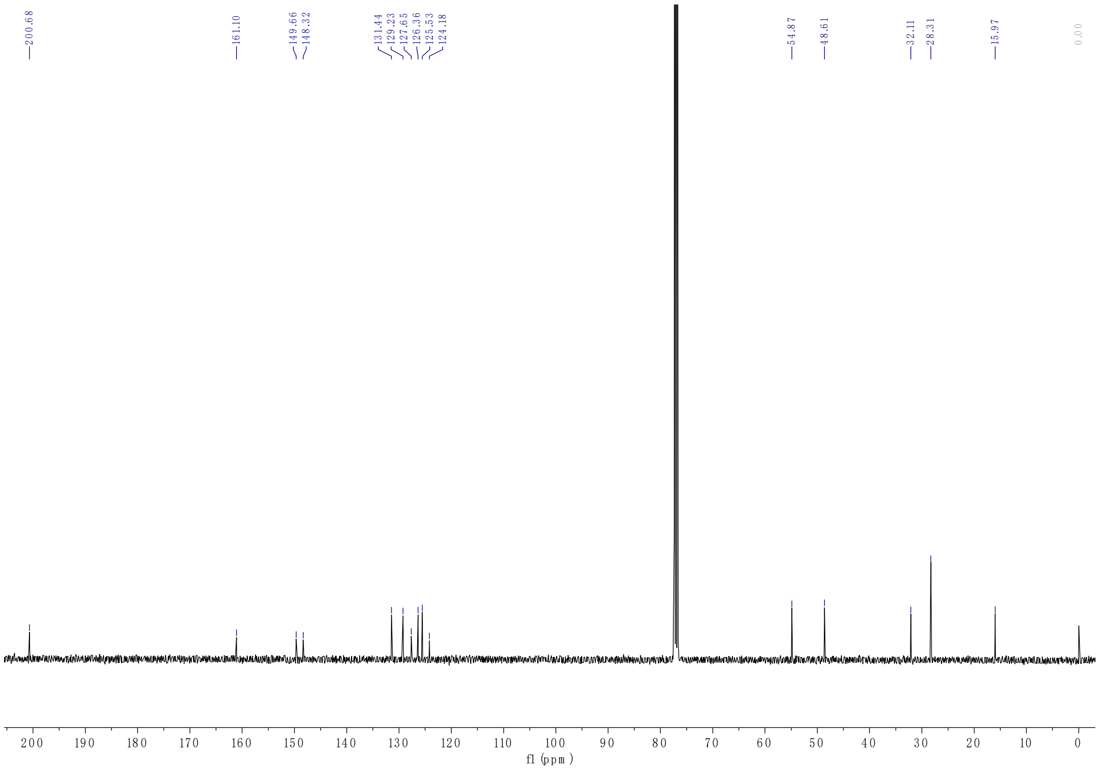

3.6.6. ^{1}H NMR for 2,4-Dimethyl-3-phenylquinoline (Table 4, entry 6)

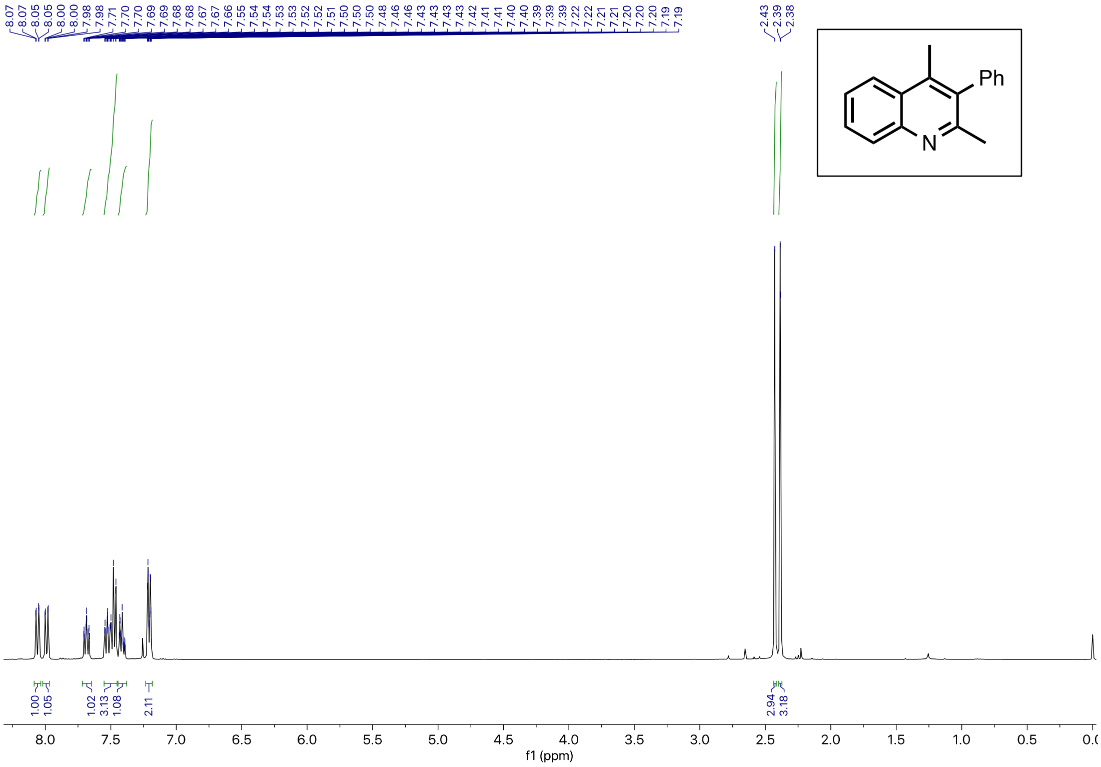

3.6.6. ^{13}C NMR for 2,4-Dimethyl-3-phenylquinoline (Table 4, entry 6)

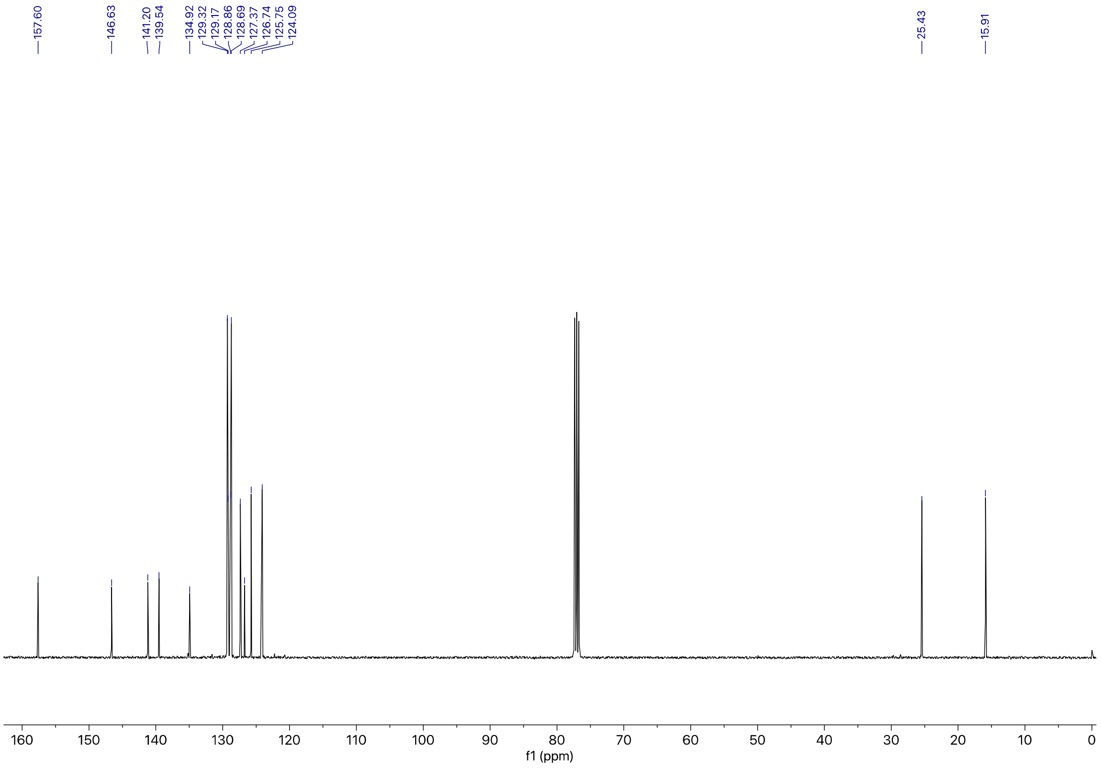

3.6.7. ^{1}H NMR for 12-Methyl-5,6-dihydrobenzo[*a*]acridine (Table 4, entry 7)

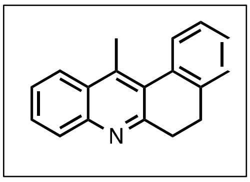

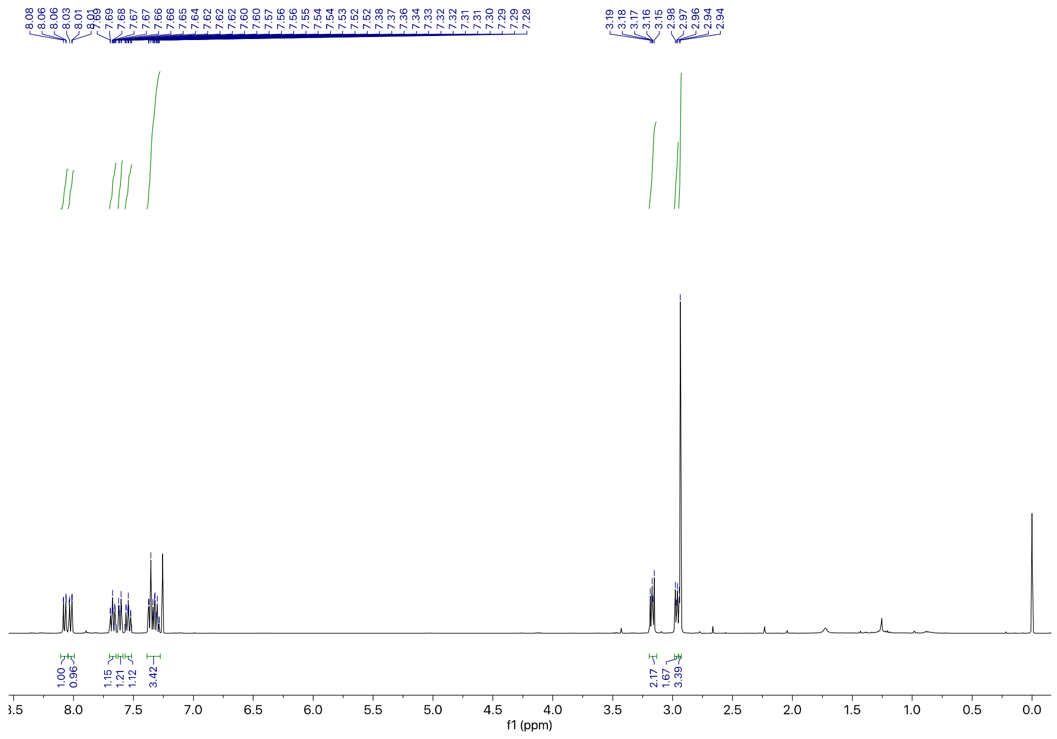

3.6.7. ^{13}C NMR for 12-Methyl-5,6-dihydrobenzo[*a*]acridine (Table 4, entry 7)

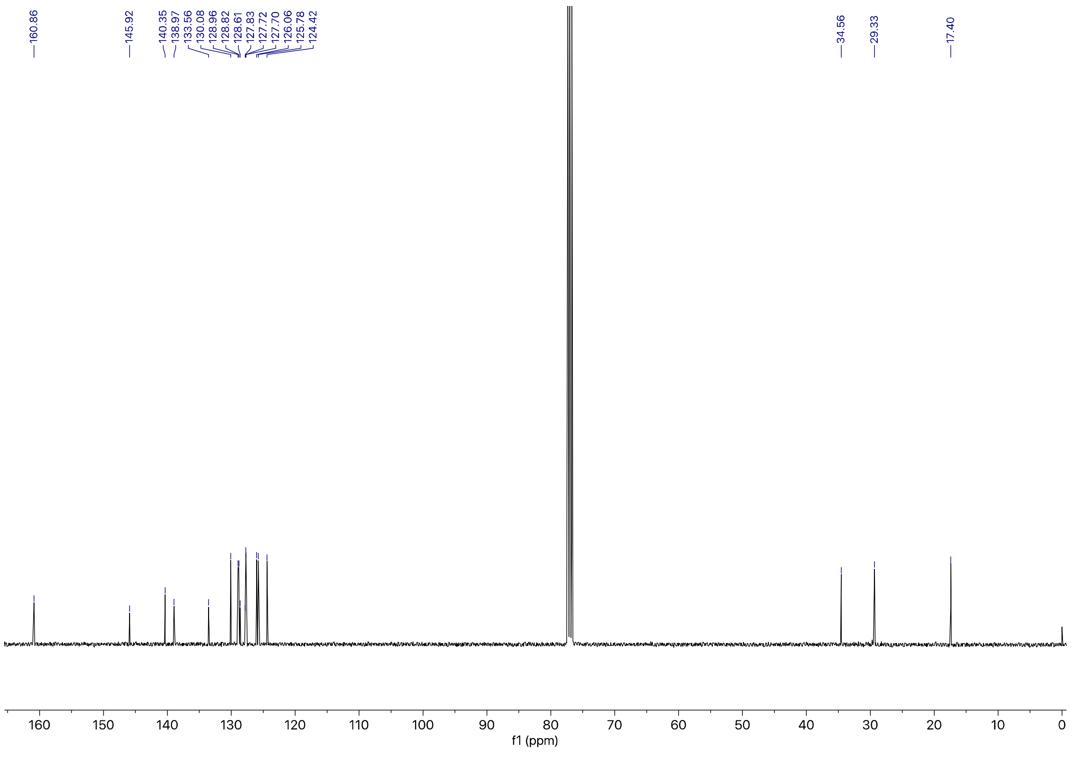

3.7.1. ^{1}H NMR for Methyl 2-ethyl-4-phenylquinoline-3-carboxylate (Table 5, entry 1)

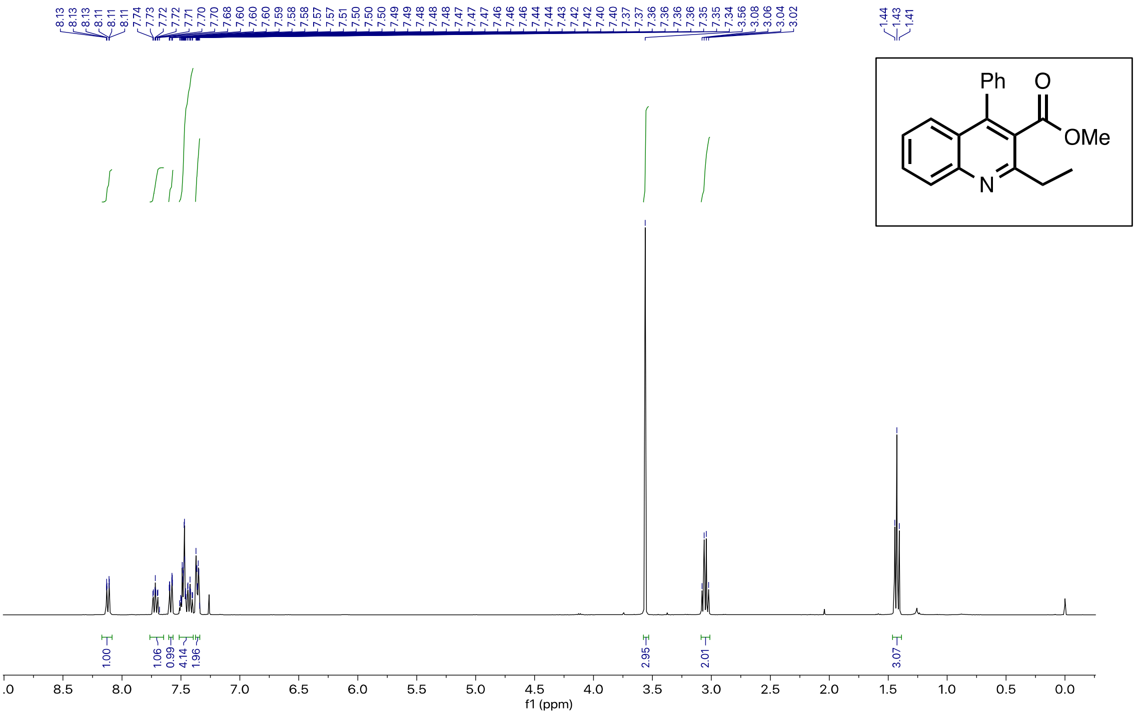

3.7.1. ^{13}C NMR for Methyl 2-ethyl-4-phenylquinoline-3-carboxylate (Table 5, entry 1)

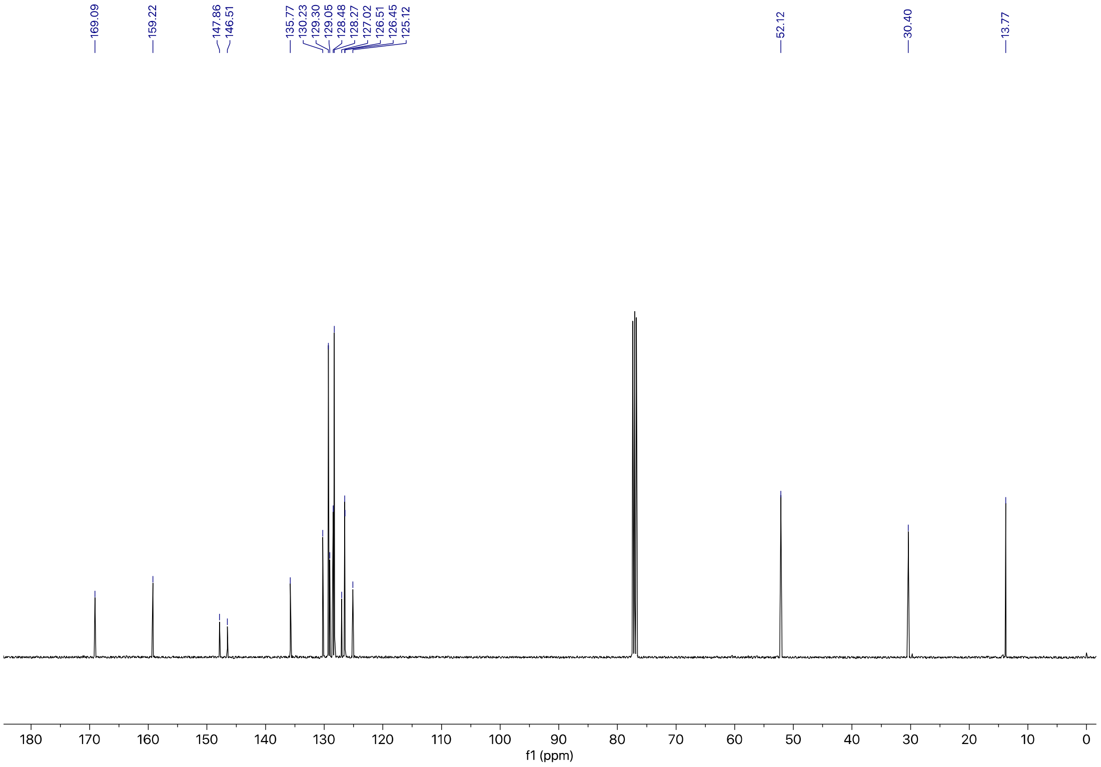

3.7.2. and 3.7.3. ^{1}H NMR for 3-Benzoyl-4-phenylquinolin-2(1*H*)-one (Table 5, entries 2 and 3)

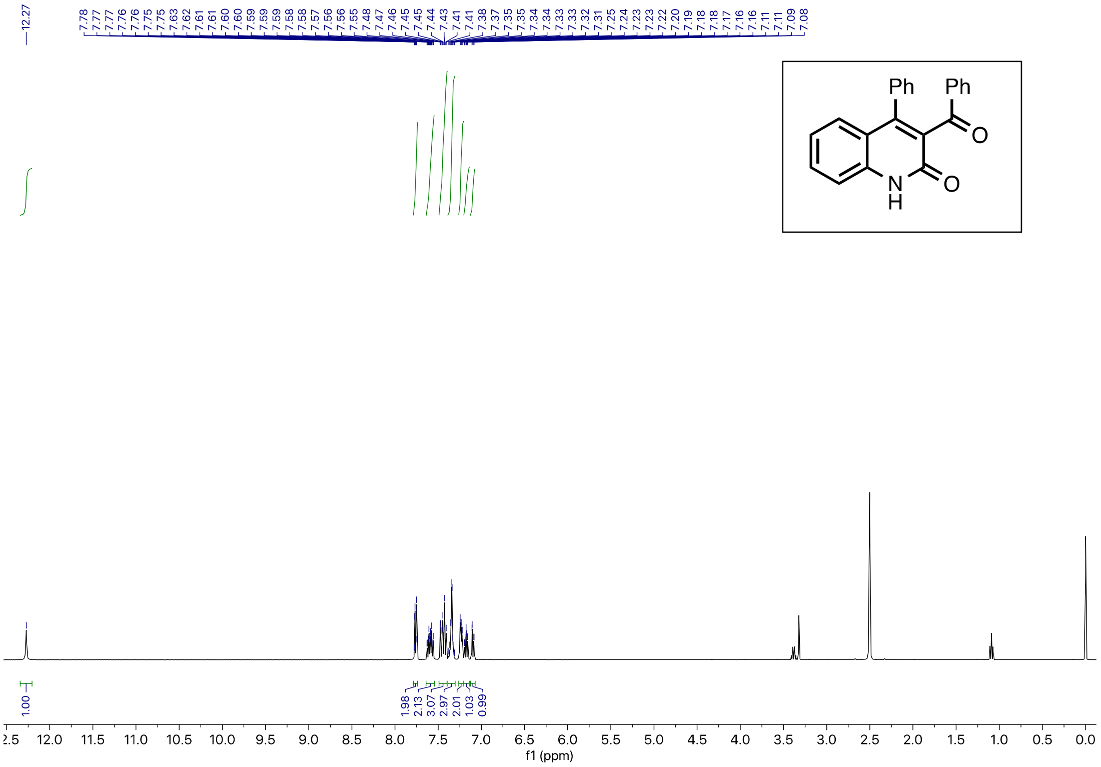

3.7.2. and 3.7.3. ^{13}C NMR for 3-Benzoyl-4-phenylquinolin-2(1*H*)-one (Table 5, entries 2 and 3)

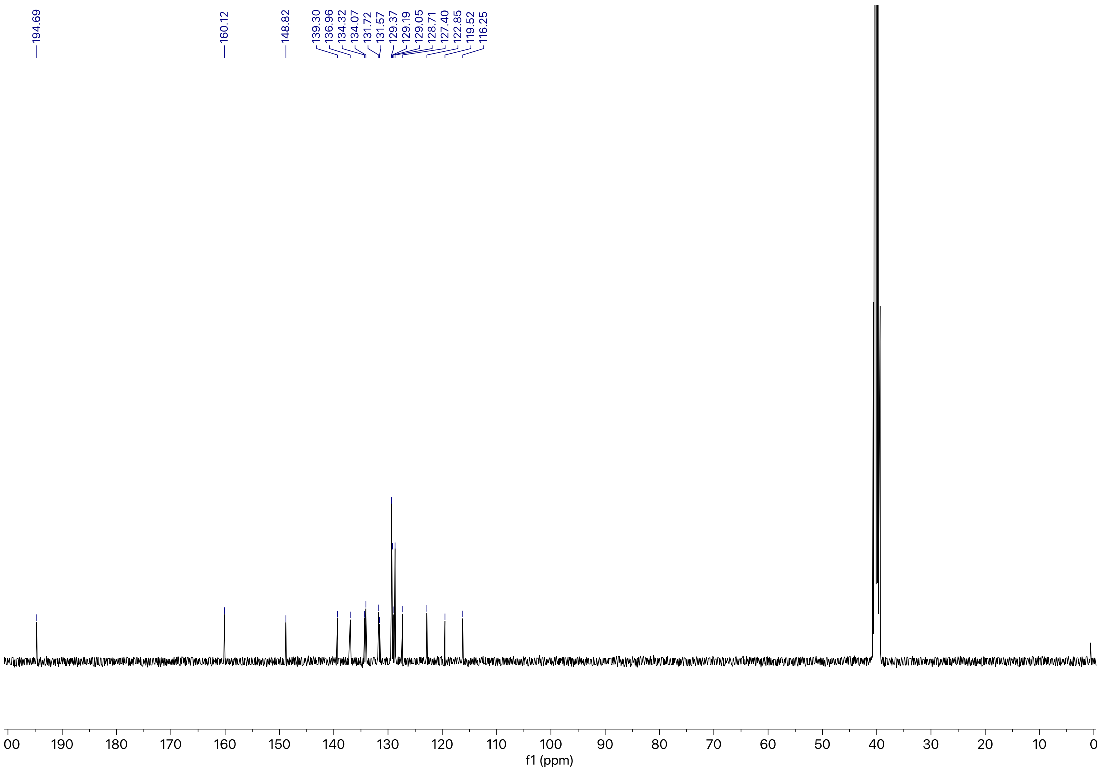

3.7.4. ^{1}H NMR for 1-(2-Methyl-4-phenylquinolin-3-yl)ethan-1-one (Table 5, entry 4)

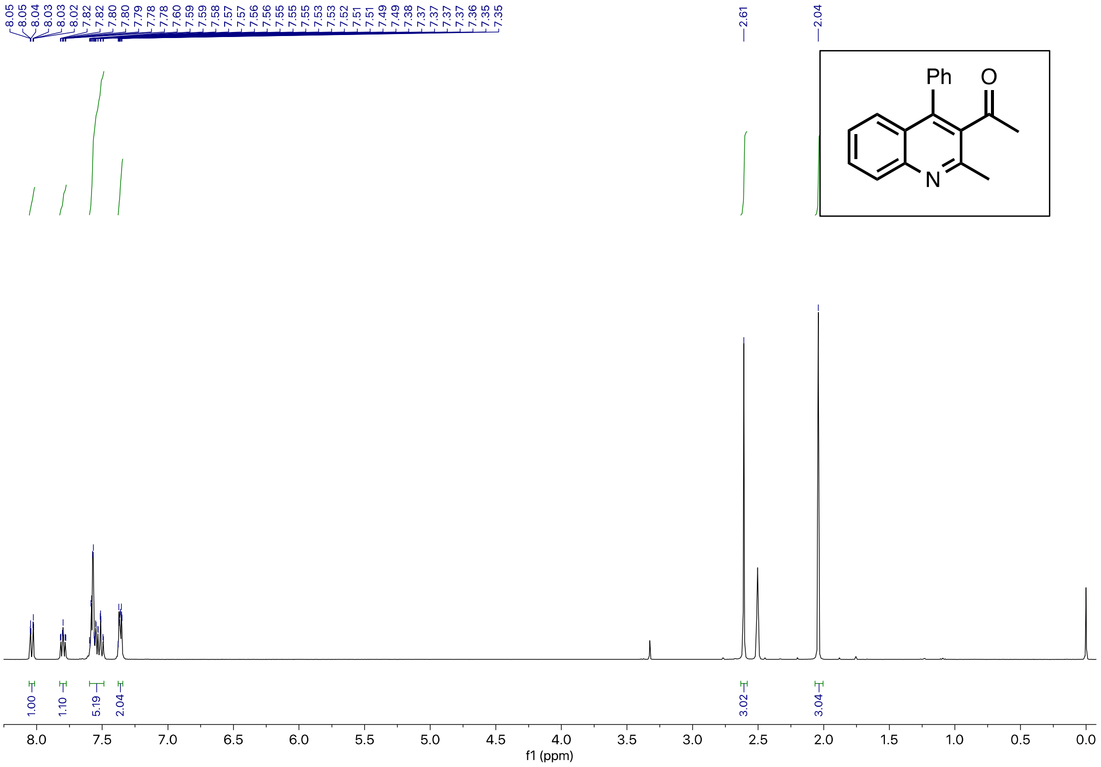

3.7.4. ^{13}C NMR for 1-(2-Methyl-4-phenylquinolin-3-yl)ethan-1-one (Table 5, entry 4)

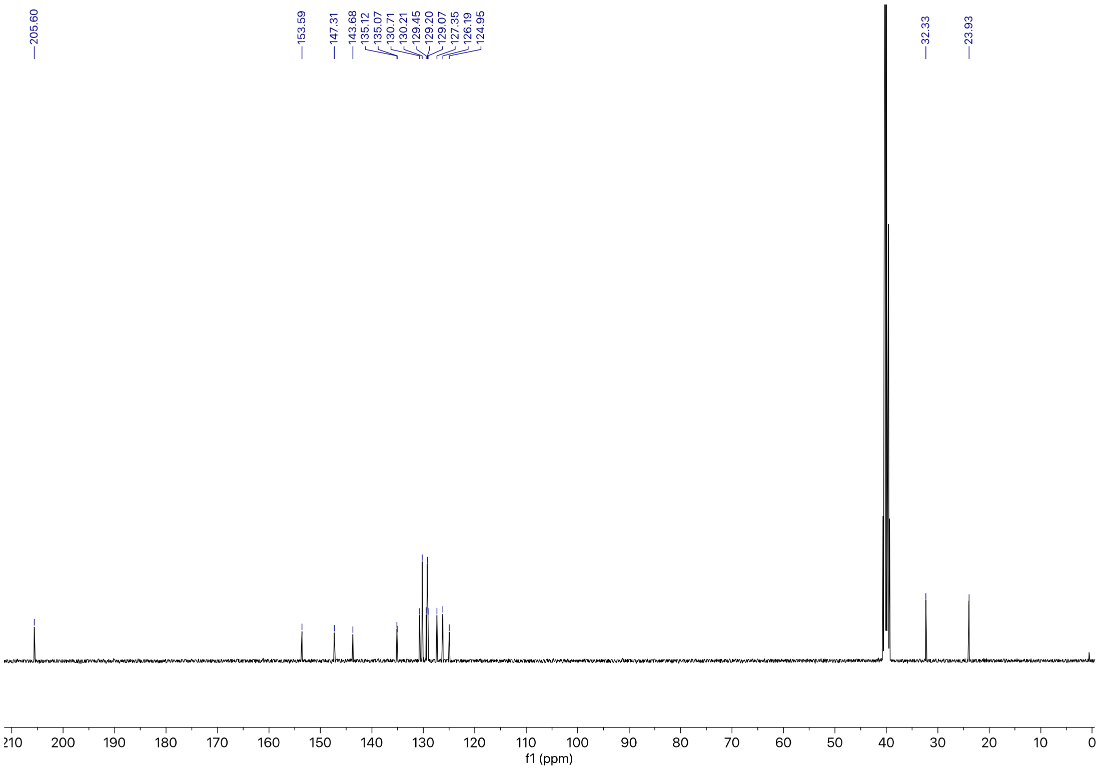

3.7.5. ^{1}H NMR for 3,3-Dimethyl-9-phenyl-3,4-dihydroacridin-1(2*H*)-one (Table 5, entry 5)

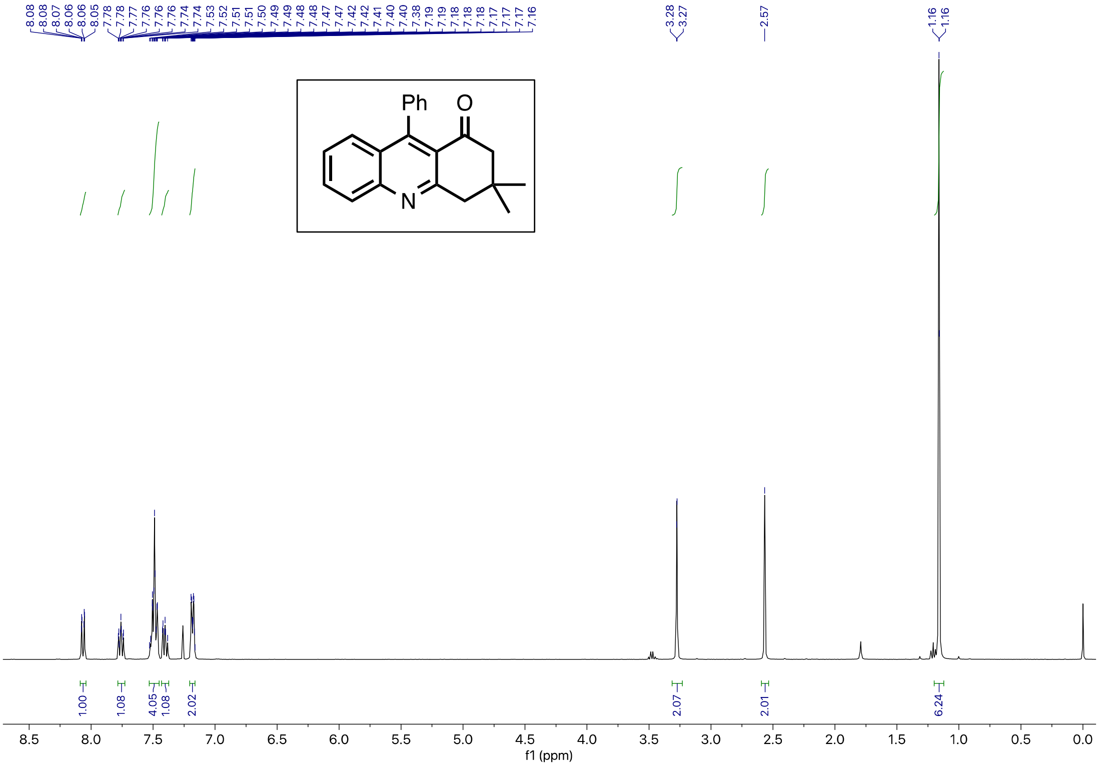

3.7.5. ^{13}C NMR for 3,3-Dimethyl-9-phenyl-3,4-dihydroacridin-1(2*H*)-one (Table 5, entry 5)

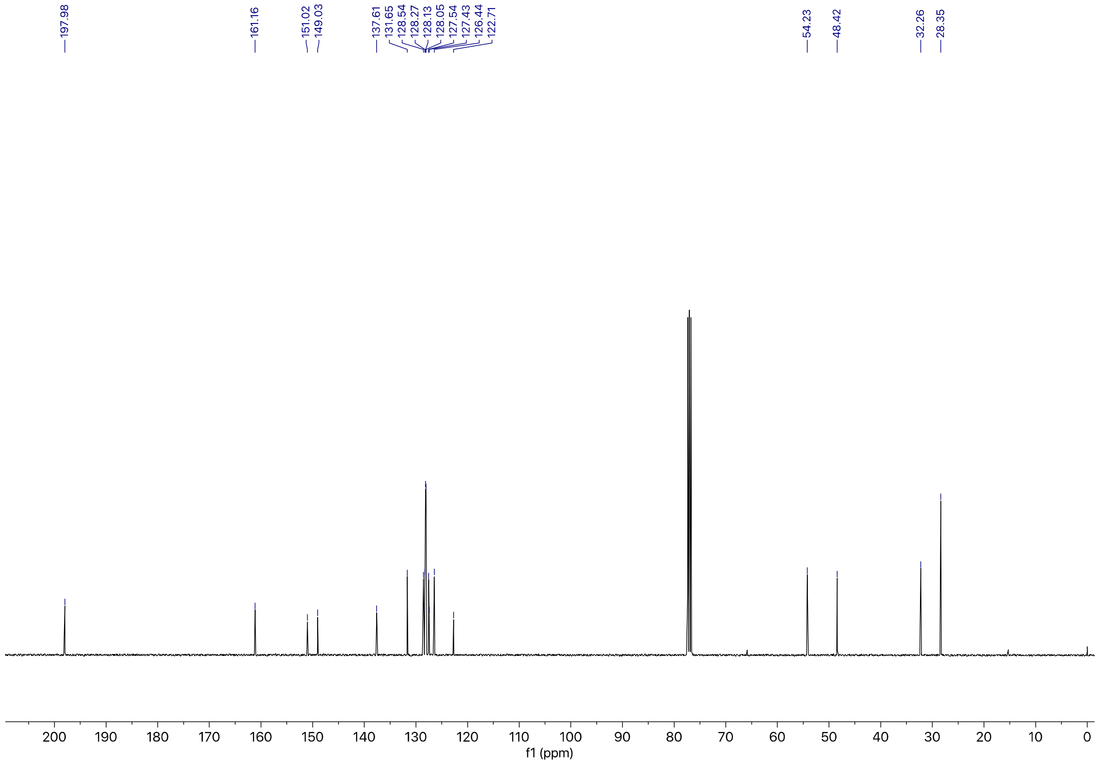

3.7.6. ^{1}H NMR for 2-Methyl-3,4-diphenylquinoline (Table 5, entry 6)

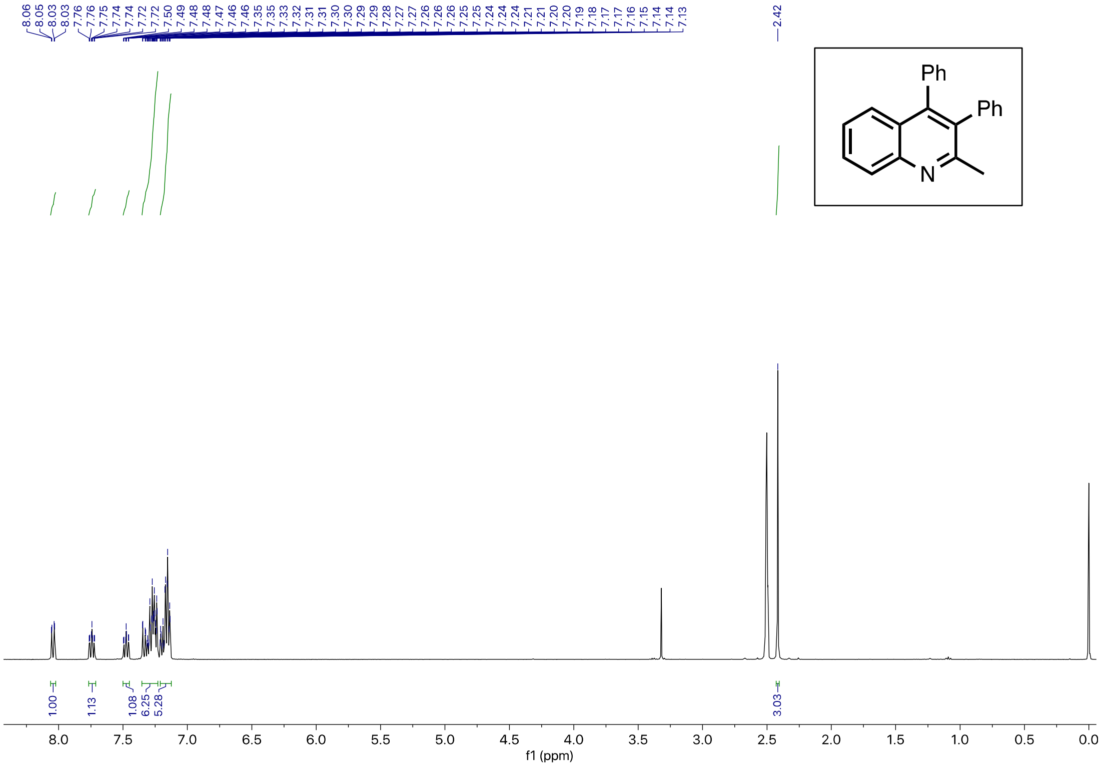

3.7.6. ^{13}C NMR for 2-Methyl-3,4-diphenylquinoline (Table 5, entry 6)

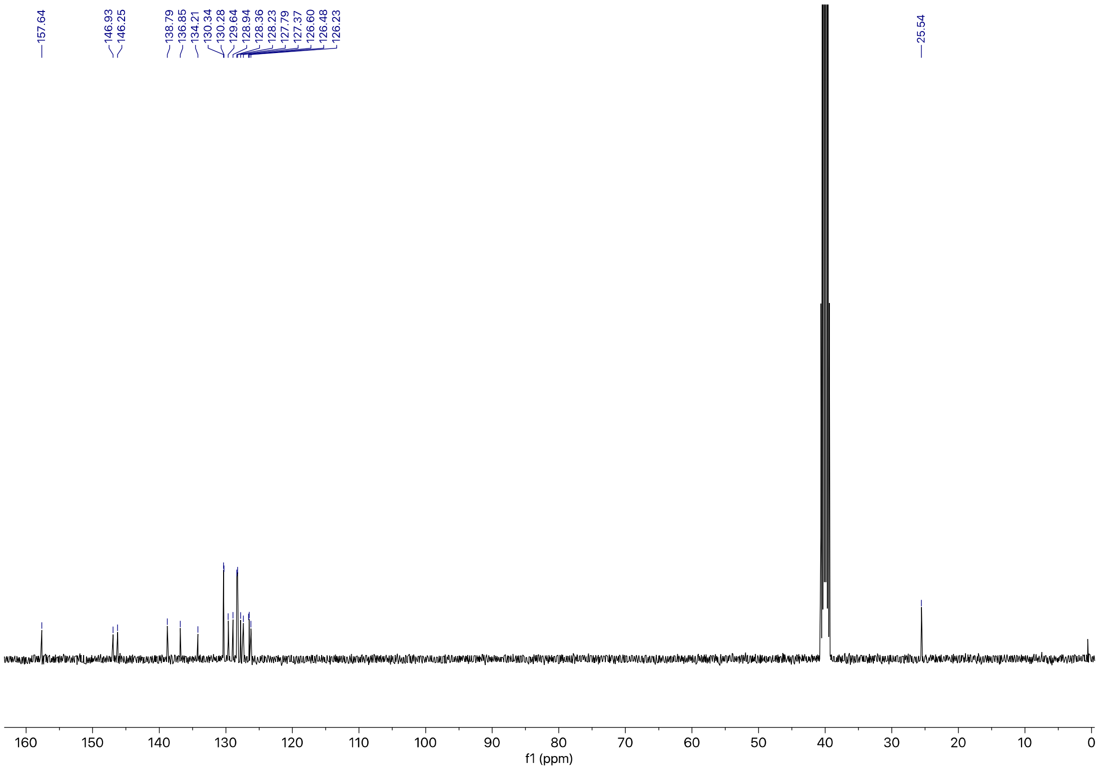

3.7.7. ^{1}H NMR for 3-Butyl-2,4-diphenylquinoline (Table 5, entry 7)

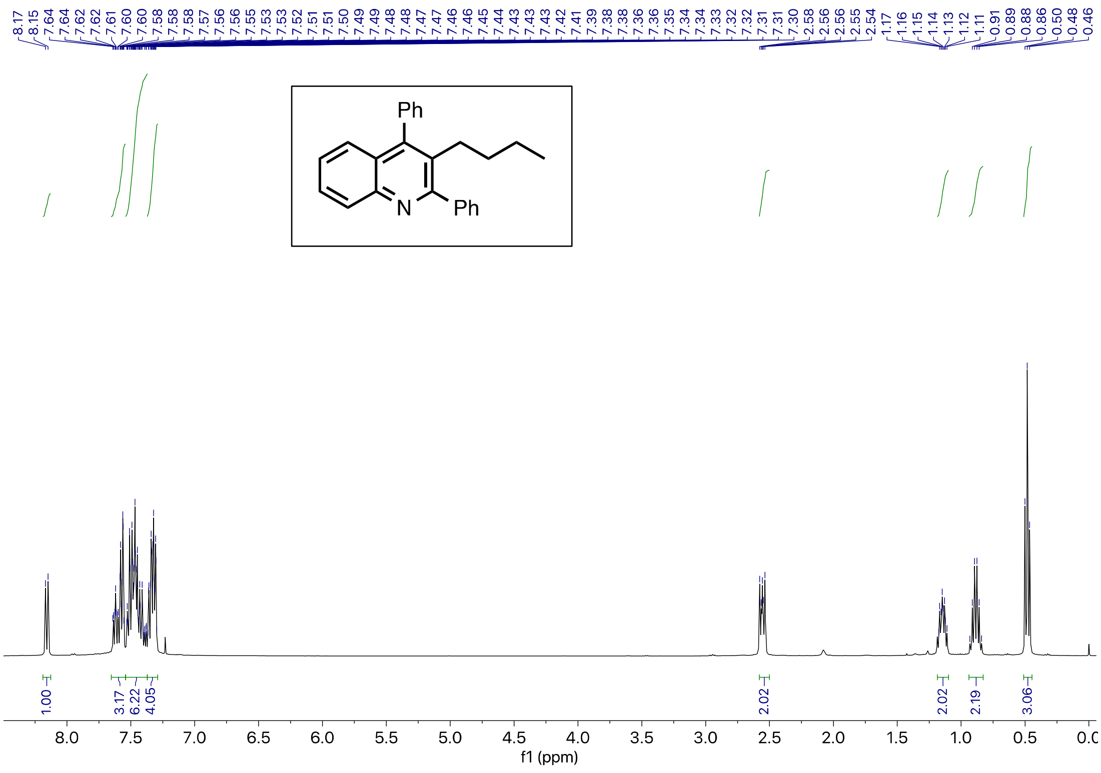

3.7.7. ^{13}C NMR for 3-Butyl-2,4-diphenylquinoline (Table 5, entry 7)

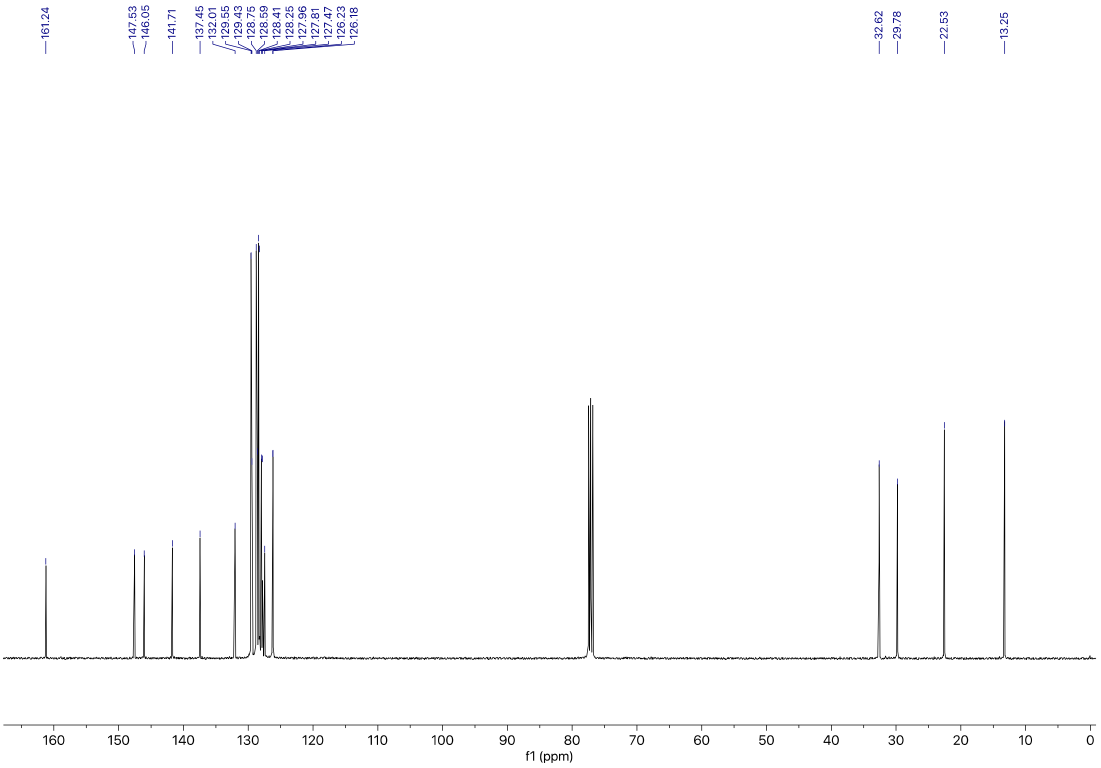

3.7.8. ^{1}H NMR for 12-Phenyl-5,6-dihydrobenzo[*a*]acridine (Table 5, entry 8)

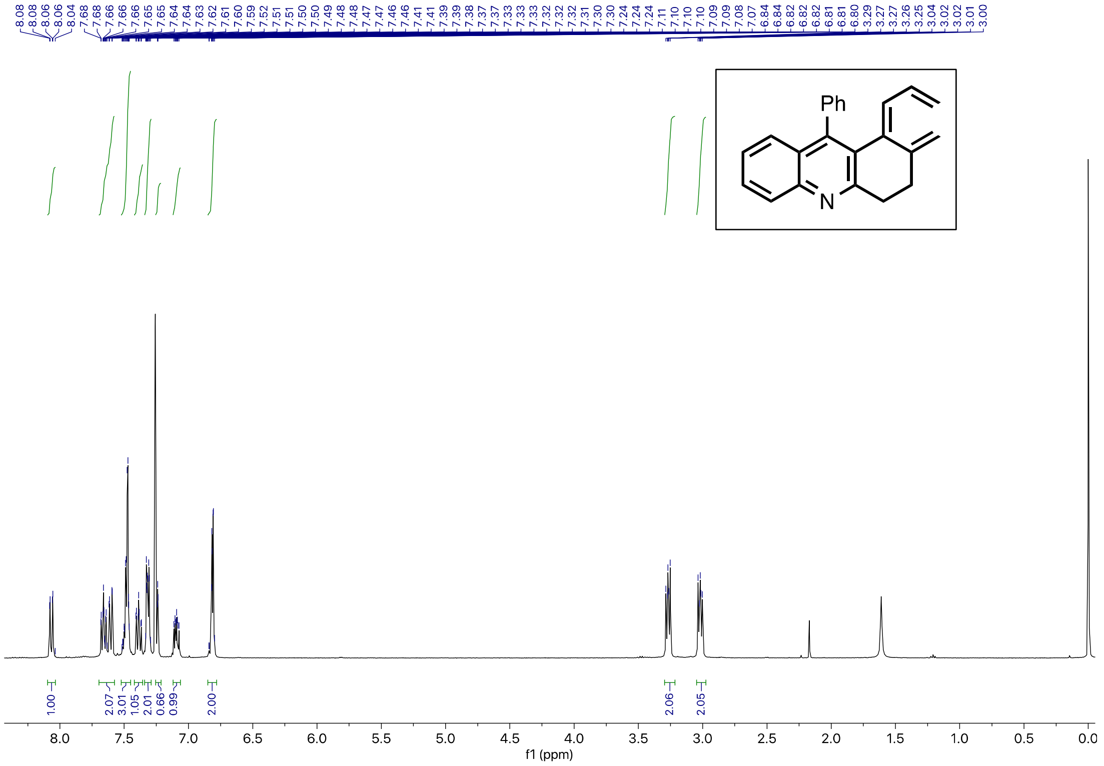

3.7.8. ^{1}H NMR for 12-Phenyl-5,6-dihydrobenzo[*a*]acridine (Table 5, entry 8)

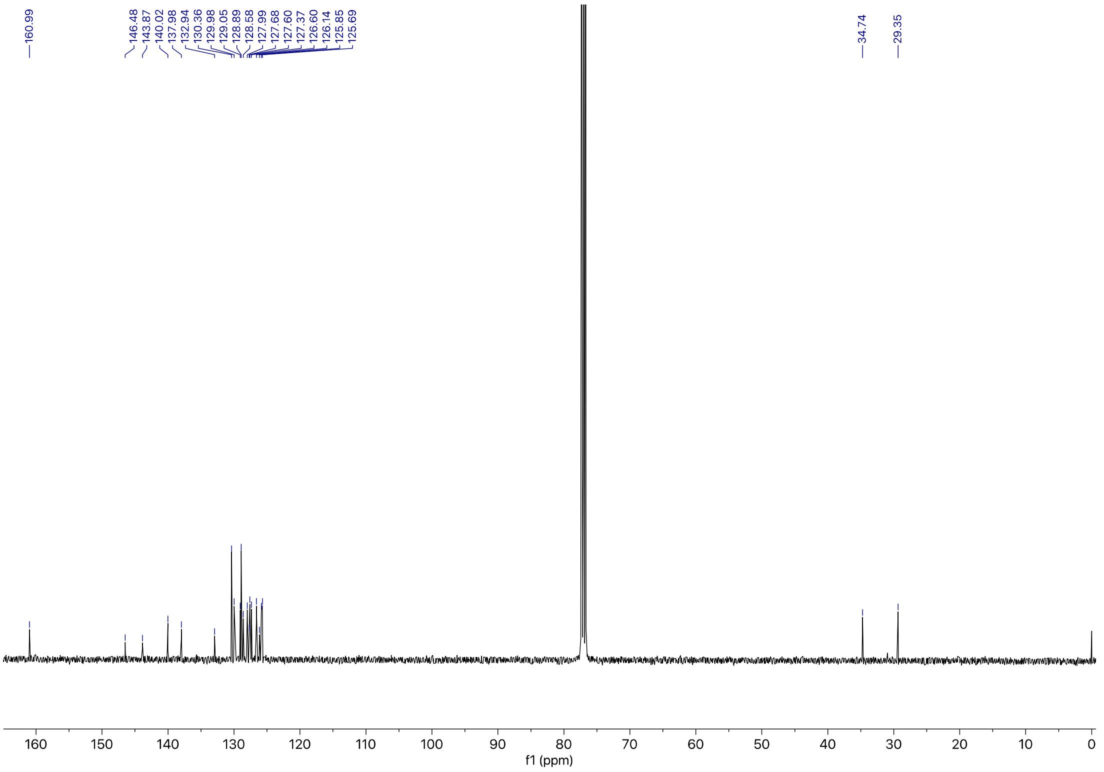

Supplement: Supplementary file 1 [file molecules-27-04123-s001.zip › molecules-1749638-supplementary.pdf]
